# Supplementary material for: Factors that influenced utilization of antenatal and immunization services in two local government areas in The Gambia during COVID-19: An interview-based qualitative study
Source: PLoS One. 2023 Jun 29;18(6):e0276357. doi: 10.1371/journal.pone.0276357 (PMC10309596; doi:10.1371/journal.pone.0276357)
Supplement: S1 File — (ZIP) [file pone.0276357.s001.zip › Supporting information /Respondent 12.docx]

In-depth Interview Questionnaire for MCH service Users

**Introduction and Consent**

Hello, my name is Abdourahman Bah. I am a final year (MRC sponsored) BSc Global Health student at Queen Mary University of London. I am interviewing health workers and mothers in The Gambia to learn about the impacts of Covid-19-related lockdown measures on utilisation of mother and child services. The interview will take about 30 minutes. All the information I obtain will remain strictly confidential. You may choose not to answer any question that makes you feel uncomfortable.

Do you have any questions?

Do you agree to being interviewed? Yes

| **A** |  |
| --- | --- |
|  |  |
| 1 | **Could you please tell me where you live – Probe: house of residence is?**  I am from Brikama Nyambai |
| 2 | **Please tell me how you got here today? Probe: public transport, private or walked.**  I used public transport |
| 3 | **Have you used MCH services during the pandemic? Probe: immunisation, antenatal consultations etc.**  I used to bring my child for immunisation at that time, but there came a time when we bring our children they will not be weighted. |
| 5 | **Have you changed the way you access this service during the outbreak? If so, how? If you have changed, are you going more times or less times?**  I used to come regularly but there were many difficulties that I faced, especially transport difficulties. Many people stopped coming for immunisation services during the pandemic. They were afraid to come because of fear of infection. |
| B | **Individual factors** |
| 7 | **How safe do you think it is to access MCH services during the pandemic? - Probe: have these concerns stopped you from using these health facilities?**  It wasn’t safe to come health facilities during that period, but I still continued bringing my child for immunisation. |
| 8 | **Have you experienced any financial difficulties (e.g., transport costs) in accessing MCH services during the pandemic? if yes, explain.**  Transport was huge difficulty during the pandemic, as it was difficulty to have a vehicle to bring me to the health facility. As such, I had to walk to the health facility from my home. However, despite this difficulty, I continued to bring my child. At that time, they stopped weighting children since there was only one weighing machine that was used for weighing all the children. This was done to reduce the chances of transmitting the disease from one child to another. They were only vaccinating children without weighing them. |
| C | **Interpersonal factors** |
| 9 | **What is your family’s attitude, including your husband, in your use of MCH services during the pandemic? Probe: Do they encourage or discourage you? In what way?**  My husband was always very supportive, and he used to encourage me to bring our child to the health facility. He used to advise me to wear a face mask when coming to the health facility and wash my hands when I get to the health facility. When coming, he would also give me money to pay transport fare. There was no one in my family who discouraged me from coming to the health facility. |
| 10 | **Have you noticed any changes in your friends’ attitudes in use of MCH services during the pandemic?**  Among my friends, there were people who were coming for MCH services regularly and there were some who were not coming for MCH services during the pandemic. I am not very sure why they were not coming, but I believe it is because of fear of the pandemic. |
| D | **Community factors** |
| 11 | **Have you noticed any changes in people’s perception in your community about the use of MCH services during the pandemic? if yes, explain. Probe: give examples of people being afraid of visiting facilities due to stigma associated with visiting health facilities or fear of being quarantined etc.**  Before the pandemic, the hospital used to be over-crowded, but the during the pandemic, the number of people coming to the health facility declined dramatically. I believed this also because of the Covid-19 pandemic. |
| 12 | **Has this had any impact on your use of MCH services during the pandemic? if yes, explain how**  Yes, it disturbed me a lot, but I just continued coming because of the benefit immunisation has for my child. |
| E | **Institutional factors** |
| 14 | **Did the health facilities stay open during the pandemic? if no, state how this may have affected your access to MCH services.**  During the pandemic, I used to come to this health facility. Although the number of people coming here reduced a lot, the hospital remained opened throughout the pandemic. |
| 15 | **How satisfied are you with the care provided by this health facility during the pandemic? probe: consultation time, treatment and respect from health workers.**  I was satisfied with services I received as the waiting time was reduced since there were few people here, as they were all trying to avoid infection at the health facility. There were also social distancing and mandatory wearing of face mask. Anyone without a mask wouldn’t be allowed entry into the health facility. |
| 16 | **Do you think this health facility has adequate medical supplies during the pandemic? if no, give reasons.**  There not enough medicines available, so we had to buy some of them from the pharmacy. Shortage of medicines here normal as it that is the case here even without the Covid-19 pandemic. |
| 17 | **Do you think this facility has enough manpower to provide MCH services during the pandemic? if no, give reasons**  When I come here, I used to find health workers here, but I don’t know if they were enough or not. |
| 18 | **What are your perceptions about the health workers in this facility? (e.g., competence or behaviour of health workers)**  I haven’t had any problems with the health workers here and I haven’t seen them have an issue with anyone. They were doing their work properly, as they provided me with all the services that I needed. |
| 19 | **How safe do you think it is for women to currently access MCH services during the pandemic in this health facility? Please explain.**  I think it is now safe to come to health facilities. I see that the number of people coming for MCH services has now gotten back to pre-Covid-19 levels. |
| F | **Policy factors** |
|  | **Was there any other barrier to accessing health care services during the pandemic that I did not ask you about?**  Transport to health facilities was a big difficulty for me. When sometimes I forgot my face mask at home, I would have to buy one, which was a burden for me. This is because a mask is expensive and sometimes, I don’t have much money on me. Even if I explain my situation do the security guard at the gate, he will not allow me to enter without a face mask. |
| 22 | **Have these measures had any impact on your use of MCH services during the pandemic? if yes, explain how**  These measures did not have any effect in my use of MCH services. I know they were introduced to protect every from the disease. However, I know some people may have Asthma. As such, they cannot wear face mask as it affects their breathing. For such persons, the mandatory wearing of face mask in health facilities may prevent them from coming. |
| 23 | **What do you think the government should do to prevent a decline in use of MCH services in the event of another pandemic?**  May God forbid, if there happens to be another wave of Covid-19. I would ask them to financially support us, since most Gambians are living below the poverty line. The government should also educate people about the pandemic. |
| 24 | **What advice would you give to people who were not using MCH services during the pandemic?**  I would advise them to bring their children for immunisation because it is important of the health of their children |
