# Supplementary material for: Factors that influenced utilization of antenatal and immunization services in two local government areas in The Gambia during COVID-19: An interview-based qualitative study
Source: PLoS One. 2023 Jun 29;18(6):e0276357. doi: 10.1371/journal.pone.0276357 (PMC10309596; doi:10.1371/journal.pone.0276357)
Supplement: S1 File — (ZIP) [file pone.0276357.s001.zip › Supporting information /Health worker 8 .docx]

In-depth interview questionnaire for health workers

**Introduction and Consent**

Hello, my name is Abdourahman Bah. I am a final year (MRC sponsored) BSc Global Health student at Queen Mary University of London. I am interviewing health workers and mothers in The Gambia to learn about the impacts of Covid-19-related lockdown measures on utilisation of mother and child services. The interview will take about 30 minutes. All the information I obtain will remain strictly confidential. You may choose not to answer any question that makes you feel uncomfortable.

Do you have any questions?

Do you agree to being interviewed? Yes

| **Background** |
| --- |
| 1. **Could you please tell me where you live?**   I live in Sukuta   1. **What is your profession?**   I am a nurse.   1. **What does your role entail?**   As a nurse, my role involves attending to pregnant women. This involves checking their vitals, taking their weight and giving them medications. |
| 1. **Please tell me for how long you have been working in this health facility?**   I have been working here for sixteen years now. |
| 1. **What motivated you into pursuing a public health career?**   I chose nursing as a career because I want to help people. This what motivated me into pursuing a career in nursing. |
| 1. **What MCH services are provided in this facility? Probe: immunisation, antenatal care**   In this health facility, we provide immunisation services and antenatal services, but in this department, we attend to pregnant women and sometimes attend to children who are sick.   1. **Did the provision of these services continue during the pandemic?**   During the Covid-19 pandemic, we continued to provide all these services. We didn’t stop providing a single service. The antenatal service was running as usual. The only difference was that we made sure that when coming to the health facility, they follow the precautionary measures. |
| 1. **Did the health facility stay open during the pandemic, and for how long?**   This hospital was always open and was never closed during the pandemic. The only days that we don’t come to work was on public holidays. We used to come to work every day. |
| 1. **Have you noticed any changes in utilisation of MCH services during the pandemic? For example, do you see fewer or more patients than usual?**   In this department, the number of women coming for antenatal services did not decline as they continued to come as normal. |
|  |
| **Individual factors** |
| 1. **From the perspective of health workers, how safe do you think it is to provide MCH services during the pandemic?**   It was difficult for us. As you know, people are different. Some would come following the precautionary measures while others you have to tell to wear a face mask. Even if they happen to put on the mask, they remove as soon as leave. That was a big difficulty for us. When coming into the health facility, the security guards made sure that they wear a face mask and wash their hands. They don’t allow anyone who refuses to follow these measures to get into the health facility. However, we knew that some may have some health problems, such Asthma, for such individuals we would ask them to get a face shield since that does not hinder their breathing. |
| 1. **How safe is for women to access MCH services in this facility at that period?**   To be honest, it was not safe for them, as they can easily get infected. To protect, we used to ask them to observe social distancing by keeping a safe distance between each other, but we have now stopped doing that because we don’t have enough space and enough chairs. At the moment, we emphasise the wearing of face mask and hand washing. |
| 1. **Did you or your colleagues work more or less hours during the lockdown? If yes, please explain why?**   In this health facility, our workload was not reduced as we were coming to work every day. Sometimes, instead of going home at 2:00 pm, we would stay here until around 3:00 to 4:00 pm because of the high number of patients. This was the case during the Covid-19 pandemic as they continued to come as normal. |
| **Interpersonal factors** |
| 1. **What is your family’s attitude in your provision of MCH services during the pandemic? (Are they supportive or not? If yes, explain how?**   I didn’t have any difficulty from my family because when I come to work, I make sure that when I get home, I wash my hands. I was lucky not get infected during the pandemic, but I was scared that I may get it and take it to my family. I understand that some of my colleagues were stigmatised in their communities because of their work, but for me, I did not experience that. It was just my brother’s wife who used to tease me. |
| 1. **Have you noticed any changes in your colleagues’ attitudes in providing MCH services during the pandemic? probe: did you experience a reduction in staff’s work appetite? If yes, explain why (maybe due to lack of risk allowance and patient overcrowding)**   In this department, we were all motivated to provide antenatal service.   1. **What incentives were provided by the government to motivate health workers during the pandemic?**   The incentive we received from the government was last year during the start of the pandemic. We were given some money, but even that wasn’t much and that was done just once. The lack of regular incentives from the government did not de-motivate us and didn’t prevent from coming to work and provide antenatal service. |
| 1. **What is your attitude towards MCH service users during the pandemic? probe: were they making your work easier or more difficult?**   We used to have many difficulties with the patients, especially with regard to the wearing of face mask. When entering the hospital, they are asked to put on the mask, but as soon as they go past the security guards, they remove it. Some of them did not like the idea of wearing a face mask at all. |
| **Community factors** |
| 1. **Have you experienced any changes in people’s perception in the community about the use of MCH services during the pandemic? if yes, explain.**   Yes, I used to hear people saying that I will not go the health facility because of the Covid-19 pandemic. |
| 1. **Have you experienced any challenges in providing MCH services due to transport difficulties? if yes, explain how**   Yes, I used to have transport problems because at that time, I was using public transport and some of the passengers refuse to put on a face mask. This is because they do not believe Covid-19 is real. Most of the drivers were also not going to work, so there was vehicle shortage and fares were increased as well. |
| **Institutional factors** |
| 1. **What do you think of the quality of care provided by this health facility during the pandemic?**   Our quality of service was not affected as we continued to provide the service as usual. Our only problem was that some patients were not willing to follow the precautionary measures. Despite this hinderance, we continued to do our jobs. |
| 1. **Do you think this health facility had adequate medical supplies during the pandemic? if no, give reasons.**   We did not have a shortage of medical supplies that are needed in this department, as we had enough stock at the pharmacy. I don’t know about other departments, but in this department, all the medicines that pregnant women need were continuously available.   1. **Do you think this health facility had adequate PPEs during the pandemic? if no, give reasons. Did that have any effect on your willingness or ability to provide MCH services?**   We used to be supplied with PPEs but there used to be a time when their availability used to be a problem. If it would be possible, we would be happy to be supplied with more PPEs because we currently use the same face mask for three days. The supply of PPEs is not meeting the level of demand for them. This is because there is a high demand for PPEs, especially for face mask. |
| 1. **Do you think this facility had enough manpower to provide MCH services during the pandemic? if no, give reasons**   We used to have manpower shortage as some of our colleagues got infected, but thank God, they are all feeling well now and have come back to work. The shortage of manpower impacted our ability to provide antenatal service as we had to cover for those got infected and went into quarantine. The workload was very high as the number of people coming for antenatal care was much higher than the number of nurses on the ground.   1. **What do you think of the health facility environment? Probe: is the facility clean and not overcrowded?**   The environment was not conducive, but we could not stop providing the service. We just managed to provide the service, but it was very difficult for us and for the patients as well. We just tried to help other and make everyone’s lives easier. |
| **Policy factors** |
|  |
| 1. **To prevent infection in health facilities, infection prevention and control measures, such as mandatory screening, wearing of PPEs and face mask, have been introduced in many health centers. What is the effect of these practices on utilization of MCH services?** |
| For some people who don’t like wearing of face mask, it is possible that the fact that they have to put on a face mask when they come to the health facility, they may refuse altogether to come for antenatal service, but in this facility, if you explain your condition to the health workers, they do give exceptions. |
| 1. **Are there any other factors that may have negatively impacted your ability to provide MCH services during the pandemic that I haven’t asked you about? if yes, please state them and explain how?**   The main difficulty we had during the pandemic was the issue of wearing face mask by patients. We were always worried that we will get infected and take the disease to our families because some patients were not willing to follow the precautionary measures. For me, personally, this was big problem. Sometimes I could not even sleep at nigh, especially when I do at test and awaiting the results. Sometimes I would even ask my children to stay away from me because of the fear that I may infect them.   1. **To prevent the decline in use and provision of MCH services in the event of another pandemic or second wave, what do you think the government should do?**   If it is possible, the government should provide us with more face mask and other protective equipment, such as hand sanitisers. They should also provide us with some incentives if it is possible. To encourage women to come for antenatal services, the government should sensitise them and tell them the health facilities are still open and were never closed, as some are not coming to health facilities because they believe that they are closed. This can be done on the radio. They should also be sensitised on the benefits of following the precautionary measures.   1. **What advice would you give to people who are not using MCH services during the pandemic?**   My advice to them is that, if either you or your child happen to get sick, you should not stay at home and not come to the health facility because you cannot cure yourself or your child at home. You should just follow the precautionary measures and come to the health facility. Others were not coming to the health facility because they would say that they would not be allowed to enter the health facility, but that is not the case. If you follow the precautionary measures, you will be allowed entry into the health facility and get the treatment you or your child needs. |
