# Supplementary material for: Factors that influenced utilization of antenatal and immunization services in two local government areas in The Gambia during COVID-19: An interview-based qualitative study
Source: PLoS One. 2023 Jun 29;18(6):e0276357. doi: 10.1371/journal.pone.0276357 (PMC10309596; doi:10.1371/journal.pone.0276357)
Supplement: S1 File — (ZIP) [file pone.0276357.s001.zip › Supporting information /Health worker 11.docx]

In-depth interview questionnaire for health workers

**Introduction and Consent**

Hello, my name is Abdourahman Bah. I am a final year (MRC sponsored) BSc Global Health student at Queen Mary University of London. I am interviewing health workers and mothers in The Gambia to learn about the impacts of Covid-19-related lockdown measures on utilisation of mother and child services. The interview will take about 30 minutes. All the information I obtain will remain strictly confidential. You may choose not to answer any question that makes you feel uncomfortable.

Do you have any questions?

Do you agree to being interviewed? Yes

| **Background** |
| --- |
| 1. **Could you please tell me where you live?**   I live in New Yundum   1. **What is your profession?**   I am a nurse midwife   1. **What does your role entail?**   As a midwife, I conduct deliveries and take care of pregnant women who come for antenatal services. |
| 1. **Please tell me for how long you have been working in this health facility.**   I have been working in this hospital for eleven years now. |
| 1. **What motivated you into pursuing a public health career?**   Being a midwife is part of upgrading yourself because you cannot remain as a general nurse. If you want to upgrade yourself, you will need to do another degree or other courses. This is why I went for the midwifery course. |
| 1. **What MCH services are provided in this facility? Probe: immunisation, antenatal care**   We have the antenatal services, postnatal services, delivery services, and immunisation for their first doses. This is normal given to babies after delivery, usually within twenty-four hours. So, they usually take that one here.   1. **Did the provision of these services continue during the pandemic?**   We continued providing these services during the pandemic but not as before. We tried to reduce the number of patients as much as possible. We were not admitting many patients at that time. This was done to make sure that there was no overcrowding. We asked women to go to their nearby health facilities instead of coming here so as to avoid many people coming here and making the place overcrowded. It was the hospital that introduced this policy so as to minimise the number of patients coming to this health facility. |
|  |
| 1. **Have you noticed any changes in utilisation of MCH services during the pandemic? For example, do you see fewer or more patients than usual?**   There were not many patients coming for MCH services at that time. they were not coming so as to avoid overcrowding |
|  |
| **Individual factors** |
| 1. **From the perspective of health workers, how safe do you think it is to provide MCH services during the pandemic?**   Even if it is not safe, you cannot stop providing the service because you cannot just leave the patients without attending to her needs. So, you have to render the service no matter how hard it is. |
| 1. **How safe is for women to access MCH services in this facility at that period?**   In my opinion it was safe because we tell them the precautionary measures to take so that we can attend to them. The precautionary measures include regular hand washing, wearing of face mask, and social distancing. We don’t allow many of them to gather at the same place. You the health worker, must also make sure that you put on your mask and the patient must also put on their mask. |
| 1. **Did you or your colleagues work more or less hours during the lockdown? If yes, please explain why?**   The number of staff on a shift was reduced. If you work for a day, you have to rest for two days. This was done to reduce the number of staff working at the same time. The staff providing the service was divided into three teams. Today this team will come and have two days of rest. The other team will come the following day and have two days of rest so that you cannot get in contact with each other. That is how we were operating during the pandemic. I can say we worked more time during that period because you start your shift at 8 am and finish at 8 pm, but you also have two days of rest. The workload was a bit reduced because not many patients were coming. For instance, if you deliver here without any complications, we ask you to go your nearest health facility. The number of deliveries we were conducting did not change because when it comes to labour you cannot stop it. So, when women are in labour, they just come here, and we cannot tell them to go to another health facility when they are in labour. This health facility is also a referral hospital, where other health facilities send their patients. There was a time when they wanted to make this facility a Covid-19 centre but didn’t materialise because people were still coming here. |
| **Interpersonal factors** |
| 1. **What is your family’s attitude in your provision of MCH services during the pandemic? (Are they supportive or not? If yes, explain how?**   The pandemic actually started while I was on confinement leave, but when it was time to get back to work, it became very difficult for me. my family was complaining but I told them this is what I signed up for, so I have to get back to work no matter what the situation is. My family was definitely afraid because I was having a child who I was breastfeeding at that time. so, they were somehow unwilling for me to get back to work, but I had to go to work. |
| 1. **Have you noticed any changes in your colleagues’ attitudes in providing MCH services during the pandemic? probe: did you experience a reduction in staff’s work appetite? If yes, explain why (maybe due to lack of risk allowance and patient overcrowding)**   I did not notice any changes. They were just risking their lives and some even got infected and came back to work at the end of their isolation. The continued doing the same work they were doing before.   1. **What incentives were provided by the government to motivate health workers during the pandemic?**   we received some money from the government which help to motivate the health workers. |
| 1. **What is your attitude towards MCH service users during the pandemic? probe: were they making your work easier or more difficult?**   The women coming here were cooperative. |
| **Community factors** |
| 1. **Have you experienced any changes in people’s perception in the community about the use of MCH services during the pandemic? if yes, explain.**   In my community, people were not going to health facilities that much. They used to say that if they go to the health facility, they will be tested for Covid-19 and they would be told that they have Covid-19, especially if you are having a cold. They would say that if you go there coughing and sneezing, they will say that you have Covid-19. For that reason, many people would just go to the Pharmacy, buy medicine and drink that and stay at home. There was that fear all along. Once you start coughing and sneezing, they would say that if I go to the hospital, they will say that I have Covid-19. That why in hospital, we didn’t have much work do at that time because many people do not go to health facilities, but when people come here, we sensitised them, during antenatal visits. We tell them to continue coming for their normal clinics. |
| 1. **Have you experienced any challenges in providing MCH services due to transport difficulties? if yes, explain how**   Transport here was a problem even without the lockdowns and curfews introduced. If you are on afternoon duty, you close very late and at that time, transport is very hectic. If you are on afternoon duty, you close at 8 am but sometimes you have to stay here until 10 pm and normally, when you are living, you change on to your normal clothes. |
| **Institutional factors** |
|  |
| 1. **What do you think of the quality of care provided by this health facility during the pandemic?**   The quality of service was definitely affected by the pandemic because before the pandemic we used to counsel the patients, especially pregnant women, but during the pandemic, we could not do that because you are not allowed to keep them together and not to keep them here for long. So, we just had to do it haphazardly so that they can leave as quickly as possible. |
| 1. **Do you think this health facility had adequate PPEs during the pandemic? if no, give reasons. Did that have any effect on your willingness or ability to provide MCH services?**   We experienced a shortage in gloves. It affected our ability to provide the service because in this ward, you need to use a glove in every procedure you carry out. If you are running here and there just looking for gloves that is another waste of time. |
| 1. **Do you think this facility had enough manpower to provide MCH services during the pandemic? if no, give reasons**   During that time, this place was handicapped because if you get infected you have to quarantine for ten days. Those left on the ground suffered a lot because the workload was too much. That affected our ability to provide the service because if you one person is to do what two people is supposed to do, you will do it but not like how it should have been done if it were done by two people. |
| **Policy factors** |
|  |
| 1. **To prevent infection in health facilities, infection prevention and control measures, such as mandatory screening, wearing of PPEs and face mask, have been introduced in many health centers. What is the effect of these practices on provision of MCH services?**   once you are entering the hospital, you there is a tap at the gate, where people wash their hands before entering the hospital. You are also not allowed to enter here if you don’t have a face mask. Hand sanitizers are also provided at the tap. There measures were strictly observed here. These measures made us feel comfortable because if everyone coming here is having a mask, you feel motivated and protected to provide the service because you feel that even if the person is infected, you feel somehow protected because of the mask. |
| 1. **What is the effect of these measures on utilisation of MCH services during the pandemic?**   Wearing of face mask was always a problem among patients. Sometimes some would put it on until they get into the health facility and put it down once they pass the security guards. They also refuse to wash their hands, especially pregnant women, who have a short temper. If you ask them to wear a face mask, they refuse, so was a big problem for us. Some may also not be willing to the health facility just because of the fact that they have to wear a face mask when they come to the health facility. Those with Asthma it was always a problem for them. Even if they explain their condition to the security guards, they may not understand and may therefore go home and coming back. |
| 1. **Are there any other factors that may have negatively impacted your ability to provide MCH services during the pandemic that I haven’t asked you about? if yes, please state them and explain how?**   for me, the main barrier I had was the fear of contracting the disease and taking it back to my family.   1. **Are there any other factors that may have contributed to the decline in the use of MCH services during the pandemic that I haven’t asked you about? If yes, please state them.**   People were afraid of getting infected. That was the main reason why many people were not coming.   1. **To prevent the decline in use and provision of MCH services in the event of another pandemic or second wave, what do you think the government should do?**   I think the government should use the media to sensitise people. They should tell people everything about the pandemic because still now, there is knowledge lacking regarding the pandemic. The government should also provide us with the necessary materials we need. They should also provide us with incentives and make our working environment very conducive.   1. **What advice would you give to people who are not using MCH services during the pandemic?**   They should continue coming for their MCH services. if you follow the precautionary measures, you will be safe from Covid-19. It is not necessary for you to stay at home doing nothing there. You may have a health problem, and no one will know which can be very risky for you. So, let Covid-19 not stop them from coming for their normal MCH services. |
