# Supplementary material for: Factors that influenced utilization of antenatal and immunization services in two local government areas in The Gambia during COVID-19: An interview-based qualitative study
Source: PLoS One. 2023 Jun 29;18(6):e0276357. doi: 10.1371/journal.pone.0276357 (PMC10309596; doi:10.1371/journal.pone.0276357)
Supplement: S1 File — (ZIP) [file pone.0276357.s001.zip › Supporting information / Health worker 5.docx]

In-depth interview questionnaire for health workers

**Introduction and Consent**

Hello, my name is Abdourahman Bah. I am a final year (MRC sponsored) BSc Global Health student at Queen Mary University of London. I am interviewing health workers and mothers in The Gambia to learn about the impacts of Covid-19-related lockdown measures on utilisation of mother and child services. The interview will take about 30 minutes. All the information I obtain will remain strictly confidential. You may choose not to answer any question that makes you feel uncomfortable.

Do you have any questions?

Do you agree to being interviewed? Yes

| **Background** |
| --- |
| 1. **Could you please tell me where you live?**   I live in Lamin. |
| 1. **Please tell me for how long you have been working in this health facility**?   I was working at North Bank region since my graduation in 2014. I was transferred to this health facility in 2019. |
| 1. **What motivated you into pursuing a public health career?**   When I was at school, I didn’t know what public health is, but used to see one of friends doing these interventions such as immunisation and when I was educated about public health, that was when I build an interest in it. |
| 1. **What MCH services are provided in this facility? Probe: immunisation, antenatal care**   We provide immunisation, antenatal care. These are the two main MCH services that we provide here.   1. **Did the provision of these services continue during the pandemic?**   Yes, although its provision was interrupted at some point, but still service providers were always available. However, during the pandemic, it was somehow difficult because of panic and fear of getting infected, many people run away from using the service. But despite this, the health providers were available to provide the service. We were giving the service to those who were available. |
| 1. **Did the health facility stay open during the pandemic, and for how long?**   There was no point in time when the hospital was closed. As you know, the provision of service must be continued no matter how serious the pandemic is because this is the place people should go if they fall sick in the community. if you close the hospital, you are going to cause more harm. We have teams here that are working tirelessly. So, whenever we have a case here, these teams would go and see the case and isolate the case.   1. **Were there MCH outreach programmes carried out this period?**   No, what we did was to strengthen our promotional and education activities because when people don’t understand a thing, they behave as if they understand, but when we strengthened our health education, this helped us a lot as people started coming back again. We told them to observe the precautionary measures. We also told them having your child immunised is as important as protecting yourself from Covid-19. If Covid-19 causes you to run away from using MCH services, in the long-run, it is going to cause us more harm than benefit. So, when we spread that message to them, we saw turn-out coming up again because they now have understood how to protect themselves when using MCH services. Covid-19 should not prevent you from using MCH services because we are preventing disease. For example, measles outbreak was occurring for some time back in the days. Now it is not happening because of this immunisation. If Covid-19 comes up now and prevented people from using their measles vaccines, in the long-run, we may have another outbreak again. So, when we spread that information through health education activities, it helped us. So, we got back to our normal coverage. The service interruption was about less than a month. It was at the beginning of the rise of Covid-19 cases, but since then, we started our health education interventions and from then things started to improve. |
| 1. **Have you noticed any changes in utilisation of MCH services during the pandemic? For example, do you see fewer or more patients than usual?**   We obviously a noticed a change in the utilisation of MCH services because here we normally go for an in-service for every three months. That is, every three months, we conduct a review to see our performance during those months. We check whether we have met our target or not. So, during that discussion, we were able to notice that the service that we provided during the pandemic has dropped compared with the beginning of the pandemic. So that along is evidence to show that the service we provided during the pandemic was affected. The factors that could have contributed this decline could have been fear because everyone knows that Covid-19 is a new thing. So, we don’t know much about it. The media is also talking about it everywhere. There was also much conspiracy going out there. So, based on that I can say it was all about panic. People wear afraid of contracting the disease. So, they ran away from health facilities. When some of the health workers here were positive, people ran way, including those who were sick and came to seek a cure. They forgot about their sickness and ran away. |
|  |
| **Individual factors** |
| 1. **From the perspective of health workers, how safe do you think it is to provide MCH services during the pandemic?**   You know then it was a problem really. Though health workers were trained to some level to know the basic precautions, you have to follow those basic precautions to protect yourself and others around you. These include wearing a face mask when you are attending to a patient, continuing with hand hygiene and maintaining some distance between you and others. Those procedures were implemented. |
| 1. **How safe is for women to access MCH services in this facility at that period?**   We have a regulation here. That is, during the Covid-19 pandemic, we did not allow people to access MCH services without being in a protective mask. you had to put on a face mask, before you can access MCH services here. We went even to the extent that we sent you out if you don’t put on a face mask. You will not access our service unless you go and get a face mask. So that, you can protect yourself and others around you because we understand that sometimes when you tell them to observe this social distancing it becomes a problem, but in that case, when you are in a face mask, you can at least get some protection. |
| 1. **Did you or your colleagues work more or less hours during the lockdown? If yes, please explain why?**   When you say a pandemic, it means an emergency situation. Nothing is as usual. So, it depends on how cases come, even if you are to close at 4 pm and you happen to have an emergency, you have to work overtime. So, we worked more time than normal. During an emergency, you have to work longer than normal because even if you have to close at 4 pm, there will be people at stand by in case you are called for case some where else. You have to go and do an intervention to make sure that the suspected cases are investigated and taken to the isolation area. So, meaning you have to work extra time de as work may not be as usual. |
| **Interpersonal factors** |
|  |
| 1. **Have you noticed any changes in your colleagues’ attitudes in providing MCH services during the pandemic? probe: did you experience a reduction in staff’s work appetite? If yes, explain why (maybe due to lack of risk allowance and patient overcrowding)**   I don’t know about other departments, but in our department, there was none like that as we have a duty roster. So, whoever is on duty, you know that you are supposed to be at work. Whether in the pandemic or not, you have to be at work. It is your responsibility to be at work during your duty roster. There is nothing like I don’t like to go to work because of so and so forth. So when you are there, you have to provide the service.   1. **What incentives were provided by the government to motivate health workers during the pandemic?**   We don’t want to talk much about that area because there was a time when they said health workers were going to receive some incentive because of their role in the pandemic. I believed some people received these incentives, but I cannot say that was the case every health worker. The majority of health workers received some incentives |
| 1. **What is your attitude towards MCH service users during the pandemic? probe: were they making your work easier or more difficult?**   You know anytime something new comes, you to talk to people and you have health educate them. So, it was somehow tough at that time before they can understand things, but later as we strengthen our health education and promotion activities, it made our lives easier because it made people understand why we are saying do this, such as put on a face mask and maintain hand hygiene. So, when they understood, they made our lives easier. They themselves sometimes when they see you as health provider not following the precautionary measures, they tend to remind you to put on your face mask. In that case, it means they have understood. |
| **Community factors** |
| 1. **Have you experienced any changes in people’s perception in the community about the use of MCH services during the pandemic? if yes, explain.**   Yes, during the pandemic, we normally provide HPV campaign to schoolgirls to prevent cervical cancer. So, our first round was okay, but because of the pandemic, when we were doing our second round, we encountered a problem. We were unable to meet a target because people didn’t understand. They thought that the vaccine we were giving was about Covid-19. We strengthen our health education campaign until we were able to overcome that rumour. |
| 1. **Have you experienced any challenges in providing MCH services due to transport difficulties? if yes, explain how**   Yes, preparedness was lacking a bit. At the beginning, I can take an example of contact tracers, like myself, we need to be mobile to be able to conduct our contact tracing. So, if we are not mobile, it will be difficult for you to go and conduct your contact tracing. At the beginning, not even everyone was mobile, like team leaders were having their motorcycles but the subordinates of the team were not having motorcycles. But at the end, we received motorcycles from other private organisations such as UNICEF. However, there are still some who don’t have motorcycles. So, meaning for them transport is a constraint still. |
| **Institutional factors** |
|  |
| 1. **What do you think of the quality of care provided by this health facility during the pandemic?**   As I said the service was affected but those does not prevent service providers from rendering their service. It should be a two way around. That is, it should a give and take. If I am supposed to give a service, there should be someone to receive the service. If those people are not around, it will have impact on the service. Service providers were there and ready to provide the service. There was no time here when we said immunisation services are no longer being offered. We changed our modality because we had to observe how this disease spread. If you want to share everything through weighting. If you are to weigh a child, they all share the same weighing machine and for some you have to undress them for them to be weighed. So, they are all using the same surfaces. We all know the mode of transmission. Your hand can transmit the disease from touching your mouth and surfaces. So, in that case, we changed our modality. That is, we stopped weighing because we don’t want to be facilitating the spread of infection to others through our weighing process. Wherein in immunisation, you come and sit along, and we will inject your child with a syringe. This syringe is auto disable. We will discard it when you are done with the immunisation. So, there was no point in time when you will say that we stopped our immunisation service at this facility. |
| 1. **Do you think this health facility had adequate medical supplies during the pandemic? if no, give reasons.**   What we normally use in immunisation is usually the vaccines, syringes and safety boxes. In our service, we are not encouraged to use gloves that much. So, during the pandemic, we tended to focus more on hand hygiene. When conducting immunisation, you cannot be going out every time to wash your hands after immunising a child. What we normally do is to provide hand sanitisers. So, we can use this hand sanitisers which when you apply properly can take you up to six hours. You don’t have any immunisation that can take you for more than six hours. So, if you apply a hand sanitiser properly, this can serve you for a long period of time.   1. **Do you think this health facility had adequate PPEs during the pandemic? if no, give reasons. Did that have any effect on your willingness or ability to provide MCH services?**   There was a time when we experienced a shortage of PPEs, especially face masks. If we ask people to put on a face mask and as a service provider, you cannot be seen without a face mask. That is, you cannot be telling people to do this when you are not doing it yourself. That will one way or the other will affect your ability to provide the service. |
| 1. **Do you think this facility had enough manpower to provide MCH services during the pandemic? if no, give reasons**   There was a human resource constraint during the pandemic because other staff were relocated. So, in that case, there was a heavy workload on others on the ground here.   1. **What do you think of the health facility environment? Probe: is the facility clean and not overcrowded?**   Really, it was not conducive because not everybody knows how to follow the precautionary measures. So, in that case, every health worker is at risk of getting infected. Some people didn’t even know how to apply the measures to protect others from getting infected. So, in that case, you are the health worker provider, you have to protect yourself from getting infected, but others might not how to do that. So, that was a big constraint at that time. |
| **Policy factors** |
| 1. **What is the effect of these measures on utilisation of MCH services during the pandemic?**   You at the beginning, let me say, human beings are always like that. When they are used to doing something normal, if anything comes and alter that situation. At the beginning, there must be some degree of rejection, but it will be left to you the service provider to provide some effort to combat that degree of rejection. So, when we strengthen our health education promotion, they understood why we are imposing on them to put on a face mask. It is not because we are trying to segregate you or because you have Covid-19. It is for your own good. So, after understanding all these, if now they see you as a health provider not putting on a face mask, they will ask you why you are not wearing a face mask. So, in that case, everyone will obviously put it on. However, it is worth noting that some may not have been coming because they may have some breathing problems since everyone who comes here must put on a face mask, but in this health facility during the clinic service, women will be coming with their face mask and at some point, in time, they will be lowering their face mask. So, if you want to have that encounter with them, they will tell you “I am an Asthmatic patient, so if I put on for a long period, it will affect my breathing. So, in that case, we understand their condition, but when you lower it for a while, we will ask you to put it back. If there is somebody who is sick, we give more attention to that person. So, we will provide service to you as soon as possible so that he/she can leave. |
| 1. **Are there any other factors that may have contributed to the decline in the use of MCH services during the pandemic that I haven’t asked you about? If yes, please state them.**   For Women who are working, it is sometimes a problem. I can say the male involvement. That is, mostly when you involve males in accessing MCH services will be beneficial as some women are workers. So, for them to bring their child can be difficult for them because they have to report to work at 8 am. So, if you are to report at 8 am and you are supposed to bring your child to the health facility, in one or the other, it becomes a problem. So, for most of those women, in some health facilities, they will provide a specific day for them, like during weekends, but for us here, we don’t work on weekends. So, such cases, if men are not involved to help in such situations, that could be contributing factor to the decline in the uptake of the service. So, male involvement is very important.   1. **To prevent the decline in use and provision of MCH services in the event of another pandemic or second wave, what do you think the government should do?**   My advice the government now as what to do in this situation is to stay intact and alert people on everything that is happening. If there is a surge in cases, the government should alert people as soon as possible so that people will know what is happening. The government should also try if the health personnel is providing recommendation, the government should be the one to enforce those interventions so that they can minimise the spread of the infection. Also, supplies must be available. That is, government should try and make sure that supplies are available for health providers for them to be able to provide the service. If there is a shortage of supplies, the service will be affected drastically.   1. **What advice would you give to people who are not using MCH services during the pandemic?**   I think that issue has been resolved because at the beginning when this pandemic started that was when we had defaulters, but after that activity, the service uptake increased again. It is just recently that conducted a defaulter tracing activity. That activity was purposely geared towards those who ran away from using the service at that time. That activity concluded two weeks ago. We conducted that activity to catch up with those people. That is another intervention that we normally conduct in order to catch up those defaulters. |
