# Supplementary material for: Factors that influenced utilization of antenatal and immunization services in two local government areas in The Gambia during COVID-19: An interview-based qualitative study
Source: PLoS One. 2023 Jun 29;18(6):e0276357. doi: 10.1371/journal.pone.0276357 (PMC10309596; doi:10.1371/journal.pone.0276357)
Supplement: S1 File — (ZIP) [file pone.0276357.s001.zip › Supporting information /Respondent 6.docx]

In-depth Interview Questionnaire for MCH service Users

**Introduction and Consent**

Hello, my name is Abdourahman Bah. I am a final year (MRC sponsored) BSc Global Health student at Queen Mary University of London. I am interviewing health workers and mothers in The Gambia to learn about the impacts of Covid-19-related lockdown measures on utilisation of mother and child services. The interview will take about 30 minutes. All the information I obtain will remain strictly confidential. You may choose not to answer any question that makes you feel uncomfortable.

Do you have any questions?

Do you agree to being interviewed? Yes

| **Background** |
| --- |
| 1. **What is your ethnicity?**   I am a Jola   1. **Could you please tell me where you live – Probe: house of residence is?**   I live in Tallinding. I brought my child for immunisation |
| 1. **Please tell me how you got here today? Probe: public transport, private or walked.**   I used public transport |
| 1. **Have you used MCH services during the pandemic? if yes, what MCH service have you used during the pandemic?**   Actually, I went to one health centre for a workshop on behalf of my counsellor. Actually, I was not taking my child for immunisation during the pandemic. I have not been bringing my child for immunisation for about six months now. I was told to bring by child for immunisation, but I travelled. So, I decided to bring her today for the vaccination. I was also not coming because of the Covid-19 pandemic due to public gathering. I was afraid of coming here and getting infected. During that time, people were afraid of coming to health facilities even if you are sick, you would decide to go to the pharmacy instead of going to the hospital because of the pandemic. I was very scared of this pandemic. For me, since the pandemic started, I did not bring my child for immunisation at all. For me, personally, I was following the protocols because at that time, I would not go anywhere without putting on a face mask. I also wash my hands regularly, but I still felt uncomfortable to come to the health facility because I knew that social distancing cannot be maintained in the hospital. |
| 1. **Have you changed the way you access this service during the outbreak? If so, how? If you have changed, are you going more times or less times and if so, what are the reasons? Probe-economic? Fears?**   When I re-started bringing my child for immunisation, they did not complain. When I came and gave them the immunisation card, they decided to give my child vitamin A. I then looked at the card, I noticed that my child was supposed to be vaccinated, but they did not vaccinate here. I raised this point to the nurse, that was when he vaccinated her for measles. I should now come for the other vaccine next month. |
|  |
| **Individual factors** |
| 1. **How safe do you think it is to access MCH services during the pandemic? - Probe: have these concerns stopped you from using these health facilities?**   I think right now it is safe as things are getting back to normal now. Even when you come to the gate, they would send you back if you don’t have a face mask. I experienced one thing at Kanifing General Hospital, where my sister has been admitted for a week, I went outside the hospital to buy breakfast for her, but I left my face mask inside the hospital, I was asked to get a mask in order to get back inside. So, I believe that they are now enforcing the rules more than they were doing before. |
|  |
| **Interpersonal factors** |
| **18.What is your family’s attitude, including your husband, in your use of MCH services during the pandemic? Probe: Do they encourage or discourage you? In what way?**  My family was supportive, especially my husband. He would always give me permission to take my child to the health facility. He never told me not to take my child for immunisation to the health facility because of safety reasons. |
|  |
| **Community factors** |
| **20.Have you noticed any changes in people’s perception in your community about the use of MCH services during the pandemic? if yes, explain. Probe: give examples of people being afraid of visiting facilities due to stigma associated with visiting health facilities or fear of being quarantined etc.**  Yes, the problem is that people don’t believe in the existence of the Covid-19 pandemic because for me, I went for a workshop with my counsellor in New Jeshwang health centre. I was told that when I come back, I should share what I have learnt with other people. So, when I came back, I tried to sensitise people in my community about the pandemic, but they were not even interested. Everybody was telling me that, they would not take their children for immunisation because their children will be given the Covid-19 vaccine which is not safe. So, I stopped there and did not try anymore. This is because of ignorance; I live in a society where the majority of people are not educated. I have not seen anyone being stigmatised because of the fact that they went to a health facility. The only stigma that I see is that when you cough or sneeze in a public, people would usually try to stay away from you. |
| **21.Has this had any impact on your use of MCH services during the pandemic? if yes, explain how**  Not actually because for me, they asked me to not come for immunisation for six months because of the Covid-19 pandemic. I used to come every month, but I had to stop because the health workers told me not come for six months, that is until my child is one year and nice months old. |
| **Institutional factors** |
|  |
| **24.Are you satisfied with the care provided by this health facility during the pandemic? probe: consultation time, treatment and respect from health workers. Has this stopped you from visiting health facilities?**  In this health facility, they have now stopped weighing children. They only ask you to bring your child when they need to be immunised. They stopped the weighing, but I don’t know why that is the case. They used to weigh children before the pandemic, but they have stopped it now. I think it might be to reduce the risk of infection. I think the pandemic had some beneficial impacts in terms of improved hygiene because they introduced a washing station at the gate, where people wash their hands before entering the hospital and put on a face mask. The main issue was, however, social distancing was never observed in this health facility. Maintaining social distancing was a problem, which I believe is because of lack of resources because there were not enough chairs and not much space. |
| **25.Do you think this health facility had adequate medical supplies during the pandemic? if no, give reasons. Probe- has this stopped from visiting health facilities.**  When my child was sick, I would sometimes have the medicines here, but at times, I would have to buy them at the pharmacy. |
| **27.What are your perceptions about the health workers in this facility? (e.g., competence or behaviour of health workers). probe- has this stopped you from visiting health facilities.**  you see people come from different places. At times, when you come, these female nurses would sometimes lose their temper and say unpleasant things to people. Nursing, however, requires patience. If you see someone doing something wrong because of ignorance, you should take the right steps to help the person understand what they have done wrong. You should not force people to do something that they don’t want to do. This is a problem for female nurses. At times, when you don’t know if your child needs to be vaccinated and when you ask them to check it for you, they will sometimes lose their temper and tell that they are busy. I might be one of the reasons why some people were not bringing their children for immunisation because for me, personally, if you embarrass me Infront of people, I will stop coming to the hospital. Also, even at times you would see us fighting with them because they keep embarrassing us Infront of people. Sometimes, people even choose to go to the big pharmacies or private hospitals, where they will not be treated badly by the health workers. Last time, I met a lady here, she only brought her child for immunisation when she was just three months and did not bring her again until the child was over a year old. She said she travelled. On that day, she met a male nurse here and he told her that he would give the child all the injection that she missed. The mother started crying and saying that her child would die because of that. If it were a female nurse, she would have made it very difficult for that lady. |
| **28.Do you think the health workers were following the Covid-19 precautionary measures appropriately? For example, were they always wearing face mask and PPEs? Probe-has this stopped from visiting health facilities?**  Some of them were following the Covid-19 precautionary measures correctly and some of them, when you come here, you would see that they were not wearing a following the rules, such as not wearing a face mask. Even social distancing, they were not observing it that much. |
| **Policy factors** |
|  |
| **30.To prevent infection in health facilities, infection prevention and control measures, such as mandatory screening, wearing of facemask and social distancing, have been introduced in many health centers. What is the effect of these measures on your use of MCH services during the pandemic?** |
| For me, I know that these are the protocols, so I have to follow them. For me, that is my life’s motto, I give the devil its due.  **32. What do you think is the effect of these measures on other people’s willingness to come for MCH services?**  I know this can, however, be a problem for others, such as those with Asthma. I know if you are an Asthmatic patient, you will have issues with breathing when you put on a face mask. When we, the nursery schoolteachers, went for a workshop, they asked us to declare any health issues that we had. All those with Asthma were asked to sit by the window so that that they can have proper ventilation. But here, if you have Asthma and explain that to the health workers, especially female nurses, they will not understand your situation. So, they may decide to stop coming to the health facility and go the pharmacy, where most of them do not provide immunisation services. The private health facilities are also very expensive, which most people cannot afford. So, they would just sit at home. |
| **35. What do you think the government should do to prevent a decline in use of MCH services in the event of another pandemic?**  The government should continue with their sensitisation efforts and engage with local leaders to help sensitise the masses. Here the main problem is lack of sensitisation, but if the engage the local leaders and use public places such as markets, schools and Bantabas, to sensitise people, this will help motivate people to come to health facilities. They should engage with community leaders because they are ones working withing the communities. They should also organise workshops to sensitise people even it is just three days workshops. Every health centre has a CEO, so the government should work with those leaders to help change the behaviour of some health workers. |
