# Supplementary material for: Factors that influenced utilization of antenatal and immunization services in two local government areas in The Gambia during COVID-19: An interview-based qualitative study
Source: PLoS One. 2023 Jun 29;18(6):e0276357. doi: 10.1371/journal.pone.0276357 (PMC10309596; doi:10.1371/journal.pone.0276357)
Supplement: S1 File — (ZIP) [file pone.0276357.s001.zip › Supporting information /Health worker 4.docx]

In-depth interview questionnaire for health workers

**Introduction and Consent**

Hello, my name is Abdourahman Bah. I am a final year (MRC sponsored) BSc Global Health student at Queen Mary University of London. I am interviewing health workers and mothers in The Gambia to learn about the impacts of Covid-19-related lockdown measures on utilisation of mother and child services. The interview will take about 30 minutes. All the information I obtain will remain strictly confidential. You may choose not to answer any question that makes you feel uncomfortable.

Do you have any questions?

Do you agree to being interviewed? Yes

| **Background** |
| --- |
| 1. **Could you please tell me where you live?**   I am from Bundung. |
| 1. **Please tell me for how long you have been working in this health facility**.   I have been working here since 2015. |
| 1. **What motivated you into pursuing a public health career?**   Public health pertains to preventing conditions that affect the population. |
| 1. **What MCH services are provided in this facility? Probe: immunisation, antenatal care**   Basically, this health facility is concentrated on providing maternal and child health services. we provide antenatal care services, immunisations, and the like. We even have a paediatric ward here, where deliveries are conducted. It is so far one of the best when it comes to maternal and child health service in the Gambia. The structures available here and the personnel are also very hardworking.   1. **Did the provision of these services continue during the pandemic?**   Yes, all these services here being provided. We just adjusted some services to make sure there is some distance maintained between people. Before the pandemic, we used to weigh children here, before being immunised inside. Also, health education activities were also going on at that time. During the peak of Covid-19, the weighing was suspended. Up to now, we have not resumed weighing. This is just to make sure that we adhere to the protocols of infection control due to Covid-19. Also, before the pandemic, our office used to be filled with people to be immunised. We used to have about ten to fifteen inside the office to be immunised, but during the Covid-19 pandemic we reduced it to five people. This was done to maintain a social distance of at least one metre. Also, before the pandemic, pregnant women used to come every day at the antenatal area, but they also put in strategies. I am not sure if the strategy I saw them introduce is part of their social distancing measures, but I saw during the pandemic that they were giving appointments only on the first every month and during all the other days, you only come to bring your paper. So, that long queue that they used to have before the pandemic was reduced. So, I think that is part of it because that measure came around that time. |
| 1. **Did the health facility stay open during the pandemic, and for how long?**   This health facility was never closed, but we had situations here when the majority of staff tested positive. So, work was slowed down for a bit because most of them were taken to the hotel and some people were stretching, but it was never closed. There were some restrictions for the number of people visiting.   1. **Were there alternative strategies for immunisation activities**?   No, since the inception of this health facility we have introduced outreached programmes. Along the line, there were some discussions to conduct outreach immunisation activities here, but it has still not been possible due to certain issues. Actually, all the people coming here are not all from Bundung. Bundung per see, our catchment area is not that wide, but because of the quality of services here people are coming here from different areas. For example, most people who delivered here tend to continue here and other people who delivered elsewhere trust our service very well. |
| 1. **Have you noticed any changes in utilisation of MCH services during the pandemic? For example, do you see fewer or more patients than usual?**   We experienced a big changed in the number of people coming for MCH services at that time because there was a scenario when the first case positive case was detected here and when people’s awareness of Covid-19 was not great. So, along the line a rumour emerged that this person is positive, and people started running here. That is, people who came for the service started running. It was very serious. It was like Covid-19 is something that would go after people. So, people started running. Even when I went home, my neighbours started asking me if we have a positive case at the health facility. So that affected our service. We had to continue health educating them, but really affected our services. some also started to confuse the vaccines we give here with Covid-19 vaccines. some husbands were telling their wives do not take our childhood vaccines. As a result, the number was very reduced, but along the line we also introduce some measures. As of now, the service coverage is now back to normal. |
|  |
| **Individual factors** |
| 1. **From the perspective of health workers, how safe do you think it is to provide MCH services during the pandemic?**   Health providers do risk a lot. It was very risky, but we are trained to serve our people. No matter how rough the situation is, we cannot run away. we as health workers, if we run away and stay at home, that would be the collapse of the health system of the Gambia. Public health officers, nurses and doctors cannot stay away from work. They just have to take the necessary precautionary measures but even the precautionary as we know in The Gambia, our PPEs are not up to standard, and they are not adequate. |
| 1. **How safe is for women to access MCH services in this facility at that period?**   It was not safe for them too, but it was in such a way that if we do not motivate them or they do not come, it can lead to the collapse of our immunisation system in the Gambia because The Gambia has one of the best immunisation rates in Africa, but the figures were falling at some level as people were scared. So, we had to talk to them and when they come, we asked them to observe social distancing. This was why we even stopped weighing so as to encourage them. When weighing, they will be closed to each other. They will spend a longer time here. So, for that reason we stopped weighing, though weighing is very important as we monitor the growth of the child, and the immunisation is also important as it prevents other infectious diseases. So, we had to balance it to make sure that the immunisation is continued to prevent other diseases. so that is the most important one. So, we decided to stop the weighing instead of the immunisation. When they knew there was no weighing, and they would just come and go and if they don’t need to be immunised, we would not keep them here. The length of time of health education was also reduced so that would not keep them here for long. So, when they know that the time, they are going to spend here is less, they tend to come as they want to stay here for long. In that, we gave them the courage to come as want they want is to come and leave early. So, when we assured them than, though our main aim is to protect. Leaving here early is advantageous to them as they can do other chose at home. So, that is how we managed. Hadn’t we stopped the weighing; it would have been very difficult for us. Some are afraid of getting infected, so that is why they didn’t come, but for others it was because of the long time they spend here, |
| 1. **Did you or your colleagues work more or less hours during the lockdown? If yes, please explain why?**   For some of the staff here, there was a cut down of time. Some will come at this time and others will come another time. This was done to make sure that the contact hours staff have here is not much. Some of them also went the extra mile, like I was coming here during weekends to vaccinate children. Normally when women deliver during weekends, they will have to wait for us until Monday when we can vaccinate their children, and when we vaccinate their children, we ask them to come back in three days, so that the nurses will check them again. What the nurses will tell them is to come back if the child has any problems, but not to come a queue after three days. When I immunise them on Saturday, they can then go home. They don’t have to stay here on Sunday. So, I was doing that during the weekends during the Covid-19 pandemic to make sure that we release them early. So, for me, I was working more time during the pandemic. I was doing that because I am a native of Bundung. All the others are from a far, such Brikama. I am also having a motor bike, so even if I don’t use the bike, I can walk from my home to here. I made that sacrifice to help my colleagues. |
| **Interpersonal factors** |
|  |
| 1. **Have you noticed any changes in your colleagues’ attitudes in providing MCH services during the pandemic? probe: did you experience a reduction in staff’s work appetite? If yes, explain why (maybe due to lack of risk allowance and patient overcrowding)**   The departments differ as some far have less contact with patients. For example, in our department, we only come in contact with patients during immunisations and most of the patients that we see are pregnant women and children. Children may not be expose that much as they don’t go out often. So, for us, we have more courage to see the patients as we are seen a newborn child. There was this media for people to sit at home. As such, people were always keeping their kids at home. We will not be that scared because of the kind of people we are dealing with. We deal with very few pregnant women, just like the one that just came here. Those who deal with other patients such the OPD staff, it is very risky. So, I don’t know if there was anyone here who refused to come to work during the pandemic, but I have heard that in other health facilities, some health workers tend to stay at home, but here I have not heard of that, like in other places, there was that fright among health workers.  Even us, who don’t get in much contact with sick people, were very careful. Among us, no one sat at home. In this hospital in general, I have not heard of anyone deliberately sitting at home apart from the days off some were given to rotate with others so as to reduce the chances of infection because some staff were infected at that time.   1. **What incentives were provided by the government to motivate health workers during the pandemic?**   We received some incentives. I think everyone was given five thousand dalasi each to motivate us. Also, during our capacity building trainings, we received some incentives. Aside from that, the government also gave us some money. Those who were also part of the defaulter training received some money as well. I wasn’t part of that team, as I am part of the rapid response team of this hospital. I was the one who took the first badge of positive cases to the hotel. It was around 12 midnight when I took them to the hotel, but I was not part of the regional rapid response team. Those who were in this team each was given about seven thousand dalasi every month or every three months. They were selected from here, but not everybody can be part of that response team because some people need to be here. |
|  |
| **Community factors** |
| 1. **Have you experienced any changes in people’s perception in the community about the use of MCH services during the pandemic? if yes, explain.**   Yes, there was change in people’s willingness to visit health facility. Some were even confronting us. Some were thinking that Covid-19 is not real. They were saying that it was not safe to come this health facility as they heard some people here had gotten infected with Covid-19. There were saying all sorts of things. As a result, many people sat home and refused to come to the health facility. We later had a defaulter tracing exercise. We were able to identify those who were not bringing their children for immunisation by checking the records in our electronic database. We went to their homes several times. When we went there, they were telling us, “I thought it was not safe to come to your health facility”. We immunised their children at their homes and health educated them. I was born in Bundung here, so many people know me. For that reason, they tend to listen to me when I talk to them as they trust me. |
|  |
| **Institutional factors** |
| 1. **What do you think of the quality of care provided by this health facility during the pandemic**?   Apart from the weighing, which we stopped during the pandemic, all the other services were delivered as expected. The quality was still maintained |
| 1. **Do you think this health facility had adequate PPEs during the pandemic? if no, give reasons. Did that have any effect on your willingness or ability to provide MCH services?**   During the pandemic, we were having some supplies of PPEs, but I cannot say it was adequate. We used to have supplies in our store, but because of the way it was used, it ran out a bit. For those in our department, we just need mask, we don’t need that sophisticated PPEs. It is the midwives who need those sophisticated PPEs, for us we only need a mask. |
| 1. **Do you think this facility had enough manpower to provide MCH services during the pandemic? if no, give reasons**   In our department, nobody was taken for quarantine in the hotel. It was only during the second test that some tested positive, but even before the results came out, they had already recovered because Vitamin C was given to people and the like. |
| **Policy factors** |
| 1. **What is the effect of these measures on utilisation of MCH services during the pandemic?**   We had some challenges with some people for wearing face mask, but at the end, they complied because we used to tell them without a face mask, you will not entre the health facility. Some used to escape from the gate and come up to here, and we would tell them to go and collect a face mask. Then, they would go and collect a face mask, but there were some who would insist on not wearing a face mask, but at the end of the day, they end up wearing it. We used to have some people said they cannot wear a face mask because they have Asthma, so we accepted that since that is part of the laws. Depending on the person they meet at the gate, if they understand their condition, they let them in, but they don’t understand, they will be sent way, when they get us, the health workers, we are supposed to understand them. So, for us that may not be a problem, but at the gate, if the person is someone who gets angry easily because of the words from the security guard, she may go home and not come back. |
| 1. **Are there any other factors that may have negatively impacted your ability to provide MCH services during the pandemic that I haven’t asked you about? if yes, please state them and explain how?**   For me, I have worked for almost eight years. I was in the provinces working in a health facility. I was providing outreach services about ten to fifteen kilometres away from the health facility. So, it is much easier here as you sit at one place. Despite all that pressure and the rain in the provinces, I was able to cope. So, for me it will not be difficult to work here.   1. **Are there any other factors that may have contributed to the decline in the use of MCH services during the pandemic that I haven’t asked you about? If yes, please state them.**   Media issues are disturbing us and vaccine hesitancy. People tend to write information anyhow they feel. Some people believe the media more than the health workers. That was affecting us, especially the WhatsApp groups, where they keep telling people not to send their children to health facilities and don’t to get them immunised. Even here, some would bring their children, but the moment they know we are going to immunise them, they refuse. They would tell us, “My husband told me not to vaccinate my child. So, we had to talk to their husbands on the phone. We tell them that these vaccines have nothing to do with Covid-19, as this has been going on since before Covid-19 came around. We also tell them, not having the vaccines is going to affect the child. There were also some factors that were affecting some women as they were not coming for immunisation even before the pandemic, but this is all because of media influence. The media was playing a negative role in immunisations. What saved us was the fact that the health workers and the presidents took the Covid-19 vaccine and there was no incident. That was when people re-started trusting our childhood vaccines.   1. **To prevent the decline in use and provision of MCH services in the event of another pandemic or second wave, what do you think the government should do?**   The government should learn from our past experience. The government should encourage the minister of health to continue with the sensitisation efforts. The government should also continue with the encouragements given to health workers to make sure that they are motivated. The health message should also continue to follow because the pandemic is now not something that is totally new to people, as people now have an idea about Covid-19. We are now learning to live with it. The measures that we took to make sure that it was reduced, we should just continue with those measures. The government should make sure that the health sector does not relax the efforts they were doing. |
