# Supplementary material for: Factors that influenced utilization of antenatal and immunization services in two local government areas in The Gambia during COVID-19: An interview-based qualitative study
Source: PLoS One. 2023 Jun 29;18(6):e0276357. doi: 10.1371/journal.pone.0276357 (PMC10309596; doi:10.1371/journal.pone.0276357)
Supplement: S1 File — (ZIP) [file pone.0276357.s001.zip › Supporting information /Respondent 7.docx]

In-depth Interview Questionnaire for MCH service Users

**Introduction and Consent**

Hello, my name is Abdourahman Bah. I am a final year (MRC sponsored) BSc Global Health student at Queen Mary University of London. I am interviewing health workers and mothers in The Gambia to learn about the impacts of Covid-19-related lockdown measures on utilisation of mother and child services. The interview will take about 30 minutes. All the information I obtain will remain strictly confidential. You may choose not to answer any question that makes you feel uncomfortable.

Do you have any questions?

Do you agree to being interviewed? Yes

| **Background** |
| --- |
| 1. **Could you please tell me where you live – Probe: house of residence is?**   I am from Farato, but I was staying here. I have not yet transferred to another nearby health facility. |
| 1. **Please tell me how you got here today? Probe: public transport, private or walked.**   I got here by using public transport. |
| 1. **Have you used MCH services during the pandemic? if yes, what MCH service have you used during the pandemic?**   During the start of the pandemic, I was pregnant at that time. I used to come for antenatal care every month. I did not miss a single month during the pandemic. I attended all of my appointment. |
|  |
|  |
| **Individual factors** |
| 1. **How safe do you think it is to access MCH services during the pandemic? - Probe: have these concerns stopped you from using these health facilities?**   In my opinion, it was safe because if you follow the Covid-19 precautionary measures, you will be safe from getting infected. Others don’t believe that Covid-19 is real, but we are now hearing that there are many positive cases of Covid-19 in The Gambia, so we can no longer say it is not real. However, I have never seen a positive case with my own eyes, but I keep hearing about it in the news and from people as well. |
|  |
| **Interpersonal factors** |
| **18.What is your family’s attitude, including your husband, in your use of MCH services during the pandemic? Probe: Do they encourage or discourage you? In what way?**  My family was supportive, especially my husband. My wellbeing has always been his main priority. He would make sure that I come for antenatal service every month and that, I follow the precautionary measures correctly, so that I can protect myself from getting infected when I come to the health facility. |
|  |
| **Community factors** |
| **20.Have you noticed any changes in people’s perception in your community about the use of MCH services during the pandemic? if yes, explain. Probe: give examples of people being afraid of visiting facilities due to stigma associated with visiting health facilities or fear of being quarantined etc.**  I used to hear people saying that many people are not coming to health facilities during the pandemic, but I haven’t met anyone in my community who is supposed to come for MCH services but stopped because of the pandemic. I used to even come with other people from my community during the pandemic. |
|  |
| **22.Have you experienced any challenges on getting to health facilities during the pandemic? if yes, state them (e.g., lack of transport)**  Yes, I used to experience transport difficulties because there was shortage of vehicles at that time. So, I used to arrive late sometimes because of transport issues. I would leave home around 6 am and get here around 9 am. This is because lack of enough vehicles during the pandemic. This was because of the pandemic as many drivers stopped working because of social distancing measures that they had observe in their vehicles. Despite this difficulty, I continued to come for antenatal service every month. |
| **Institutional factors** |
| **23.Did the health facilities stay open during the pandemic? if no, state how this may have affected your access to MCH services.**  Some of the health facilities that I went to during the pandemic was closed because of the pandemic. So, I decided to come this health facility, which has never been closed since I started coming here. |
| **26.Do you think this facility had enough manpower to provide MCH services during the pandemic? if no, give reasons**  We used to stay here for a long time before we could access the service but that was not because there not enough health workers, it was because there were many people coming here at that time since other health facilities were closed or reduced the number of patients that they take during the pandemic. So, if you want to leave here early, you had to come very early. They attend to people according to who arrived first. They would also ask us to observe social distancing. We used to sit in fours but during the pandemic, we had to sit in threes and ensuring that we leave a safe distance between one another. |
|  |
|  |
| **Policy factors** |
|  |
| **30.To prevent infection in health facilities, infection prevention and control measures, such as mandatory screening, wearing of facemask and social distancing, have been introduced in many health centers. What do you think of the implementation of these measures in the health facilities? Probe: were they implemented correctly?**  When we come here, they would ask us to put on a face mask, observe social distancing and regularly wash our hands. We had to do all that whenever we came here during the pandemic. The health workers also had to wear face mask when attending to patients. This was all done to protect us from getting infected. |
| **31.What is the effect of these measures on your use of MCH services during the pandemic?**  These measures did not prevent me from coming for antenatal services during the pandemic because I knew that these measures are there to protect me and others from getting infected. I did not have any problem with wearing face mask.  **32. What do you think is the effect of these measures on other people’s willingness to come for MCH services?**  I know that these measures may prevent some people from coming MCH services in the health facilities because people have different lifestyles and may feel uncomfortable to wear face mask. |
|  |
| **35. What do you think the government should do to prevent a decline in use of MCH services in the event of another pandemic?**  The government has tried a lot during the pandemic. They have provided different forms of support. For example, they have provided hand washing stations in health facilities and other public places all over the country. So, they should continue doing the same things. They should also sensitise people about the pandemic so that people will understand what the Covid-19 is and how to protect themselves. If this is done, I believe people will feel safe to come to health facilities.  **36. What advice would you give to people who are not using MCH services during the pandemic?**  If you sit at home and refuse to come for antenatal service or stop bringing your child for immunisation, you will be putting yours and your child’s wellbeing at increased risk because even if you have a headache, you will not know the cause of the headache. If your child gets sick, you will not be able to identify the cause of the illness. So, you have to come to the health facility, where they will carry out some test to understand what is wrong with you or your child and give you the necessary medical attention you need. |
