# Supplementary material for: Factors that influenced utilization of antenatal and immunization services in two local government areas in The Gambia during COVID-19: An interview-based qualitative study
Source: PLoS One. 2023 Jun 29;18(6):e0276357. doi: 10.1371/journal.pone.0276357 (PMC10309596; doi:10.1371/journal.pone.0276357)
Supplement: S1 File — (ZIP) [file pone.0276357.s001.zip › Supporting information /Health worker 9.docx]

In-depth interview questionnaire for health workers

**Introduction and Consent**

Hello, my name is Abdourahman Bah. I am a final year (MRC sponsored) BSc Global Health student at Queen Mary University of London. I am interviewing health workers and mothers in The Gambia to learn about the impacts of Covid-19-related lockdown measures on utilisation of mother and child services. The interview will take about 30 minutes. All the information I obtain will remain strictly confidential. You may choose not to answer any question that makes you feel uncomfortable.

Do you have any questions?

Do you agree to being interviewed? Yes

| **Background** |
| --- |
| 1. **Could you please tell me where you live?**   I live in Brikama Nyambai   1. **What is your profession?**   I am a midwife   1. **What does your role entail?**   We conduct deliveries, we attend to women with conditions such as Eclampsia, and attend to pregnant women during antenatal visits. |
| 1. **Please tell me for how long you have been working in this health facility.**   I have been working here for eleven years now. That was since 2014. |
| 1. **What motivated you into pursuing a public health career?**   I just like the job, but it is not about motivation. I like helping people, but there is not motivation. |
| 1. **What MCH services are provided in this facility? Probe: immunisation, antenatal care**   We provide antenatal services; we also provide infant welfare immediately after birth before referring them to their nearest health facility. So, here we attend to antenatal clinics. Sometimes I do go to the clinic, and I do give service to post delivery with high BP. I counsel them on family planning, and I even provide other family methods such as intra-uterine device,   1. **Did the provision of these services continue during the pandemic?**   The service was provided but was reduced because patients were scared to come. They stayed at home. Even the clinics that we were running, we did not have enough scanning machines because we were trying to avoid this close contact. So, that is why the service was very reduced. This was however only experienced in the antenatal clinics, but not in the delivery ward. But if experience that in the antenatal clinics, this will eventually reflect on deliveries. |
| 1. **Did the health facility stay open during the pandemic, and for how long?**   This health facility was continuously open and was never closed. Even our department was never closed though it hit our staff hard as most of us were infected. |
| 1. **Have you noticed any changes in utilisation of MCH services during the pandemic? For example, do you see fewer or more patients than usual?**   Women were not coming to health facilities during the pandemic because of lack of sensitisation and ignorance. It is now that we started sensitising them but before that, the level of knowledge about the pandemic was very poor. Nurses don’t communicate with their patients, which is very bad. You need to communicate to them so that they understand. If the know, they always apply that knowledge, but if they don’t know, they can’t apply it. Sometimes they would just sit at home and say I will not join antenatal early, if not I will just be going up and down. They don’t know that joining early is important to them, but it is because of ignorance that they say this. That is the only reason because if you ask some of them, that is what they are going to tell you. Covid-19 also made them to stay at home because of the fear of infection in health facilities. Even us here, he used to give them long appointments so as to prevent overcrowding at the health facility. |
|  |
| **Individual factors** |
| 1. **From the perspective of health workers, how safe do you think it is to provide MCH services during the pandemic?**   It was definitely not safe at all because we don’t have much space here. It is just recently that we had an isolation room, but before that, we will be contacting with the patient and attending to them, until later when we will have the results of the patients to inform us whether they are positive or not. It is just recently that we started doing rapid test and PCR test, but before, they will collect your sample and you will wait for week or more before you get your results. So, during all that waiting time, we will be attending to the patient and if the results happen to be positive, they just ask you to go and do self-isolation. So, you go back home infecting your family and put everybody at risk. I was even positive, but luckily, I pulled through. |
| 1. **How safe is for women to access MCH services in this facility at that period?**   It was not safe for them too but because of the need to attend their clinics, that is, why they come, especially the at-risk mothers, because we give them appointments for every two weeks or one week, depending on their condition. It was not the least safe. We were definitely just risking our lives. |
| 1. **Did you or your colleagues work more or less hours during the lockdown? If yes, please explain why?**   At that time, we scaled down and that is when we continued to work for twelve hours and go off for forty-eight hours. This was done to reduce the overcrowding of the staff. So, when two midwives come in the morning, two come in the afternoon. The night staff will also come later at night working for twelve hours. Since then, we continue that scale down. |
| **Interpersonal factors** |
| 1. **What is your family’s attitude in your provision of MCH services during the pandemic? (Are they supportive or not? If yes, explain how?**   All of family was dissuading me from coming to work. They were saying you should stop working because if you continue going to work you will get infected at the hospital. That is, when I was positive, they were all running away from me. so, for them, they were definitely not willing for me to come, especially my husband. He was always taking and saying you should stay at home. So, when I told him I was positive and I need to self-isolate, it was very difficult. If I recall that scenario, I find it very interesting because of the way he was behaving. I did not receive stigma in my community because when I was positive, I kept in me, and it was only my husband and my brother’s wife who knew, I was positive. Even me who were coming to my compound, all stopped coming, but they didn’t know, I was positive. They just knew that I was working in a health facility. So, that was the reason why they stopped coming. |
| 1. **Have you noticed any changes in your colleagues’ attitudes in providing MCH services during the pandemic? probe: did you experience a reduction in staff’s work appetite? If yes, explain why (maybe due to lack of risk allowance and patient overcrowding)**   Yes, I noticed a change, but that wasn’t all of us. Definitely, we are all sacrificing our lives because nobody stayed at home as we all continued coming to work. It was only those that were positive that went to self-isolation. If not that, all of us were coming to work. The motivation among us was very poor but that didn’t affect our work. We were not motivated but that didn’t affect our work.   1. **What incentives were provided by the government to motivate health workers during the pandemic?**   The incentive that we received here was the Covid-19 money which was given to us last year. This was done only one during which we received some money from the government, but that was not even much. Since then, we have not received any allowances from the government. |
| 1. **What is your attitude towards MCH service users during the pandemic? probe: were they making your work easier or more difficult?**   Other can come with short temper but others can come behave politely. We have to do push and pull with them and with their escort as well. During the Covid-19 pandemic, we were saying no escort. So, if you want to receive the patient at the gate and leave the escort outside, it becomes a problem. So, it was a big task for us. They harassed us and quarrelled with us and did all kinds of things. people here don’t believe that Covid-19 really exist in the Gambia, but this is because of ignorance and lack of literacy. |
| **Community factors** |
| 1. **Have you experienced any changes in people’s perception in the community about the use of MCH services during the pandemic? if yes, explain.**   of course, yes because I do see them queuing at the pharmacies. We have a pharmacy near our compound, if I am going to work, I pass by them queuing there and when I asked why they keep queuing here instead of going to the health facility. I even had to speak to the pharmacist and told him that what he is doing is not helping this people, can’t you tell them to the health facility where they will be checked, you are causing more infection in the community than helping them because the place is overcrowded and they were running from the hospital because they believed that if you go to the health facility, they will say you have Covid-19 and will be kept there. |
| 1. **Have you experienced any challenges in providing MCH services due to transport difficulties? if yes, explain how**   For me personally, I did not have any transport difficulties because I was having my own vehicle, but others, they were transport difficulties, especially if they close at night and want to go back home. So, it was always a problem. So, for that reason, we even had a meeting and said that if the hospital does not provide transport for returning staff, we will still down on strike, but with all that, they didn’t provide anything in terms of transport. We just sacrificing ourselves, but definitely, it was a tough time for us. |
| **Institutional factors** |
| 1. **Do you think this health facility had adequate medical supplies during the pandemic? if no, give reasons.**   we did have a shortage of medical supplies, even now have shortage. we used to have shortage of important medicines. This was because of the pandemic. it was difficult as we were controlling our gloves so much. sometimes we will even have a student coming to sacrifice and help but you cannot give opportunity to her because we don’t have enough gloves. Because by right, if you are to conduct a delivery, you have to put on gloves, but for us, we just risk it, wash your hands and apply hand sanitisers. |
| 1. **Do you think this facility had enough manpower to provide MCH services during the pandemic? if no, give reasons**   We experienced a huge shortage of manpower. There was a time when we were having only one midwife on the ground plus the nurse attendants. So, there was huge workload for those remaining here. The time when I was in self-isolation, this place was very bad as you will have only one staff on a shift and patients were coming. Health centres were referring all of their patients to this health facility. If a patient comes to their health facility, they will just tell them to go to Kanifing General Hospital. So, they were just trying to protect themselves and put us at risk. |
| **Policy factors** |
| 1. **Did the lockdown measures, such as curfews and stay at home policies etc., put in place last year had any impact on your use of MCH services during the pandemic? if yes, explain how.**   Before you leave here is around ten and you go to the main and before you have vehicle, especially for those who live far, so it was problem. |
| 1. **To prevent infection in health facilities, infection prevention and control measures, such as mandatory screening, wearing of PPEs and face mask, have been introduced in many health centers. What is the effect of these practices on provision of MCH services?**   At the entrance, there was a hand sanitation point and we were hand sanitizers. It was just that we used to have shortage at times. So, you have to provide it for yourself. When coming into the health facility, women wash their hand at the gate and when they come to us, we give them hand sanitizers. Wearing of face mask was also a matter of most and if you don’t have a face mask, we send you out to go and get a face mask. Before, we used to give them face mask, but it was not enough for everyone. So, we have to ask them to go and buy. So, at the end, they themselves knew that if they don’t come with a face mask, they will be sent out. For some, if you give them a face mask, they will tell you they cannot put on because if they do, they will not be able to breath as they have Asthma. We used to have problems with such people. Sometimes we just ask them to remove it for some seconds and put back on. Some were refusing to come the health facility because of mandatory wearing of face mask. They would say I will not go to the health facility because if I go, they will ask me to put on a face mask. |
|  |
| 1. **Are there any other factors that may have negatively impacted your ability to provide MCH services during the pandemic that I haven’t asked you about? if yes, please state them and explain how?**   Sometimes we run out of gloves and sanitisers and digital thermometer and oxygen.   1. **Are there any other factors that may have contributed to the decline in the use of MCH services during the pandemic that I haven’t asked you about? If yes, please state them.**   It is still because of the Covid-19 pandemic and others because of ignorance as they are not aware. Sometimes we even invite the Red Cross to sensitise them and we sometimes do health education using the media such as through the radio.   1. **To prevent the decline in use and provision of MCH services in the event of another pandemic or second wave, what do you think the government should do?**   The government should have a special hospital for Covid-19. Even if Covid-19 is away, we can use that hospital for other chronic illnesses such as HIV. They should also provide us with the necessary PPEs such as gloves, face mask and hand sanitisers. The government should also try to motivate the staff. It is very important to motivate us as it will make us more motivated. We are just sacrificing ourselves without any motivations. If the motivation is there, others will increase   1. **What advice would you give to people who are not using MCH services during the pandemic?**   We need to sensitise them using the radios and to talk to them when they come for clinics. Maybe those who come will chat with those who didn’t come because they do give information to each other. That one can also help. |
