# Supplementary material for: Factors that influenced utilization of antenatal and immunization services in two local government areas in The Gambia during COVID-19: An interview-based qualitative study
Source: PLoS One. 2023 Jun 29;18(6):e0276357. doi: 10.1371/journal.pone.0276357 (PMC10309596; doi:10.1371/journal.pone.0276357)
Supplement: S1 File — (ZIP) [file pone.0276357.s001.zip › Supporting information /Health worker 10.docx]

In-depth interview questionnaire for health workers

**Introduction and Consent**

Hello, my name is Abdourahman Bah. I am a final year (MRC sponsored) BSc Global Health student at Queen Mary University of London. I am interviewing health workers and mothers in The Gambia to learn about the impacts of Covid-19-related lockdown measures on utilisation of mother and child services. The interview will take about 30 minutes. All the information I obtain will remain strictly confidential. You may choose not to answer any question that makes you feel uncomfortable.

Do you have any questions?

Do you agree to being interviewed? Yes

| **Background** |
| --- |
| 1. **Could you please tell me where you live?**   I am from Farato   1. **What is your profession?**   I am a registered midwife. I studied at the American International University. I graduated in April 2020.   1. **What does your role entail?**   My role here is basically to provide maternal and child health services to women. In the labour ward here, what we basically do here is to conduct deliveries, monitor patients during antenatal visits and after deliveries.  We sometimes provide antenatal services, especially when the Gynaecology department is busy. |
| 1. **Please tell me for how long you have been working in this health facility.**   I have been working here for about eight months now. |
| 1. **Did the provision of these services continue during the pandemic?**   we continued providing the care during the pandemic |
| 1. **Did the health facility stay open during the pandemic, and for how long?**   I heard that it was closed for some time because most of the staff here got infected, including myself. I came from the school and got infected. I was then placed in self-isolation. |
| 1. **Have you noticed any changes in utilisation of MCH services during the pandemic? For example, do you see fewer or more patients than usual?**   in this hospital, the number of deliveries remained unchanged during the pandemic until the hospital was announced to be turned into a Covid-19 treatment centre. It was then that the number of patients coming here reduced as some of the patients were scared to come here. I think that was the only time we experienced a reduction in the number of patients coming here. Even that was just for a week or two. In this facility, there was not much change in the number of patients coming here, especially in the labour ward. |
|  |
| **Individual factors** |
| 1. **From the perspective of health workers, how safe do you think it is to provide MCH services during the pandemic?**   It was not safe at all because the protective gears were not provided. We were all infected, but we were not taken care of. We took care of ourselves. We were neglected by the government. Every day we get in contact with Covid-19 patients here. Maybe this one is going to be positive. Even before our shift today, there were two positive cases that were identified. We were working with them, but even to provide us with the PPEs was a problem. So, it was not safe at all. I think we are naturally immune now. |
| 1. **How safe is for women to access MCH services in this facility at that period?**   It was not safe for them too because in the event that the staff were infected, it will be transmitted to them. This may even prevent some women from coming to health facility because many women, like in the street, when you ask them why they are not coming to the health facility, they would tell you that your health facility is a Covid-19 treatment centre, so we are no more coming there. |
| 1. **Did you or your colleagues work more or less hours during the lockdown? If yes, please explain why?**   They did a scale down. We are still on that scale down because we work for a day usually for twelve hours and then you are off for forty-eight hours. The workload though is too much because many people prefer coming here. The catchment area includes many towns, and it is a referral hospital, even though we are currently not receiving referrals because of the renovation, but when they come, we still receive them. |
| **Interpersonal factors** |
| 1. **What is your family’s attitude in your provision of MCH services during the pandemic? (Are they supportive or not? If yes, explain how?**   My family was very supportive during the pandemic, but I didn’t disclose the fact that I was infected because my mom is old, and she is diabetic and hypertensive. My father is also old. So, I did not want to cause any panic for them. I just took the precautionary measures so as not to infect them. I also gave them medications because at some point they were having some symptoms. So, I decided to give them some medications. that was before the arrival of the vaccines, but they have now taken the vaccine. There were very supportive back then. I even had a child whom they helped me took care of so that I could come to work. For me personally, I was on a study leave during the pandemic, but I wrote to the hospital to inform that I want to come back a help because I saw that many of the health workers were infected but unfortunately, I also got infected when I came back. It was only my husband who did not want me to come, but fortunately, he was not around. So, I decided to come. |
| 1. **What incentives were provided by the government to motivate health workers during the pandemic?**   At what point we were provided with an allowance. So, that helped to motivate the health workers at some point. |
| 1. **What is your attitude towards MCH service users during the pandemic? probe: were they making your work easier or more difficult?**   I can’t say all, but some we had difficult moments with them because they were not cooperative. We don’t blame them that much because of the pain. Some were cooperative and make your live very easy. So, at the end of the day, you have a positive outcome. However, most of them were not following the precautionary measures. Some would come with the mask and if you ask them to adjust the mask to cover both their mouth and nose, they will not even listen to you. There was a time when we were providing the service women without a face mask. There was a time when the infection rate was low. At that time, we were providing the service to women whether you have a mask or not. |
| **Community factors** |
| 1. **Have you experienced any changes in people’s perception in the community about the use of MCH services during the pandemic? if yes, explain.**   Yes, some of my relatives were saying that they will not go the health facility because according to them, when they come here, they will catch the virus. So, it is better for them to take the local treatment than to come to the hospital. |
| 1. **Have you experienced any challenges in providing MCH services due to transport difficulties? if yes, explain how**   Yes, I experienced a huge difficulty because public transport was not operating that much. Drivers take a limited number of people. So, if you don’t come out early, you will not have a vehicle and the fares was very expensive as well. |
| **Institutional factors** |
|  |
| 1. **What do you think of the quality of care provided by this health facility during the pandemic?**   They quality of service was affected because at that time, you come to work scared. This can even lower your immune system. You tend to forget most of the things you should do because you are scared and as such, your brain will not be functioning the way it supposed to. |
| 1. **Do you think this health facility had adequate medical supplies during the pandemic? if no, give reasons.**   In this health facility, they provide the needed medical supplies. w   1. **Do you think this health facility had adequate PPEs during the pandemic? if no, give reasons. Did that have any effect on your willingness or ability to provide MCH services?**   PPEs were not available at that time. it did affect our ability to provide the service but nonetheless we just continued risking our lives in order to provide the service since there is no other way out. We used to run out of surgical gloves, so we have to use other gloves to conduct deliveries. |
| 1. **Do you think this facility had enough manpower to provide MCH services during the pandemic? if no, give reasons**   There was manpower shortage here. Those who were left on the ground experienced many difficulties because most of us got infected. So, we were all self-isolating at home. So, those remaining here had many problems. It even went to the point that they had to close hospital and fumigate the area. So, the manpower was a problem because many of us got infected at the same time. we used to have a new positive case every day among our staff. Although I was not here, I heard that the workload was too much.   1. **What do you think of the health facility environment? Probe: is the facility clean and not overcrowded?**   It was not conducive, but we just tried to improvise. |
| **Policy factors** |
|  |
| 1. **To prevent infection in health facilities, infection prevention and control measures, such as mandatory screening, wearing of PPEs and face mask, have been introduced in many health centers. What is the effect of these practices on provision of MCH services?**   They did have an effect because wearing face mask and hand washing helped to reduce the infection rate. If these measures are done properly, then we will all be protected and feel motivated. |
| 1. **What is the effect of these measures on utilisation of MCH services during the pandemic?**   These measures may prevent others from coming because some would tell you that they cannot breathe with the face mask on. This might have some effect on their willingness to come to the health facility because it is compulsory. So, they would rather stay at home. |
| 1. **Are there any other factors that may have negatively impacted your ability to provide MCH services during the pandemic that I haven’t asked you about? if yes, please state them and explain how?**   The main barrier we had here is the lack of PPEs, such as gloves. Some women were also not cooperative at all.   1. **To prevent the decline in use and provision of MCH services in the event of another pandemic or second wave, what do you think the government should do?**   The government should increase their Covid-19 treatment service. We cannot be working here with positive cases. We are not protected. They need to provide an isolation centre for positive cases. You cannot be positive and continue using the same toilet with other pregnant women. There is no isolation room in this health facility.   1. **What advice would you give to people who are not using MCH services during the pandemic?**   they should come to their clinics regularly because some of them may have an underlying health problem and for some women, even the panic and the constant fear may raise their blood pressure. For the majority of women who die during labour is because of raised blood pressure, which leads to postpartum haemorrhage. So, if you don’t come to the clinic, how can we check and monitor your blood pressure. So, they should always come to the health facility. |
