# Supplementary material for: Factors that influenced utilization of antenatal and immunization services in two local government areas in The Gambia during COVID-19: An interview-based qualitative study
Source: PLoS One. 2023 Jun 29;18(6):e0276357. doi: 10.1371/journal.pone.0276357 (PMC10309596; doi:10.1371/journal.pone.0276357)
Supplement: S1 File — (ZIP) [file pone.0276357.s001.zip › Supporting information /Respondent 13.docx]

In-depth Interview Questionnaire for MCH service Users

**Introduction and Consent**

Hello, my name is Abdourahman Bah. I am a final year (MRC sponsored) BSc Global Health student at Queen Mary University of London. I am interviewing health workers and mothers in The Gambia to learn about the impacts of Covid-19-related lockdown measures on utilisation of mother and child services. The interview will take about 30 minutes. All the information I obtain will remain strictly confidential. You may choose not to answer any question that makes you feel uncomfortable.

Do you have any questions?

Do you agree to being interviewed? Yes

| **A** | **Background** |  |
| --- | --- | --- |
| 1 | **Could you please tell me where you live – Probe: house of residence is?**  I am from Brikama Bussura | |
| 2 | **Please tell me how you got here today? Probe: public transport, private or walked.**  I used public transport | |
| 3 | **Have you used MCH services during the pandemic? Probe: immunisation, antenatal consultations etc.**  Yes, I used to bring my child for immunisation. | |
| 5 | **Have you changed the way you access this service during the outbreak? If so, how? If you have changed, are you going more times or less times?**  I used to bring my child for immunisation, but there came a time when they stopped us from coming because of the Covid-19 pandemic. | |
| **B** | **Individual factors** |  |
| 7 | **How safe do you think it is to access MCH services during the pandemic? - Probe: have these concerns stopped you from using these health facilities?**  I can say it was safe but at the same time not that safe. If you have someone who would immunise your child at home is easier than if having to pay public transport to come to the health facility. It was also not safe to come to the health facility because you can easily get infected at the health facility. I wasn’t coming regularly because the health facility used to be overcrowded, which made the environment unsafe. | |
| 8 | **Have you experienced any financial difficulties (e.g., transport costs) in accessing MCH services during the pandemic? if yes, explain.**  I did not experience transport difficulties, but transport fares were increased at that time. There was also a shortage of vehicles, which made it very difficult to get here during that period of time. | |
| **C** | **Interpersonal factors** |  |
| 9 | **What is your family’s attitude, including your husband, in your use of MCH services during the pandemic? Probe: Do they encourage or discourage you? In what way?**  It is me who makes the decision to come here, and he supports me whenever I wish to come. However, some of my family members were saying I should not come because of the high transport fares that I have to pay to get here. Some were also saying I shouldn’t come because the health facility was not safe during the pandemic. | |
| 10 | **Have you noticed any changes in your friends’ attitudes in use of MCH services during the pandemic?**  Some of my friends were not bringing their children for immunisation during the pandemic. There were not coming because of the high fares they have to pay to get here. | |
| **D** | **Community factors** |  |
| 11 | **Have you noticed any changes in people’s perception in your community about the use of MCH services during the pandemic? if yes, explain. Probe: give examples of people being afraid of visiting facilities due to stigma associated with visiting health facilities or fear of being quarantined etc.**  In my village, there were also some people who were not coming for MCH services during the pandemic. They were also no coming to the health facility because of high transport fares. | |
| **E** | **Institutional factors** |  |
| 14 | **Did the health facilities stay open during the pandemic? if no, state how this may have affected your access to MCH services.**  The health facility that I used to visit was closed for some time. It was closed because of the Covid-19 pandemic. When I come and find it close, I would go back home and come back the following month. When I come back the following month, they would complain about why I missed the last month. When it is not actually my fault, not mine. | |
| 15 | **How satisfied are you with the care provided by this health facility during the pandemic? probe: consultation time, treatment and respect from health workers.**  When I come here during the pandemic, I used to queue here for a long period of time. | |
| 16 | **Do you think this health facility has adequate medical supplies during the pandemic? if no, give reasons.**  They don’t have the medicines that are needed. They would usually prescribe some medicines that you would have to buy at the pharmacy, where it is too costly. | |
| 18 | **What are your perceptions about the health workers in this facility? (e.g., competence or behaviour of health workers)**  When delivering, they would tell you abusive words, especially the women health workers. They say things that would burn you, when you’re suffering during labour, but this not all the health workers, as some do not have any problems. | |
| **F** | **Policy factors** |  |
| 20 | **Did the lockdown measures, such as stay at home policies, travel bans, etc, put in place last year had any impact on your use of MCH services during the pandemic? if yes, explain how.**  Yes, sometimes when you come here, they would tell you they cannot take you since they have reached the threshold for that day. I would come and they would sometimes ask to go back. | |
| 21 | **To prevent infection in health facilities, infection prevention and control measures, such as mandatory screening and wearing of facemask, have been introduced in many health centers. What is the effect of these practices on waiting time and quality of service?**  If you don’t have a face mask, you will not be allowed entry into the health facility. They also asked everyone to wash their hands at the entrance before getting in. I am not very much comfortable wearing a face mask. I used to put on when entering the health facility, but quickly remove it when I get in. | |
| 23 | **What do you think the government should do to prevent a decline in use of MCH services in the event of another pandemic?**  I would advise them to educate people about the pandemic as well as the importance of vaccinating children. | |
| 24 | **What advice would you give to people who were not using MCH services during the pandemic?**  I would advise them to bring their children for immunisation. Transport fares should not prevent them from bringing their children for immunisation because of it is importance for the wellbeing of their children. | |
