# Supplementary material for: Factors that influenced utilization of antenatal and immunization services in two local government areas in The Gambia during COVID-19: An interview-based qualitative study
Source: PLoS One. 2023 Jun 29;18(6):e0276357. doi: 10.1371/journal.pone.0276357 (PMC10309596; doi:10.1371/journal.pone.0276357)
Supplement: S1 File — (ZIP) [file pone.0276357.s001.zip › Supporting information /Respondent 14.docx]

In-depth Interview Questionnaire for MCH service Users

**Introduction and Consent**

Hello, my name is Abdourahman Bah. I am a final year (MRC sponsored) BSc Global Health student at Queen Mary University of London. I am interviewing health workers and mothers in The Gambia to learn about the impacts of Covid-19-related lockdown measures on utilisation of mother and child services. The interview will take about 30 minutes. All the information I obtain will remain strictly confidential. You may choose not to answer any question that makes you feel uncomfortable.

Do you have any questions?

Do you agree to being interviewed? Yes

| **A** | **Background** |  |
| --- | --- | --- |
| 1 | **Could you please tell me where you live – Probe: house of residence is?**  I am from Brikama Farabasu | |
| 2 | **Please tell me how you got here today? Probe: public transport, private or walked.**  I used public transport | |
| 3 | **Have you used MCH services during the pandemic? Probe: immunisation, antenatal consultations etc.**  During the peak of the pandemic, I was pregnant. I used to come for antenatal care at that time. | |
| 5 | **Have you changed the way you access this service during the outbreak? If so, how? If you have changed, are you going more times or less times?**  I used to come for antenatal care but there came a time when we were told not to come every month. When you come one month, you should not come the following month. For that reason, I didn’t come for antennal care for about three months. When I re-started coming, the health workers asked why I wasn’t coming for past three months. I replied, you told us not to come until the pandemic has cooled down. When coming at that time, only two people were allowed to sit on one bench instead of five and you have to wash your hands at the entrance or apply a hand sanitiser. You also need to put on a face mask without which you will not be allowed entry and will be asked to go home and get one. They complained about my absence for the past three months, but I told them I wasn’t coming because they were ones who told us not come until things has improved. I was also afraid to come as they used to say you can even get infected with Covid-19 through handshake and coughing. That is also why I wasn’t coming for antennal care. | |
| **B** | **Individual factors** |  |
| 7 | **How safe do you think it is to access MCH services during the pandemic? - Probe: have these concerns stopped you from using these health facilities?**  Off course it wasn’t safe to come to the health facility during the pandemic. when coming, my parents would tell me “You know we are living in the pandemic, so if you go out you will get infected with Covid-19 and even at the health facility, some people may have gotten infected and will not tell others that they have it and they will continue sitting close to you, knowing that they have it”. These were just some of the things my parents were afraid off. | |
| 8 | **Have you experienced any financial difficulties (e.g., transport costs) in accessing MCH services during the pandemic? if yes, explain.**  Transport was huge challenge at that time. Even today, transport is a big problem. Where I live is very far from the health facility. As such, I pay many fares. There is also a vehicle shortage. this was even worse during the pandemic as social distancing was introduced in every vehicle as such drivers had to take only few passengers. You also had to pay double fares at that time. | |
| **C** | **Interpersonal factors** |  |
| 9 | **What is your family’s attitude, including your husband, in your use of MCH services during the pandemic? Probe: Do they encourage or discourage you? In what way?**  My mother used tell me “You know that Covid-19 exist, so you should not go out often”. All of my siblings were also afraid to go out at that time, especially during the lockdown as most people were afraid to go out at that time. My husband wasn’t as afraid as my mother, but he used to tell me, when you go to the health facility, you just make sure that neither you nor my child get infected. He would also say “you know about the weighing; all kinds of children will be placed on the same weighing machine. The child can easily get infected there and spread it to the rest of the family. This is because there is only one weighing machine at the health facility where all the children that are bought for immunisation are placed to monitor their growth. This is what my husband used to complain about, but despite these fears, he never prevented me coming for MCH services. | |
| 10 | **Have you noticed any changes in your friends’ attitudes in use of MCH services during the pandemic?**  Yes, during the lockdown last year, many people stopped coming for MCH services. when I tell my friends to come MCH services, they would tell me they would not go because of the pandemic. Me, personally, I never saw a person who go infected with Covid-19. I only saw it on T.V. At that time, whoever dies, they would say it is because of Covid-19. A friend of mine also told me one her friends since her delivery which is three months ago, she only brought her child once for immunisation. | |
| **D** | **Community factors** |  |
| 11 | **Have you noticed any changes in people’s perception in your community about the use of MCH services during the pandemic? if yes, explain. Probe: give examples of people being afraid of visiting facilities due to stigma associated with visiting health facilities or fear of being quarantined etc.**  At that time, only few people were coming to health facilities. All those who were not coming, they said it is because of the pandemic. they would say “Gambia is a small country, so if one person gets infected, everyone in the country will get it. When one person died from Covid-19 in Kanifing General Hospital, people stopped going to that hospital due to fear of infection. For me personally, when I got home, I would always wash myself and my child before doing anything else. | |
| 12 | **Has this had any impact on your use of MCH services during the pandemic? if yes, explain how**  What people were saying did not prevent me from coming to health facilities because I have never seen a positive case of Covid-19 with my own eyes. During the peak of the pandemic, I was pregnant at that, but that I still continued coming. At that time, I was going to Fagikunda health centre. I only followed the Covid-19 precautions such as wearing of face mask and washing my hands. | |
| **E** | **Institutional factors** |  |
| 14 | **Did the health facilities stay open during the pandemic? if no, state how this may have affected your access to MCH services.**  They hospital was open but there came a time when they would tell people not to come for MCH services. However, they health workers later told us that, they didn’t deliver such information. They also introduced social distancing at the health facility. They also introduced a limit on the number of people they would allow in the health facility a day, so if you don’t come early, you will not receive the service. For this reason, many people stopped going to the health facility. Even myself, there was a time I stopped going to the health facility. When they asked me, why I wasn’t coming, I replied it is because whenever I come here you would have stopped seeing people for that day. There was a month that I didn’t go to the health facility because I was told my someone that the health facility is currently closed, when I went the following month, they asked me why I wasn’t coming, I told them I was told the health facility was closed. They told me, that was true as they never closed the health facility since they people may have emergency at any moment. | |
| 15 | **How satisfied are you with the care provided by this health facility during the pandemic? probe: consultation time, treatment and respect from health workers.**  The health facility that I used to visit used to be overcrowded. To reduce this, they started seeing a community by community. So, if you missed the day allocated for your community, you will not be offered any service on another day. They would tell you; you have missed the day allocated for your community so you will have to wait until the following month. During the pandemic it was not very crowded, so we did not have to wait for long. It was just that we had to observe social distancing. | |
| 16 | **Do you think this health facility has adequate medical supplies during the pandemic? if no, give reasons.**  Even before the Covid-19 pandemic, I would not have all the medical supplies that I need. If they prescribe some medicines for you, you will have few in the health facility and you have to buy the rest in a pharmacy outside. The only medicines that they will give you is paracetamol. The shortage of medical supplies in the health facilities is something normal here. | |
| 17 | **Do you think this facility has enough manpower to provide MCH services during the pandemic? if no, give reasons**  At the health facility that I used to visit, there weren’t many health workers at that time, but there were some students give them a hand. | |
| **F** | **Policy factors** |  |
| 20 | **Did the lockdown measures, such as stay at home policies, travel bans, etc, put in place last year had any impact on your use of MCH services during the pandemic? if yes, explain how.**  Yes, these measures affected many people. During that time, I stopped coming for antennal care for three months and stopped going out. People were not allowed to go out once the curfew comes into effect. | |
| 21 | **To prevent infection in health facilities, infection prevention and control measures, such as mandatory screening and wearing of facemask, have been introduced in many health centers. What is the effect of these practices on waiting time and quality of service?**  Some people don’t like wearing face mask. Even myself, when I put on a face mask, I cannot breathe properly. Despite this, people had to wear face mask in order to enter the health facility. This may have prevented some people from coming to health facilities since they will not be allowed without a face mask. I didn’t tell them I wasn’t comfortable wearing a face mask as they may not understand my situation. | |
| 23 | **What do you think the government should do to prevent a decline in use of MCH services in the event of another pandemic?**  The government should help people with medicines so that when our children get sick, they will get the medicines they need. Some people don’t have money to buy medicines. Some may be able to afford it, but some may not. | |
| 24 | **What advice would you give to people who were not using MCH services during the pandemic?**  I would advise them to bring their children for immunisation because vaccinating their children is good for the wellbeing of their children as it protects from vaccine preventable diseases. | |
