# Supplementary material for: Factors that influenced utilization of antenatal and immunization services in two local government areas in The Gambia during COVID-19: An interview-based qualitative study
Source: PLoS One. 2023 Jun 29;18(6):e0276357. doi: 10.1371/journal.pone.0276357 (PMC10309596; doi:10.1371/journal.pone.0276357)
Supplement: S1 File — (ZIP) [file pone.0276357.s001.zip › Supporting information /Respondent 15.docx]

In-depth Interview Questionnaire for MCH service Users

**Introduction and Consent**

Hello, my name is Abdourahman Bah. I am a final year (MRC sponsored) BSc Global Health student at Queen Mary University of London. I am interviewing health workers and mothers in The Gambia to learn about the impacts of Covid-19-related lockdown measures on utilisation of mother and child services. The interview will take about 30 minutes. All the information I obtain will remain strictly confidential. You may choose not to answer any question that makes you feel uncomfortable.

Do you have any questions?

Do you agree to being interviewed? Yes

| **A** | **Background** |  |
| --- | --- | --- |
| 1 | **Could you please tell me where you live – Probe: house of residence is?**  I am from Brikama Nyambai | |
| 2 | **Please tell me how you got here today? Probe: public transport, private or walked.**  I used public transport | |
| 3 | **Have you used MCH services during the pandemic? Probe: immunisation, antenatal consultations etc.**  Yes, during the pandemic, I used to bring my child for immunisation | |
| 5 | **Have you changed the way you access this service during the outbreak? If so, how? If you have changed, are you going more times or less times?**  I was coming less during the pandemic. I used to come only when the child was to be injected. They would usually send us a text to tell us the child is due for vaccination. However, this wasn’t the case for long time. It was only during the peak period of the pandemic. one would not come if one were not sent a text because at that time, they had stopped weighing children. It was only vaccinations that were offered. So, you don’t come if your child doesn’t have vaccinations. | |
| **B** | **Individual factors** |  |
| 7 | **How safe do you think it is to access MCH services during the pandemic? - Probe: have these concerns stopped you from using these health facilities?**  In my opinion, it wasn’t safe to access MCH services during the pandemic. however, people were this risk because of the importance of vaccinating their children. The children cannot put on a mask, so it is us the mothers who had to wear face mask. So, it was a huge risk to come here for both the mothers and their children since only the mothers can wear face mask. For me personally, I knew it was not safe, but I was coming just for the importance of vaccinating my child. | |
| 8 | **Have you experienced any financial difficulties (e.g., transport costs) in accessing MCH services during the pandemic? if yes, explain.**  Travelling at that time was very difficult. This is because the drivers at that time were reluctant to go to work because of the Covid-19 restrictions. For that reason, it was very difficult to get a vehicle and sometimes, I would have to walk to the health facility from my home despite the long distance. Nonetheless, this difficulty did not prevent me from bringing my child for immunisation. | |
| **C** | **Interpersonal factors** |  |
| 9 | **What is your family’s attitude, including your husband, in your use of MCH services during the pandemic? Probe: Do they encourage or discourage you? In what way?**  During that period, my husband used to help me a lot because when men bring their children to the health facility, they are exempted from queuing. So, for that reason, instead of me bringing our child to the health facility, my husband would bring the child for immunisation and get home quickly. If he wasn’t going to work on that day, he would always take our child for immunisation. So, he was very helpful | |
| 10 | **Have you noticed any changes in your friends’ attitudes in use of MCH services during the pandemic?**  No, I haven’t | |
| **D** | **Community factors** |  |
| 11 | **Have you noticed any changes in people’s perception in your community about the use of MCH services during the pandemic? if yes, explain. Probe: give examples of people being afraid of visiting facilities due to stigma associated with visiting health facilities or fear of being quarantined etc.**  Yes, I have seen some people in my community who were refusing to bring their children for immunisation during the Covid-19 pandemic. When returning home from the health facility, some would even ask me if immunisations were still ongoing. They would say they are afraid to bring their child to the health facility. These are just some examples of people being afraid to bring their children to health facilities. The reason they would give is that it was not safe to come to health facilities because of the overcrowding in health facilities. So, they preferred to stay at home than to mingle with the large crowd in the health facilities. | |
| 12 | **Has this had any impact on your use of MCH services during the pandemic? if yes, explain how**  These change of perception in the community didn’t not impact my willingness to bring my child for immunisation. This because everyone has their own perceptions about the pandemic and for them, that’s how they saw it. For me, I prioritize the health of my child despite the fact that I was putting him at risk of contracting Covid-19 since he couldn’t wear face mask. | |
| **E** | **Institutional factors** |  |
| 14 | **Did the health facilities stay open during the pandemic? if no, state how this may have affected your access to MCH services.**  When I was coming here, it was always open. | |
| 15 | **How satisfied are you with the care provided by this health facility during the pandemic? probe: consultation time, treatment and respect from health workers.**  I was pleased to see that they banned weighing children during the pandemic. This made me feel safe as it reduced the chances of transmitting the disease from one child to another. | |
| 16 | **Do you think this health facility has adequate medical supplies during the pandemic? if no, give reasons.**  The queue used to be long here during the pandemic but was a bit reduced as some were not bringing their children from immunisation. The availability of medicines here is not reliable as sometimes you have all the medicines you need but at times, you will have to buy them at the pharmacy. However, it is worth noting that this was not only the case during the pandemic but is something that happen on regular basis here. | |
| 17 | **Do you think this facility has enough manpower to provide MCH services during the pandemic? if no, give reasons**  During the Covid-19 pandemic, there were very few health workers here. I don’t know what the reason was, but they were not many at time. As result, we would wait here a long period before we can receive the service we came for. Although, the number of people bringing their children for immunisation declined, the waiting time increased due to the decline in the number of health workers. | |
| **F** | **Policy factors** |  |
| 20 | **Did the lockdown measures, such as stay at home policies, travel bans, etc, put in place last year had any impact on your use of MCH services during the pandemic? if yes, explain how.**  This did not have any effect on my use of MCH services during the pandemic because even before the introduction of these measures, I was already not going out often. I only go for important things such going to the market and for other things. | |
| 21 | **To prevent infection in health facilities, infection prevention and control measures, such as mandatory screening and wearing of facemask, have been introduced in many health centers. What is the effect of these practices on waiting time and quality of service?**  Initially, the wearing of face mask was huge challenge for me, and it affected me badly. At that, the wearing of face mask was very strict here and people were not even allowed not cover their noses. The health workers were always monitoring people to make sure that they cover both mouth and nose with no exceptions. The wearing of face mask was therefore a big difficulty for me since we had to wear it for a long period of time due to the long waiting time. The other measures though did not have effect on me. | |
| 22 | **Have these measures had any impact on your use of MCH services during the pandemic? if yes, explain how**  Some of these measures also contributed to the decline in the number of people using MCH services during the pandemic. For example, I know someone who stopped bringing her child for immunisation because of the mandatory wearing of face mask at health facilities. She told me that wearing face mask affects her breathing, as such she would rather stay at home and not wear a face mask than bring her child for immunisation. | |
| 23 | **What do you think the government should do to prevent a decline in use of MCH services in the event of another pandemic?**  The government should try to sensitize people about the importance of vaccinating children because at that time they were only particular about the pandemic and not focusing on the importance of vaccinating children. They were only concentrating on one aspect and forgetting other aspects. Therefore, my advice to the government would be not forget about encouraging mothers to take their children for immunisation since it is something very important for the wellbeing of their children. | |
| 24 | **What advice would you give to people who were not using MCH services during the pandemic?**  For women who were not bringing their children for immunisation during the pandemic, my advice to them would be to bring their children for immunisation because it is very important for their children. The advice goes both to mothers and pregnant women as it is important for the health of both children and pregnant women. For that reason, they should all take it very seriously. | |
