# Supplementary material for: Factors that influenced utilization of antenatal and immunization services in two local government areas in The Gambia during COVID-19: An interview-based qualitative study
Source: PLoS One. 2023 Jun 29;18(6):e0276357. doi: 10.1371/journal.pone.0276357 (PMC10309596; doi:10.1371/journal.pone.0276357)
Supplement: S1 File — (ZIP) [file pone.0276357.s001.zip › Supporting information /Health worker 3.docx]

In-depth interview questionnaire for health workers

**Introduction and Consent**

Hello, my name is Abdourahman Bah. I am a final year (MRC sponsored) BSc Global Health student at Queen Mary University of London. I am interviewing health workers and mothers in The Gambia to learn about the impacts of Covid-19-related lockdown measures on utilisation of mother and child services. The interview will take about 30 minutes. All the information I obtain will remain strictly confidential. You may choose not to answer any question that makes you feel uncomfortable.

Do you have any questions?

Do you agree to being interviewed? Yes

| **Background** |
| --- |
| 1. **Could you please tell me where you live?**   I live in Brikama.   1. **What is your profession?**   I am a nurse   1. **What does your role entail?**   For now, as far as the maternity is concerned, we are concerned with maternity cases, deliveries and other cases that are concerned with maternity. |
| 1. **Please tell me for how long you have been working in this health facility.**   I have been working here for about four years now. |
| 1. **What motivated you into pursuing a public health career?**   Nursing is just my passion, I love nursing. Whenever I visit a health facility, I see them, and I feel happy. The act of caring for someone who is need of that care. I just love it. |
| 1. **What MCH services are provided in this facility? Probe: immunisation, antenatal care**   They do provide antenatal services for pregnant mothers, infant and child clinics, and we do have personal care and conduct deliveries and other emergency cases as far as maternity is concerned.   1. **Did the provision of these services continue during the pandemic?**   As far as I know, in this maternity the service provision continued. |
| 1. **Did the health facility stay open during the pandemic, and for how long?**   The health facility was opened all the time. |
| 1. **Have you noticed any changes in utilisation of MCH services during the pandemic? For example, do you see fewer or more patients than usual?**   As far as I know, in this labour ward, people were coming in fact some people were leaving their health facilities to come here instead. Because according to them, if they go to their health facilities, they are told that there is Covid-19 so they are trying to reduce the number of patients coming to their health facility. So for us, whoever comes here we manage. I could even remember when the first suspected case came from Senegal. She was brought here, where she was isolated. We didn’t reduce the number of people coming here. The service follow was just normal to me. the reduction in the number of people coming depends on the season. Let us say, in a particular season, usually in September deliveries are high but at this time of the year, you can sometimes go a whole day without any delivery or just one delivery. In September or in other months, we can have up to twenty deliveries a day. To my observation, that has to do with the season but has noting do with the pandemic. |
|  |
| **Individual factors** |
| 1. **From the perspective of health workers, how safe do you think it is to provide MCH services during the pandemic?**   It was not safe at all because we didn’t have the gears. I could remember, there was a day I was here, we had a suspected case from Senegal. I had to call my people and tell them that I would not attend to the patient because there was no PPEs available. So, I was not going to put my life at risk. I had to go to the store and tell them to give us some PPEs. Imagine they had all these things in the store but was not released to us, so we had to protest on that particular day for it to be given to us. So, actually it was not safe at all for anybody especially for me, as I know of myself, because we didn’t have any PPEs. Even up to now, you need to buy mask with your own money. Sometimes they will have it in the store but will not give to people, so we sometimes have to be stubborn to get it. |
| 1. **How safe is for women to access MCH services in this facility at that period?**   It was not safe for them too, because it was not safe for us so how can it be safe for them. For them, they were not really concerned with the protocols as they were not following the protocols. In fact, it was difficult as for some of them when they come if you ask them to wear a face mask, they will tell you they cannot put on a face because they would not be able to breath with the mask on. So, that also makes it unsafe for rest of us. |
| 1. **Did you or your colleagues work more or less hours during the lockdown? If yes, please explain why?**   There was a particular time when they had to cut down the number of staff on the ground. We used to work for a certain number of hours for three days in a week before going off for about two days. This was done to reduce the number of people interacting on the same shift. I think the number of hours remained unchanged but the number of working days was reduced. |
| **Interpersonal factors** |
| 1. **What is your family’s attitude in your provision of MCH services during the pandemic? (Are they supportive or not? If yes, explain how?**   I didn’t have any issues with my family with regard to coming to work at that time. I could remember when I was working at night, my colleague who I was working with, we both took the test on the same day, and she tested positive. We were both eating together and shared things including cups. So, I had to go home and self-isolate for one week. So, my family was actually very supportive, including my husband, my mom and dad. |
| 1. **Have you noticed any changes in your colleagues’ attitudes in providing MCH services during the pandemic? probe: did you experience a reduction in staff’s work appetite? If yes, explain why (maybe due to lack of risk allowance and patient overcrowding)**   Some of my colleagues went to the extent of getting discouraged of providing the service, but they did not stop providing the service. Some were really demotivated, but some were really very strong and remained motivated.   1. **What incentives were provided by the government to motivate health workers during the pandemic?**   We were given some cash sometime a go to motivate us. This helped to motivate nurses. As you know, how poor Gambian nurses are, so if they are given some money at that difficult moment, that would really boost their moral. |
| 1. **What is your attitude towards MCH service users during the pandemic? probe: were they making your work easier or more difficult?**   Some were not following the precautionary measures. For some, if you ask them to wear a face mask, they will tell you if I wear my face mask, I will not be able to breath. So, most of them based it on that. Others would also tell you that they are Asthmatic, and as such cannot wear a face mask. These were some of the excuses they were given us. We insisted on telling them that it is for their own safety. They were really making our lives difficult. |
| **Community factors** |
| 1. **Have you experienced any changes in people’s perception in the community about the use of MCH services during the pandemic? if yes, explain.**   To be honest with you, I know very little of what is happening in my area because when I close from work I am always at home. The few people that I attend to or those who come to my house were telling me that they would not go the hospital because if you do, they will tell you that you have Corona. So, for that reason, some were trying to run away from health facilities. |
| **Institutional factors** |
|  |
| 1. **What do you think of the quality of care provided by this health facility during the pandemic?**   As far as I know, in this place, the quality of service was not impacted that much by the pandemic. as long as this area is concerned, the quality of service remained the same. |
| 1. **Do you think this health facility had adequate medical supplies during the pandemic? if no, give reasons.**   The little medical supplies that we needed was available during the pandemic.   1. **Do you think this health facility had adequate PPEs during the pandemic? if no, give reasons. Did that have any effect on your willingness or ability to provide MCH services?**   The shortage of PPEs did impact our ability to provide the service because in certain cases, you will not render the service that is expected of you because you are tying to protect yourself. You are getting in contact with the patient and the patient maybe coughing at you, so if you don’t have the necessary PPEs, something must be absent in the care you provide. |
| 1. **Do you think this facility had enough manpower to provide MCH services during the pandemic? if no, give reasons**   When they started the scale down, we experienced a shortage in manpower. Currently, we have about three or four midwives here, but during the pandemic, instead of four midwives, we used to have only two midwives on the ground. This was because two would go on scale down for two to three days and the other two will be here. So, the manpower was massively reduced. We had some cases among our colleagues, so that had another effect, and some were also in self isolation. Nonetheless, we managed to provide the service continuously. The workload also increased as some were leaving their health facilities to come here. Some were leaving all the way from Senegal to come here. So, I can say the workload increased.   1. **What do you think of the health facility environment? Probe: is the facility clean and not overcrowded?**   The security guards were doing their best. They made sure that people wear face mask before entering the hospital, but even with that, we had some challenges as some automatically refused to wear their face mask, so it was challenging for us. |
| **Policy factors** |
|  |
| 1. **To prevent infection in health facilities, infection prevention and control measures, such as mandatory screening, wearing of PPEs and face mask, have been introduced in many health centers. What is the effect of these measures on utilisation of MCH services during the pandemic?** |
| I could remember I had a patient who came in but was not wearing a face mask and I told her to go and get one as she cannot come here without a mask. So, the woman was upset and left. I overheard her saying that I will go to another health facility as this is not the only health facility in the country. |
| 1. **Are there any other factors that may have negatively impacted your ability to provide MCH services during the pandemic that I haven’t asked you about? if yes, please state them and explain how?**   I think the main barrier we had here was the shortage of PPEs.   1. **Are there any other factors that may have contributed to the decline in the use of MCH services during the pandemic that I haven’t asked you about? If yes, please state them.**   As I told you before, they would tell you that if I go a health facility, they will tell you that you have Covid-19, and I will be quarantined. I think those were the main reasons.   1. **To prevent the decline in use and provision of MCH services in the event of another pandemic or second wave, what do you think the government should do?**   The government needs to sensitise people because people are lacking knowledge about Covid-19. Some are still not taking Covid-19 seriously update and they believe Covid-19 does not exist in The Gambia. They should also make sure that they provide enough PPEs for health workers. If PPEs are not available, adequate service will not be rendered because they are certain things I for one will not do for the patient. If I were to monitor a patient every thirty minutes, I will increase it to one hour because I will try to reduce the exposure time with the patient. So, if I have the necessary PPEs, I will not be scared to get close to the patient, but if the PPEs are not there and I have a family and hypertensive father, I will obviously not put myself and my family at risk. My family is more important to me than the patient. So, if they want things to go fine then should provide adequate PPEs to health workers.   1. **What advice would you give to people who are not using MCH services during the pandemic?**   As I said, let them continue sensitising women, including pregnant women, to protect themselves like how the health workers protect themselves. The government should tell them we try to protect the health workers, so we protect them, you should also protect yourselves. They need to continue sensitising people as we have a long way to go as long as Covid-19 is concerned. The information we have is not enough as people are still lacking knowledge about Covid-19. |
