# Supplementary material for: Factors that influenced utilization of antenatal and immunization services in two local government areas in The Gambia during COVID-19: An interview-based qualitative study
Source: PLoS One. 2023 Jun 29;18(6):e0276357. doi: 10.1371/journal.pone.0276357 (PMC10309596; doi:10.1371/journal.pone.0276357)
Supplement: S1 File — (ZIP) [file pone.0276357.s001.zip › Supporting information /Respondent 1.docx]

In-depth Interview Questionnaire for MCH service Users

**Introduction and Consent**

Hello, my name is Abdourahman Bah. I am a final year (MRC sponsored) BSc Global Health student at Queen Mary University of London. I am interviewing health workers and mothers in The Gambia to learn about the impacts of Covid-19-related lockdown measures on utilisation of mother and child services. The interview will take about 30 minutes. All the information I obtain will remain strictly confidential. You may choose not to answer any question that makes you feel uncomfortable.

Do you have any questions?

Do you agree to being interviewed? Yes

| **Background** |
| --- |
| 1. **What is your ethnicity?**   I am a Fula   1. **Could you please tell me where you live – Probe: house of residence is?**   I am from Busumbala |
| 1. **Please tell me how you got here today? Probe: public transport, private or walked.**   My brother brought me here in his vehicle   1. **Have you gone to the health facility during the COVID-19? Were you treated differently? Were there any different procedures? If so, what are they doing that is different?**   I have gone to a health facility during the pandemic, but I was afraid. I can remember, I once had an appointment with a doctor, but I did go because I was of getting infected. For that being the case, I was afraid to use the contraceptive injection and went and bought the contraceptive tablet as a result I had an unplanned pregnancy for this baby. I had this baby because of that Covid-19 because I took the pills and went to the pharmacy because I didn’t want to go to the health facility. So, I went straight to the pharmacy, and I bought the contraceptive tablet. So, unfortunately for me I had this pregnancy, which was very sad for me. I was very frustrated at that moment. I even wanted to abort it. I even called two gynecologists and one of them said he was not the right person. It was because of this that I didn’t abort. This is all because of the Covid-19 pandemic and which has affected my education. Right now, I am studying at the university of The Gambia but because of my unplanned pregnancy I could not finish my exams. When you go to the health facility, they would tell you to put on a face mask and maintain social distancing and taking people’s temperatures. |
| 1. **Have you used MCH services during the pandemic? if yes, what MCH service have you used during the pandemic?**   During the peak of the pandemic, I was pregnant, but I was not coming for antenatal service until after six months of my pregnancy. Then, I was really sick as I had anaemia and could not walk for a long distance. So, I went to the health facility. That was the time I joined the antenatal clinic. During the pandemic, I was not coming to the health facility that often. This was because of fear of getting infected. |
|  |
|  |
| **Individual factors** |
| 1. **How safe do you think it is to access MCH services during the pandemic? - Probe: have these concerns stopped you from using these health facilities?**   In my opinion, it was not safe as that was the reason, I was not coming to the health facility. So, I got all these problems because of that. |
| 1. **Have you experienced any financial difficulties (e.g., transport costs) in accessing MCH services during the pandemic? if yes, explain. Probe- have these difficulties stopped you from using these health facilities?**   I did not have transport difficulties at that time because my husband has a car. At that time I was going to Brikama District hospital. |
| **Interpersonal factors** |
| **18.What is your family’s attitude, including your husband, in your use of MCH services during the pandemic? Probe: Do they encourage or discourage you? In what way?**  My family was supportive as my husband used to bring me to the hospital in his vehicle, but I used to hear people saying in the media that it is not safe to come to the health facility. |
|  |
| **Community factors** |
| **20.Have you noticed any changes in people’s perception in your community about the use of MCH services during the pandemic? if yes, explain. Probe: give examples of people being afraid of visiting facilities due to stigma associated with visiting health facilities or fear of being quarantined etc.**  Yes, because in the beginning of the pandemic, many people were afraid to go to the health facility. I could remember when one of my children was sick, I took him to the pharmacy instead of going to the health facility. Also, one of my relatives died last year during the pandemic and I wanted to visit the family and one of my cousins told me not to go because there were rumours that he died from Covid-19. So, for that reason, I could not visit his family and even my husband was telling me not to go because it is not safe. |
| **21.Has this had any impact on your use of MCH services during the pandemic? if yes, explain how**  These rumours from the community and from the media also contributed to my unwillingness to go to the health facility because they were advising people in the media not to go to the health facility as is it not safe. |
|  |
| **Institutional factors** |
|  |
| **24.Are you satisfied with the care provided by this health facility during the pandemic? probe: consultation time, treatment and respect from health workers. Has this stopped you from visiting health facilities?**  I was satisfied with service that I received because when you go to the health facility, they check your temperature which was never done before, and they introduced other measures such as cleaning the environment. As you know in public facilities, this was hardly done before the pandemic. so, in that sense the pandemic had a beneficial effect as it improved the quality of service. |
| **25.Do you think this health facility had adequate medical supplies during the pandemic? if no, give reasons. Probe- has this stopped from visiting health facilities.**  No, as you sometimes don’t have all the medicines you need. So, you have to buy them from private pharmacies. This, however, was not the reason I was not coming to health facilities because for me I even prefer to buy the medicines outside. |
|  |
| **27.What are your perceptions about the health workers in this facility? (e.g., competence or behaviour of health workers). probe- has this stopped you from visiting health facilities.**  I saw them advising people to observe social distancing and before you enter the hospital, you need to wear a face mask. So, as long as you abide by these rules, you don’t a problem with them. |
| **28.Do you think the health workers were following the Covid-19 precautionary measures appropriately? For example, were they always wearing face mask and PPEs? Probe-has this stopped from visiting health facilities?**  No, most of the health workers were not following the precautionary measures correctly. It is because of this that many people coming to the health facility refuse to wear face mask. This also contributed to my unwillingness to come to the health facility. I wasn’t coming because the health facility environment was not conducive at all which made me feel unsafe to come to the health facility, but as of now it is becoming normal. |
| **Policy factors** |
|  |
| **30.To prevent infection in health facilities, infection prevention and control measures, such as mandatory screening, wearing of facemask and social distancing, have been introduced in many health centers. What is the effect of these measures on your use of MCH services during the pandemic?** |
| The mandatory wearing if face mask was another reason why I was not willing to go to certain health facilities because I feel uncomfortable wearing it. So, that is why I wasn’t putting on a face mask, especially when I was pregnant. When I go to a health facility, if they ask me to put it on, I just tell them my situation. When I put it on, I feel like suffocating?  **32. What do you think is the effect of these measures on other people’s willingness to come for MCH services?**  Sometimes, I used to see people not coming to the health facility because they don’t have the money to buy a face mask. |
|  |
| **34. Was there any other barrier to accessing health care services during the pandemic that I did not ask you about?**  I don’t believe that Covid-19 is real because I have never seen with my own eyes a person who was infected with Covid-19 and died from it. I only hear it on the news.  **35. What do you think the government should do to prevent a decline in use of MCH services in the event of another pandemic?**  The government should continue sensitising people about the Covid-19 pandemic and encourage them to come to the health facilities. Also, as some health workers do not follow the precautionary measures and keep socialising, the government should do something about that. Because that was the main reason, I was not coming to the health facilities. Also, people had to put on a face mask when getting into the health facility, but some remove it as soon as they get in. So, the wearing of face mask was only enforced at the gate and was not enforced inside the hospital premises.  **36. What advice would you give to people who are not using MCH services during the pandemic?**  I would advise them to come and visit the health centre because if you are sick, there are other diseases that are more dangerous than Covid-19 that are escalating because of fear. So, they should really visit the health centre. |
