# Supplementary material for: Factors that influenced utilization of antenatal and immunization services in two local government areas in The Gambia during COVID-19: An interview-based qualitative study
Source: PLoS One. 2023 Jun 29;18(6):e0276357. doi: 10.1371/journal.pone.0276357 (PMC10309596; doi:10.1371/journal.pone.0276357)
Supplement: S1 File — (ZIP) [file pone.0276357.s001.zip › Supporting information / Health worker 2.docx]

In-depth interview questionnaire for health workers

**Introduction and Consent**

Hello, my name is Abdourahman Bah. I am a final year (MRC sponsored) BSc Global Health student at Queen Mary University of London. I am interviewing health workers and mothers in The Gambia to learn about the impacts of Covid-19-related lockdown measures on utilisation of mother and child services. The interview will take about 30 minutes. All the information I obtain will remain strictly confidential. You may choose not to answer any question that makes you feel uncomfortable.

Do you have any questions?

Do you agree to being interviewed? Yes

| **Background** |
| --- |
| 1. **Could you please tell me where you live?**   I live in Sukuta-nema-su |
| 1. **Please tell me for how long you have been working in this health facility**.   I have been working here for five years now. |
| 1. **What motivated you into pursuing a public health career?**   Like I said, during those days, when we were young, we only see a colleague or a senior member of the community. We were just trying to copy others, but to be honest with you, I never knew much about public health before joining as I was just motivated by others. It was when I joined there and during my first and second year when I really appreciated it. That is what is still keeping me in the profession if not I would have gone already. |
| 1. **What MCH services are provided in this facility? Probe: immunisation, antenatal care**   Let say, starting from antenatal care services, postnatal, and our side which is immunisation.   1. **Did the provision of these services continue during the pandemic?**   Yes, the provision of the essential ones continued, including immunisations. The only service that was interrupted was the weighting. That service is still been stopped. People were many and grouped together. It takes a lot of time, so we took it out to avoid that long waiting time. If you come and you don’t have it, you go home immediately. We don’t want people to stay here for long. That was basically the only service that was affected. Other than that, everything was okay. |
| 1. **Did the health facility stay open during the pandemic, and for how long?**   Yes, it was closed because we had some positive cases of Covid-19 here, but was only closed for fumigation and it wasn’t the entire hospital that was closed. It was only some departments that were closed. The closure time was also just for thirty minutes. It was less than a day. It was because of the chemical that people were asked to stay away. |
| 1. **Have you noticed any changes in utilisation of MCH services during the pandemic? For example, do you see fewer or more patients than usual?**   Yes, there was big change. So many people stayed way. I can tell you that there was about fifty percent reduction in the number of people coming for MCH services. This was because of fear of the pandemic. There was so much information out there, such as hospitals are not safe. It was all because of safety. You know how much protective women are of their children. So, they don’t want to expose them to the virus. Others would even tell you that health clinics has stopped. We don’t even know where they got that information. They would tell you that they have heard that the hospital has stopped offering immunisation services. so, for that reason, they were no longer taking their children for immunisation. They misunderstood the difference between the weighting and the actual immunisation. We told them that the weighting has been stopped, so they automatically thought that immunisation services was also included. So, that was also the communication lap. Most of them thought the whole service was stopped. Fear of the pandemic was also another factor. Sometimes you would be here and even one health worker can handle that clinic day, but they are now coming back. The service was greatly affected. All of our targets were down, but it is now going back to normal. |
|  |
| **Individual factors** |
| 1. **From the perspective of health workers, how safe do you think it is to provide MCH services during the pandemic?**   Basically, it was just difficult because grouping about fifty to hundred people together. Because trying to tell these women to observe social distancing was just a problem. It was just not that safe, but we tried to be so protective of our selves. We made sure that all of them put on face mask, and when we check your card, if your child doesn’t need to be immunised, we immediately asked you to go home. We didn’t allow them to waste time. After being immunised, we advised them to go home immediately. We also removed some of the chairs outside here so that there can be some space. we tried to make sure that they are far apart. Even though this was difficult to maintain, we tried out best. |
| 1. **How safe is for women to access MCH services in this facility at that period?**   Like I said, it was not safe. As you know, hospital settings are reservoir for infection, where you can get it at any time. Because this is where sick people are admitted whom you can easily get in contact with. We are using the same structure, the same equipment and the same entrance. So it wasn’t that safe but just tried as much as possible to educate them. |
| 1. **Did you or your colleagues work more or less hours during the lockdown? If yes, please explain why**?   Other offices/departments were doing rotations, but for the public health office, we didn’t do that. All of us come at once, so it was normal service delivery here from 8am to 4pm. Although the workload reduced a bit, we were always around. |
| **Interpersonal factors** |
| 1. **What is your family’s attitude in your provision of MCH services during the pandemic? (Are they supportive or not? If yes, explain how?**   You know in The Gambia, some don’t even belief that Covid-19 really exist. So, if they don’t belief that, then there is no way they will tell you not to go to work. So, I don’t even discuss that with my family. |
| 1. **Have you noticed any changes in your colleagues’ attitudes in providing MCH services during the pandemic? probe: did you experience a reduction in staff’s work appetite? If yes, explain why (maybe due to lack of risk allowance and patient overcrowding)**   No, there was no change, they were always ready to provide the service. Some of us were even going the extra mile to help others. About three of us here are also with the Covid-19 rapid response team. So most of us, we were going the extra mile.   1. **What incentives were provided by the government to motivate health workers during the pandemic?**   There was a time when we were provided with motor bikes, fuel and allowance. |
| 1. **What is your attitude towards MCH service users during the pandemic? probe: were they making your work easier or more difficult?**   It was very difficult to convince others. Even if you tell a woman to go out and buy a mask, they will tell you that they don’t have money to buy a mask. So, we sometimes had to compromise so that we can agree at a point. This is because we don’t want the child to go home and missed their immunisation. Also, the service coverage was already going down, so that could be a missed opportunity for the child, as some women would not come back. So, sometimes we provide mask for her and if we can’t, we asked for another woman’s help to bring the child inside. Some were following all of our instructions, but as you know there are some crazy ones. They think everything is politically motivated. They think people are doing this to get money. They say so many abusive words, but that comes with the job. |
| **Community factors** |
| 1. **Have you experienced any changes in people’s perception in the community about the use of MCH services during the pandemic? if yes, explain.**   In my area, we don’t have many women of childbearing age and for me personally, I am mostly indoors. I don’t go out during that Covid-19 time. Also, we I am living and where I work are two different settings. So, if you go to Sukuta health centre I may not know. |
| 1. **Have you experienced any challenges in providing MCH services due to transport difficulties? if yes, explain how**   I didn’t experience any transport difficulties during the pandemic as I was having a motor bike. Transport could have been a big problem without the motor bike because most the drivers were sitting down due to strike. People also had to pay double fare and some drivers refused to go to work. |
| **Institutional factors** |
|  |
| 1. **What do you think of the quality of care provided by this health facility during the pandemic?**   The quality of service was not affected. The workload was reduced but the quality of service was not affected. |
| 1. **Do you think this health facility had adequate medical supplies during the pandemic? if no, give reasons**.   There was a stock out of the Polio vaccine. The issue was with the supply chain which was affected by the pandemic. We stopped giving it for about three to four months.   1. **Do you think this health facility had adequate PPEs during the pandemic? if no, give reasons. Did that have any effect on your willingness or ability to provide MCH services?**   Sometimes it was adequate while sometimes it was scarce. We had to the work regardless of the availability of PPEs, but mostly we always improvise. For example, if we don’t have the medical face mask, we use the local face mask made with cloth. The infection control staff used to go around to make sure that every health worker has face mask. |
| 1. **Do you think this facility had enough manpower to provide MCH services during the pandemic? if no, give reasons**   In our unit, we were not that much affected. We didn’t have any case in our unit. It was only one positive case that we had but even that was before it was detected. Before the sample result was received it almost about a week. The fourteen days had almost collapsed, so had been coming to work. He only stayed home for about four to five days.   1. **What do you think of the health facility environment? Probe: is the facility clean and not overcrowded?**   The health environment was conducive. Even the number of people coming for antennal care was reduced. |
| **Policy factors** |
|  |
| 1. **To prevent infection in health facilities, infection prevention and control measures, such as mandatory screening, wearing of PPEs and face mask, have been introduced in many health centers. What is the effect of these practices on provision of MCH services?**   Sometimes to feel that you are working in a safe environment is something very important. That a feeling that somebody is doing their job very well. |
| 1. **What is the effect of these measures on utilisation of MCH services during the pandemic?**   The introduction of these measures discouraged a lot of women from coming for MCH services. others would even tell you that paying twenty-five dalasi for a face mask is expensive. Even you the health worker has that push and pull with them; they may tend to stay away from the health facility for the subsequent month. I know this is also a factor as they are not provided with face mask for free. For some we do understand that they may have some respiratory problems. so, we rushed them in so that they can receive the service quicky, but they are some who would not explain their condition to us, so we would not understand their condition. |
| 1. **Are there any other factors that may have negatively impacted your ability to provide MCH services during the pandemic that I haven’t asked you about? if yes, please state them and explain how?**   The main barrier I can say was communication because there was a time, when they would not even trust the childhood vaccines, we were given them here. The perception they had was that we were giving them Covid-19. The believed that the Covid-19 virus was included in the childhood vaccines. There was so much information out there.   1. **Are there any other factors that may have contributed to the decline in the use of MCH services during the pandemic that I haven’t asked you about? If yes, please state them.**   I think it was mostly the miscommunication that we had with them and the fear of Covid-19. The name of Covid-19 was just too much for some people. Also, for most people they were just following the stay-at-home policies introduced by the government. For some people, they also have the perception that hospitals are not safe, so they rarely come to the health facility. Also, the media is another factor as they usually come to the health facility to make a video when something happens here. For that reason, people tend to shy away. I can tell you the first death we had here, that was the guy from Bangladesh. After that, before you see somebody enter the hospital was a problem. So, people were saying there was a positive case in BMCHH. So, for that reason, people tend to stay away.   1. **Kindly explain some of the coping/adaptive mechanisms that has helped you while providing MCH services.**   We had that rigorous health education. When they come, we talked to them and gave them some important messages. So, it was just sensitisation.   1. **To prevent the decline in use and provision of MCH services in the event of another pandemic or second wave, what do you think the government should do?**   I think basically it should the same thing which is sensitisation to make sure that they are aware because awareness was the problem.   1. **What advice would you give to people who are not using MCH services during the pandemic?**   My advice to them is to come for MCH service. Even though it is not that safe at hospital but even at home you can have Covid-19. So, there is no way you can run away from it. You can only protect yourself. I would just advise them to continue coming and not to spend much time here and put on the PPEs. |
