# Supplementary material for: Factors that influenced utilization of antenatal and immunization services in two local government areas in The Gambia during COVID-19: An interview-based qualitative study
Source: PLoS One. 2023 Jun 29;18(6):e0276357. doi: 10.1371/journal.pone.0276357 (PMC10309596; doi:10.1371/journal.pone.0276357)
Supplement: S1 File — (ZIP) [file pone.0276357.s001.zip › Supporting information /Respondent 2.docx]

In-depth Interview Questionnaire for MCH service Users

**Introduction and Consent**

Hello, my name is Abdourahman Bah. I am a final year (MRC sponsored) BSc Global Health student at Queen Mary University of London. I am interviewing health workers and mothers in The Gambia to learn about the impacts of Covid-19-related lockdown measures on utilisation of mother and child services. The interview will take about 30 minutes. All the information I obtain will remain strictly confidential. You may choose not to answer any question that makes you feel uncomfortable.

Do you have any questions?

Do you agree to being interviewed? Yes

| **Background** |
| --- |
| 1. **Could you please tell me where you live – Probe: house of residence is?**   I live in Bundung |
| 1. **Please tell me how you got here today? Probe: public transport, private or walked.**   I walked from my home to the health facility.   1. **Have you gone to the health facility during the COVID-19? Were you treated differently? Were there any different procedures? If so, what are they doing that is different?**   No, I was not going to any health facility during the pandemic last year. I was not taking my child for immunization to the health facility during the pandemic last year. I started bringing her for immunization last month (July). |
| 1. **Have you changed the way you access this service during the outbreak? If so, how? If you have changed, are you going more times or less times and if so, what are the reasons? Probe-economic? Fears?**   I was not taking my child for immunization to the health facility during the pandemic last year. I started bringing her for immunization last month (July). I used to take her to a hospital in Banjul before the Covid-19 pandemic, but when the pandemic started, they stopped us from going there. This was because of the pandemic. So, they told us not to take our children there because of the pandemic, but they later called us and told us to bring our children when they are supposed to be immunized. So, whenever it is time for my child to be immunized, they would give me a call. Stopped taking her immunization to the hospital in February 2020, but they later called me to bring me my child for immunization. There were many months that my child was not immunized because even I took her to the hospital, they would ask me to go home. Also, they stopped weighing children and they were only doing the immunization. |
|  |
| **Individual factors** |
| 1. **How safe do you think it is to access MCH services during the pandemic? - Probe: have these concerns stopped you from using these health facilities?**   For me, it is safe because if you have a child, you must take the child to the health facility. At that time, if you are breastfeeding, they would advise you to wash your breast first before it to your child. For me, fear of getting infected at the health facility did not stop me from going to the health facility. Whenever, I feel like I understand my body, I go to the health facility and when my child is not feeling well, I also take her to the health facility. |
| 1. **Have you experienced any financial difficulties (e.g., transport costs) in accessing MCH services during the pandemic? if yes, explain. Probe- have these difficulties stopped you from using these health facilities?**   Off course, I had transport difficulties at that time because at that time, I was staying in Bundung. I used to pay about 50 dalasi to get to Banjul. When coming back, I used to face the same problem. Transport at that time was very costly and there were not many vehicles. Their decision to stop us from going to the health facility was huge a favour to me, because I used to spend lot of money on transport. When we get there, we also wait for a long period of time. I don’t think that was because of the pandemic; I think that is normal here because we had to wait for the health workers while they have their breakfast. That is why when I go there, I always quarrel with them because you come from a far place. Instead of them attending to you, they will be sitting and chatting with their colleagues and neglecting you. |
| **Interpersonal factors** |
|  |
| **19.Have you noticed any changes in your friends’ attitudes in use of MCH services during the pandemic? probe: are they going more times or less times?**  Yes, I have seen many of them not going to the health facility during the pandemic. The reason they would give is that if they go the health facility, they would be told that they have Covid-19. So, for that reason, they are afraid of going to the health facility. For some of them, when they fall sick, they preferred to use traditional medicine than going to the health facility. Others would also go to the pharmacy, but for me, I prefer going to the health facility where I will be checked because if you go to the pharmacy, they will not check you, they will only give you medicine to take |
| **Community factors** |
| **20.Have you noticed any changes in people’s perception in your community about the use of MCH services during the pandemic? if yes, explain. Probe: give examples of people being afraid of visiting facilities due to stigma associated with visiting health facilities or fear of being quarantined etc.**  Some people were not going to the health facility because they were afraid of getting infected at the health facility. You know when there are many rumours circulating around, people feel scared to go to the health facility, but for me, this did not prevent me from going to the health facility. |
|  |
|  |
| **Institutional factors** |
| **23.Did the health facilities stay open during the pandemic? if no, state how this may have affected your access to MCH services.**  Before the pandemic, I used to go to Hagan Hospital in Banjul, but when the Covid-19 pandemic started, they closed the health facility. I brought my child there for immunisation, but they said they have closed the hospital and they are not seeing patients. I went there several times, but they told me that it was closed. So, I went home and stopped taking my child for immunisation. Several months later, they called me and told me that I need to take my child for immunisation. That was when I re-started taking my child for immunisation. |
| **26.Do you think this facility had enough manpower to provide MCH services during the pandemic? if no, give reasons**  During that time, there were very few health workers and some of those who were there would not even attend to patients. They would be sitting and chatting with their friends while leaving the patients not being attended to. |
| **27.What are your perceptions about the health workers in this facility? (e.g., competence or behaviour of health workers). probe- has this stopped you from visiting health facilities.**  I always have problems with the health workers because whenever I see them doing something wrong, I say it straight to them. The pandemic made things very difficult because there were not even enough medicines at the health facilities. You would go to the health facility, and they would ask you to go and buy the medicines from the private pharmacies. People suffered a lot during that period, as people were not having money at that time. even me, I experienced that same problem. There was a time when my child sick and I took him to Banjul Hospital, but I was told that there was no medicine no available. I took her there several times, but I was told there was still no medicine available. So, I decided to take her to the MRC@LSHTM. |
| **28.Do you think the health workers were following the Covid-19 precautionary measures appropriately? For example, were they always wearing face mask and PPEs? Probe-has this stopped from visiting health facilities?**  Some of them were following the precautionary measures correctly, but some were not. It was plenty of them that were not following the precautionary measures, such not wearing of face mask and that is not safe because they are the ones treating people and advising people to follow the precautionary measures, so they should also apply those measures. I once went to Banjul Hospital and one of the health workers asked me to wear a face mask while she was wearing one herself, so I pointed the same thing to her, and she got angry with me. They shouldn’t be telling me people to follow the precautionary measures while they are not following them, themselves. They should be the ones to wear it first. This may even prevent other women from taking their children to immunisation such hospitals as they would feel unsafe since the health workers are not following the precautionary measures. |
| **Policy factors** |
|  |
| **30.To prevent infection in health facilities, infection prevention and control measures, such as mandatory screening, wearing of facemask and social distancing, have been introduced in many health centers. What do you think of the implementation of these measures in the health facilities? Probe: were they implemented correctly?**  Before you enter the health facility, you have to wash your hands and also have to wear a face mask, but those were telling us to put on a face mask, you would find them not wearing a face mask. |
| **31.What is the effect of these measures on your use of MCH services during the pandemic?**  For those who have breathing problems, the mandatory wearing of face mask could be a problem for them. The mandatory wearing of face mask was also a reason why some women were not bringing their children for immunisation. I have seen this with my own eyes. I came here the other day to give blood to my sister, but I did not bring a face mask, so I was not allowed to get into the hospital. I had to go and buy a face mask. I may have the money to buy a face mask, but other people may not have that money and since the hospital does not provide free mask for those who cannot buy one, there only option would be to go home. |
|  |
| **35. What do you think the government should do to prevent a decline in use of MCH services in the event of another pandemic?**  The government should help us by providing enough medical supplies. Some mothers are not willing to take their children to the health facility because they know that they will not have the medicines they need. So, to encourage mothers to go to health facilities, they should make sure that the medicines needed are always available. The health workers should also respect their patients. If people come to the health facilities, they should be given the service in a nice manner. They should be neglecting their patients by just sitting and chatting in groups. They are not paid to chat; they are paid to attend to patients. They should also follow the precautionary measures, such as wearing of face mask.  **36. What advice would you give to people who are not using MCH services during the pandemic?**  Not bringing your child for immunisation is not good. If your child is sick, you should also take them to the health facility rather than taking them to the pharmacy where there are not checked. My advice to them would be to take their children to the health facility, so that their children can get immunised |
