# Supplementary material for: Factors that influenced utilization of antenatal and immunization services in two local government areas in The Gambia during COVID-19: An interview-based qualitative study
Source: PLoS One. 2023 Jun 29;18(6):e0276357. doi: 10.1371/journal.pone.0276357 (PMC10309596; doi:10.1371/journal.pone.0276357)
Supplement: S1 File — (ZIP) [file pone.0276357.s001.zip › Supporting information /Respondent 16.docx]

In-depth Interview Questionnaire for MCH service Users

**Introduction and Consent**

Hello, my name is Abdourahman Bah. I am a final year (MRC sponsored) BSc Global Health student at Queen Mary University of London. I am interviewing health workers and mothers in The Gambia to learn about the impacts of Covid-19-related lockdown measures on utilisation of mother and child services. The interview will take about 30 minutes. All the information I obtain will remain strictly confidential. You may choose not to answer any question that makes you feel uncomfortable.

Do you have any questions?

Do you agree to being interviewed? Yes

| **A** | **Background** | |
| --- | --- | --- |
| 1 | Could you please tell me where you live – Probe: house of residence is? | I live in Bakoteh |
| 2 | Please tell me how you got here today? Probe: public transport, private or walked. | I used public transport |
| 3 | Have you used MCH services during the pandemic? Probe: immunisation, antenatal consultations etc. | During the pandemic, I used antenatal care services, as I was pregnant at that time. |
| 4 | If yes, what MCH Service have you used during the pandemic? Kindly state the reasons. |  |
| 5 | Have you changed the way you access this service during the outbreak? If so, how? If you have changed, are you going more times or less times? | Normally, I used to come every month. |
| 6 | Kindly mention the number of times you accessed this service in the last twelve months. | I started coming for antenatal care in August last year. I delivered in December. |
| **B** | **Individual factors** | |
| 7 | How safe do you think it is to access MCH services during the pandemic? - Probe: have these concerns stopped you from using these health facilities? | I felt it is not safe, but I had to protect myself by wearing face mask and applying hand sanitizer. |
| 8 | Have you experienced any financial difficulties (e.g., transport costs) in accessing MCH services during the pandemic? if yes, explain. | No, in contrast to others paying a double fare, I used to pay a single fare. The difficulty I experienced was the fact that it was difficult for me to obtain a vehicle when I need one. |
| **C** | **Interpersonal factors** | |
| 9 | What is your family’s attitude, including your husband, in your use of MCH services during the pandemic? Probe: Do they encourage or discourage you? In what way? | My husband was supportive when he around. However, he was only here until August and then he left for the US. That’s when I started coming this health facility. He never stopped me from coming. |
| 10 | Have you noticed any changes in your friends’ attitudes in use of MCH services during the pandemic? | I did not have any experience of that. |
| **D** | **Community factors** | |
| 11 | Have you noticed any changes in people’s perception in your community about the use of MCH services during the pandemic? if yes, explain. Probe: give examples of people being afraid of visiting facilities due to stigma associated with visiting health facilities or fear of being quarantined etc. | Yes, I used to hear from people sometimes. They used to say the hospital is not safe during this pandemic. These some of the things some people used to say. |
| 12 | Has this had any impact on your use of MCH services during the pandemic? if yes, explain how | It did not affect my coming for MCH services during the pandemic. |
| 13 | Have you experienced any challenges on getting to health facilities during the pandemic? if yes, state them (e.g., lack of transport) |  |
| **E** | **Institutional factors** | |
| 14 | Did the health facilities stay open during the pandemic? if no, state how this may have affected your access to MCH services. | Normally they would give us appointment and they would be opened every time. The health facility was available whenever we had an appointment. |
| 15 | Are you satisfied with the care provided by this health facility during the pandemic? probe: consultation time, treatment and respect from health workers. | Our major problem here is that the waiting time here was very long at the time of the pandemic. so, we would have to wait long before can see a health worker. The waiting time was exacerbated during the pandemic |
| 16 | Do you think this health facility has adequate medical supplies during the pandemic? if no, give reasons. | Yes, there was enough medical supplies during the pandemic. We did not experience any shortage of medical supplies, as we would get the medicines prescribed. |
| 17 | Do you think this facility has enough manpower to provide MCH services during the pandemic? if no, give reasons | There were enough health workers, but the problem was that the hospital was over-crowded. The overcrowding made me feel unsafe, but I just had to protect myself |
| 18 | What are your perceptions about the health workers in this facility? (e.g., competence or behaviour of health workers) | I was comfortable with the way the health workers treated me. |
| 19 | How safe do you think it is for women to access MCH services during the pandemic in this health facility? Please explain. | I don’t think it was safe to come for MCH services during the pandemic because you can easily get infected. Even if I take all precautionary measures, I still don’t feel comfortable coming here to health facilities during the pandemic. I only came because it was compulsory. |
| **F** | **Policy factors** | |
| 20 | Did the lockdown measures, such as stay at home policies, travel bans, etc, put in place last year had any impact on your use of MCH services during the pandemic? if yes, explain how. |  |
| 21 | To prevent infection in health facilities, infection prevention and control measures, such as mandatory screening and wearing of facemask, have been introduced in many health centers. What is the effect of these practices on waiting time and quality of service? |  |
| 22 | Have these measures had any impact on your use of MCH services during the pandemic? if yes, explain how | These measures did not have any effect on my coming to health facilities during the pandemic. this is because I know that they are put in place to protect us. |
| 23 | Are there any other measures introduced either in your community or health facilities that have had an impact on your use of MCH services during the pandemic? (e.g., policy to close certain health facilities or scale back MCH service provision) if yes, please state them and explain how. | I did not experience any of these, as I visited only this hospital during the pandemic. this one was always open. |
| 24 | Was there any other barrier to accessing health care services during the pandemic that I did not ask you about? | The only difficulty I experienced was the fact that I had to stay here for a very long time before I could access the service I needed. |
