# Supplementary material for: Factors that influenced utilization of antenatal and immunization services in two local government areas in The Gambia during COVID-19: An interview-based qualitative study
Source: PLoS One. 2023 Jun 29;18(6):e0276357. doi: 10.1371/journal.pone.0276357 (PMC10309596; doi:10.1371/journal.pone.0276357)
Supplement: S1 File — (ZIP) [file pone.0276357.s001.zip › Supporting information /Respondent 17.docx]

In-depth Interview Questionnaire for MCH service Users

**Introduction and Consent**

Hello, my name is Abdourahman Bah. I am a final year (MRC sponsored) BSc Global Health student at Queen Mary University of London. I am interviewing health workers and mothers in The Gambia to learn about the impacts of Covid-19-related lockdown measures on utilisation of mother and child services. The interview will take about 30 minutes. All the information I obtain will remain strictly confidential. You may choose not to answer any question that makes you feel uncomfortable.

Do you have any questions?

Do you agree to being interviewed? Yes

| **A** | **Background** | |
| --- | --- | --- |
| 1 | Could you please tell me where you live – Probe: house of residence is? | I live in Jabang |
| 2 | Please tell me how you got here today? Probe: public transport, private or walked. | My husband brought me in his vehicle. |
| 3 | Have you used MCH services during the pandemic? Probe: immunisation, antenatal consultations etc. | Yes, I used to come for antenatal care, but then started bringing my child for immunisation. |
| 4 | If yes, what MCH Service have you used during the pandemic? Kindly state the reasons. |  |
| 5 | Have you changed the way you access this service during the outbreak? If so, how? If you have changed, are you going more times or less times? | I continued bringing my child for immunisation during the pandemic. It is only when I was ill or travelled when I didn’t bring my child for immunisation. I bring my child for immunisation for the child’s welfare. During the peak of the Covid-19 pandemic, I was pregnant, but that didn’t prevent me from coming for antenatal care services. At that time, I was going to Serrekunda health centre. At that time, we used to have many difficulties there. If you don’t have a mask, they would not allow you to enter. Thank God, few months later my husband got a promotion and then I started coming to this health facility in September 2020. I started coming here on a Thursday and I delivered my child on the following Sunday. When I was going to Serrekunda health centre, I was disturbed a lot by the health workers, but that did not stop me from going for antenatal care. |
| 6 | Kindly mention the number of times you accessed this service in the last twelve months. |  |
| **B** | **Individual factors** | |
| 7 | How safe do you think it is to access MCH services during the pandemic? - Probe: have these concerns stopped you from using these health facilities? | When I would go the health facility, I would be told to wear a face mask and wash my hand thoroughly. So, when I get there that is what I would do: apply a hand sanitiser and wear a face mask. So, when the health workers come, I give them my card but sometimes they would even limit the number of people they would allow in a day. They would allow only around twenty to twenty-five people a day. So, if you don’t go early, you will not be allowed in. The Serrekunda health facility didn’t have a scanner, so I used to go to Babun Fatty for the scan |
| 8 | Have you experienced any financial difficulties (e.g., transport costs) in accessing MCH services during the pandemic? if yes, explain. |  |
| **C** | **Interpersonal factors** | |
| 9 | What is your family’s attitude, including your husband, in your use of MCH services during the pandemic? Probe: Do they encourage or discourage you? In what way? | Because my husband is educated, he does not take the importance of good health lightly. He would not want me not to come for MCH services during the pandemic. He would take me on his personal vehicle to the health facility. He would leave me there and his driver would pick me up when I am ready at the health facility. |
| 10 | Have you noticed any changes in your friends’ attitudes in use of MCH services during the pandemic? |  |
| **D** | **Community factors** | |
| 11 | Have you noticed any changes in people’s perception in your community about the use of MCH services during the pandemic? if yes, explain. Probe: give examples of people being afraid of visiting facilities due to stigma associated with visiting health facilities or fear of being quarantined etc. | There was a time, when your child doesn’t need to be immunised, you will be told not go for weighing your child. That’s why I even stopped taking my older child for immunisation. |
| 12 | Has this had any impact on your use of MCH services during the pandemic? if yes, explain how |  |
| 13 | Have you experienced any challenges on getting to health facilities during the pandemic? if yes, state them (e.g., lack of transport) | I didn’t experience transport difficulties during the pandemic because my husband would take me on his personal vehicle to the health facility. He would live me there and his driver would pick me up when I am ready at the health facility. However, if you don’t have a vehicle and have to use public transport, you have a big problem because fares are very high . |
| **E** | **Institutional factors** | |
| 14 | Did the health facilities stay open during the pandemic? if no, state how this may have affected your access to MCH services. | At the Serrekunda health centre, there was an MRC department that was closed at the peak of the pandemic. I used to bring my child there, but there was a time when I was told it is currently closed because of the pandemic. That is why I stopped going there. |
| 15 | How satisfied are you with the care provided by this health facility during the pandemic? probe: consultation time, treatment and respect from health workers. | For me, I mostly go to private health facilities. Apart from this health facility, I used to go to another German health facility. The private health facilities I visited didn’t have any problems. That’s why everyone is trying to go to private health facilities. If you go to public health facilities, you will experience many difficulties. |
| 16 | Do you think this health facility has adequate medical supplies during the pandemic? if no, give reasons. | At the Serrekunda health facility, there was a shortage of essential medicines. That is even one of the reasons why some people refuse to go to health facilities. When you go there, you would be given a prescription to buy the medicines at the pharmacy. That is also why some people would just visit a pharmacy without going to a health facility. When my husband did have much money, I used to go to there, but it wasn’t easy. |
| 17 | Do you think this facility has enough manpower to provide MCH services during the pandemic? if no, give reasons |  |
| 18 | What are your perceptions about the health workers in this facility? (e.g., competence or behaviour of health workers) | At the Serrekunda health facility, there was a very bad Manjago health worker. He used to disturb us a lot. His behaviour and other health workers could prevent others from coming for MCH services at that health facility. For me, no matter how difficult you are, you will hardly fine anything to complain about me. I know what I come for, which is for antenatal service or for immunisation service. I just sit at my end and mind my own business. However, if you try to start any trouble with me and I will respond in full. We are all Gambians. No one should disturb anyone. we should respect each other. In my opinion, this kind of behaviour is a result of the Covid-19 pandemic. this is because during the pandemic, when the health facility gets overcrowded, the health workers feel overwhelmed. |
| 19 | How safe do you think it is for women to access MCH services during the pandemic in this health facility? Please explain. | If you follow all the precautions which include washing your hands and wearing face mask, you will be fine. However, some people don’t like wearing face mask. They would say wearing face mask would make it difficult for them to breath. Nonetheless, in private health facilities, you will not be allowed to enter without a face mask. Even in Serrekunda health centre, if you don’t have a face mask you will be denied entry. For this reason, some people refuse to go to health facilities. This is because they can’t put on a face mask whenever they go to the health facility. |
| **F** | **Policy factors** | |
| 20 | Did the lockdown measures, such as stay at home policies, travel bans, etc, put in place last year had any impact on your use of MCH services during the pandemic? if yes, explain how. |  |
| 21 | To prevent infection in health facilities, infection prevention and control measures, such as mandatory screening and wearing of facemask, have been introduced in many health centers. What is the effect of these practices on waiting time and quality of service? |  |
| 22 | Have these measures had any impact on your use of MCH services during the pandemic? if yes, explain how |  |
| 23 | Are there any other measures introduced either in your community or health facilities that have had an impact on your use of MCH services during the pandemic? (e.g., policy to close certain health facilities or scale back MCH service provision) if yes, please state them and explain how. |  |
| 24 | Was there any other barrier to accessing health care services during the pandemic that I did not ask you about? | I delivered during the peak of the pandemic, which was in September 2020. There was a limit to the number of people who attended the naming ceremony. So, not much was done for the naming ceremony. |
