# Supplementary material for: Factors that influenced utilization of antenatal and immunization services in two local government areas in The Gambia during COVID-19: An interview-based qualitative study
Source: PLoS One. 2023 Jun 29;18(6):e0276357. doi: 10.1371/journal.pone.0276357 (PMC10309596; doi:10.1371/journal.pone.0276357)
Supplement: S1 File — (ZIP) [file pone.0276357.s001.zip › Supporting information /Health worker 14.docx]

In-depth interview questionnaire for health workers

**Introduction and Consent**

Hello, my name is Abdourahman Bah. I am a final year (MRC sponsored) BSc Global Health student at Queen Mary University of London. I am interviewing health workers and mothers in The Gambia to learn about the impacts of Covid-19-related lockdown measures on utilisation of mother and child services. The interview will take about 30 minutes. All the information I obtain will remain strictly confidential. You may choose not to answer any question that makes you feel uncomfortable.

Do you have any questions?

Do you agree to being interviewed? Yes

| **Background** |
| --- |
| 1. **Could you please tell me where you live?**   I live in Banjulunding |
| 1. **Please tell me for how long you have been working in this health facility**?   I am in my fifth year now. I work here for part-time. I come here only on Saturdays. |
| 1. **What motivated you into pursuing a public health career?**   Actually, it is a result of conditions that I feel can be prevented because I believe most of the conditions happening are a result of lack of understanding. But actually, when you are equipped with knowledge you are able to change certain scenarios happening in your proximity or within your own community. |
| 1. **What MCH services are provided in this facility? Probe: immunisation, antenatal care**   Immunisation, antenatal, deliveries and then post-delivery services. |
| 1. **Did the health facility stay open during the pandemic, and for how long?**   It was open all along until and unless they had a confirmed case of covid-19, when the place was fumigated for three days, and work was ceased during these days. However, MCH services were not affected since it did not fall on the days MCH services were provided. Some services were shifted to the following week, and we were able to provide immunisation services. |
| 1. **Have you noticed any changes in utilisation of MCH services during the pandemic? For example, do you see fewer or more patients than usual?**   Yes, there was a reduction in the inflow and then the majority of MCH users when we called, they were telling us that because of the pandemic our family members are not allowing us to move out, or going to health facilities, where it was always crowded. To encourage people to come, we had to had discussion with them and explained to them the impact of the Covid-19 and the impact it would have on vaccine preventable diseases, which is worse than that of the pandemic. Majority adhered and reasoned with us, but during the peak of the pandemic, there was a massive reduction in the number of clients coming of MCH services. |
|  |
| **Individual factors** |
| 1. **From the perspective of health workers, how safe do you think it is to provide MCH services during the pandemic?**   Actually, we were going through all the protocols that were introduced, such as maintaining that hand hygiene whenever you come in contact with a patient and when you step into the hospital. Also, the majority of us were wearing face mask and all the clients had to put on a mask before coming into the health facility. However, due to the small space in the hospital, we could not maintain that 2 metre distance. Nonetheless, the women were so cautious that they always tried to keep some distance and cough hygiene was another thing that we advocated whenever you cough by coughing on you elbow or wearing a mask when you sneeze. |
|  |
| 1. **Did you or your colleagues work more or less hours during the lockdown? If yes, please explain why?**   At the facility level, there was this roster which resulted in massive reduction in staff coming in. you would come for two days and stay off for three days. So not all of us were coming at the same time, particularly during the peak of the pandemic. |
| **Interpersonal factors** |
| 1. **What is your family’s attitude in your provision of MCH services during the pandemic? (Are they supportive or not? If yes, explain how?**   My family was supportive. They know my role and responsibility. The pandemic came at a time when they need both of us. That is, both family members and health workers. They know that I was working under the preventive aspect, so I would be in the front line of anything that was going to happen. They were supportive and always concern when I am away. They would always ask, how are you doing there and how are the patients. The communication was thick and fast because I was part of even part of the covid-19 response team, so I work from Monday to Sunday. I had no time and would be at home late night, when I would be called to collect samples and coordinate the team to collect the samples. |
| 1. **Have you noticed any changes in your colleagues’ attitudes in providing MCH services during the pandemic? probe: did you experience a reduction in staff’s work appetite? If yes, explain why (maybe due to lack of risk allowance and patient overcrowding)**   Well, you know it is Gambia. The willingness is always there, but the motivation is never there. It not about the monetary aspect but is mostly about the protective that you need not being readily available. We were just sacrificing ourselves for the love of the country and the passion for the work we do. Other than that, we were even thinking of quieting because at that point in time seeing your colleagues contracting the disease and the ministry doesn’t even care if they were there or not. Feeding was another problem as well, as you know if you get the disease, you need supplements and if you are from a poor background, wherein you are the breadwinner, it is always traumatising. These were some of the reasons why some people were reluctant to provide MCH services. nonetheless, the provision of MCH services continued and everybody was willing to step up. |
| 1. **What is your attitude towards MCH service users during the pandemic? probe: were they making your work easier or more difficult?**   With regard to the Covid-19 pandemic, in the private health facility every worked fine. This is because they had there set rules which everyone had to follow. So, basically everything went fine. |
| **Community factors** |
| 1. **Have you experienced any changes in people’s perception in the community about the use of MCH services during the pandemic? if yes, explain.**   No, because in my community in Banjulunding, we developed a Covid-19 response team. Youths of the community bought some sanitary materials, which were distributed to the community members. We also did community health education about how to prevent yourself and how you can go about doing your day-to-day activities without getting infected with Covid-19. This is because the majority of us leading the team are health workers residing in the community. We saw it befitting to give back to our community. We did not give them money but equipped them with knowledge. It was so helpful. |
| 1. **Have you experienced any challenges in providing MCH services due to transport difficulties? if yes, explain how**   Yes, sometimes especially during the peak of the pandemic. there were so many misconceptions out there among vehicle owners and clients. For that reason, you would sometimes spend more than an hour looking for a vehicle, and you would sometimes have to pay five-times the usual fare. For example, instead of paying twenty dalasi, you may have to pay fifty dalasi or hundred dalasi. This transport difficulty had an impact on health workers willingness to travel to health facilities. Thinking of your salary and the fare you pay to get to the health facility. If you are paying a fifty-dalasi fare and if you are working Monday to Friday, that means you are spending about two hundred and fifty dalasi a week. So, fifty-dalasi times four, is thousand dalasi a month. So, if you take that out of your salary, you will have very little left. For that reason, you would sometimes here people saying, I will not go to work today because I did not have a vehicle. That is just the excuse they give, but the monetary aspect comes into play. When you have to give out fish money and you only have maybe around ten or twenty dalasi on you, so it becomes very stressful. |
| **Institutional factors** |
| 1. **Do you think there was adequate health facilities to provide MCH services during the pandemic? if no, state reasons**   Yes, there were adequate health facilities, but when the pandemic hit the health facilities, it became very hectic. Other facilities would be overwhelmed with clients. Proximity counts a lot. For instance, people from Yundum and Busumbala, instead of them going to Yundum, they would go to Banjulunding. But restrictions were put in place and health facilities on day-to-day basis would provide only antenatal services. As a result, so many things happened. You know these maternal deaths and abortions came up because they did not get the services they needed at the stipulated time. Some would be booked after four months and others after six months because of the pandemic. this is also because for you to been seen at the health facility, you would need to come to the health facility early in the morning. In fact, some would even sleep at the health facility overnight because only around twenty patients were seen a day. So, the pandemic had a very negative effect on women who were coming in for MCH services. you would hear them describing the trauma they experienced at that time and the bad experience they had during their pregnancy period. The provision of antennal care was badly affected, but immunisation services remained unaffected. |
| 1. **What do you think of the quality of care provided by this health facility during the pandemic?**   The provision of service during the pandemic was not satisfactory but was fair enough considering the situation and the risk associated with it. |
| 1. **Do you think this health facility had adequate medical supplies during the pandemic? if no, give reasons.**   That one was not available. Availability and utility should go together but none of those was there. Even gloves, it came to a time when we’re struggling to get them. We had to called donors to provide them for health workers. During that time the supply chain was never there but when the Covid-19 came it became worse. So, PPEs were not available. You know the attitude of health workers. some would say since I don’t have PPEs, I am not going to render the service. It is only few people, who had passion and believe in all mighty and take the necessary precautions, those were the ones who were sacrificing, but nothing was readily available for us to use during that peak period of the pandemic. |
| 1. **Do you think this facility had enough manpower to provide MCH services during the pandemic? if no, give reasons**   Looking at the WHO guideline, the manpower was not there even before the pandemic. this is because under the WHO guideline, they are a number of patients that a doctor should see, but in the Gambia, you don’t have that. One doctor can see even five thousand people in a month. The provision of service was disrupted because we have an enough number of health workers. It is just that we had this step down, wherein others would come in today and tomorrow and others would come the following day. The step down was done so as to reduce the transmission chain. It is also because there is a tiny space in the health facilities to accommodate many health workers and clients at the same time. There is also poor ventilation in many health facilities. We therefore saw it befitting to do a step down so as to reduce the spread of the disease. |
| **Policy factors** |
| 1. **Did the lockdown measures, such as curfews and stay at home policies etc., put in place last year had any impact on your use of MCH services during the pandemic? if yes, explain how.**   The lockdown and curfews introduced last year did have much impact on the use of MCH services. it is mostly the misconceptions about the disease that affected the use of services. People were misinformed and communication was broken from the beginning. This was because of the attitude of the government, as the government was telling people to socialise while the government was organising political rallies. This made the people lose trust in the government and stopped following government advice. |
| 1. **To prevent infection in health facilities, infection prevention and control measures, such as mandatory screening, wearing of PPEs and face mask, have been introduced in many health centers. What is the effect of these practices on provision of MCH services?**   Yes, it did have an effect on provision of MCH services. they said people should not gather but provision of MCH service requires people to gather. Looking at the little space available in many facilities, it was nearly impossible to practice social distancing. This contributed to people not going to health facilities since it is a gathering |
| 1. **What is the effect of these measures on utilisation of MCH services during the pandemic?**   Yes, it did as many were reluctant do that do that. These precautionary measures were not even adhered to in many of the facilities. What they needed was not available. It was in private facilities that these measures were adhered to. In public facilities, a poor individual thinking of fare to get to the health facility, if you say that person that they need to buy a facemask to get in. in fact, when people knew that every individual needs a mask, they increased the price of a mask. For instance, a mask use to cost ten dalasi and it was increased to twenty-five dalasi, thirty-five and fifty dalasi. So that also contributed to the lack of usage of PPEs. |
| 1. **Are there any other measures introduced either in the community or health facilities that have had an impact on provision of MCH services during the pandemic? (e.g., policy to close certain health facilities or scale back MCH service provision) if yes, please state them and explain how.**   Yes, a policy setting a limit to the number of people that can been seen in a health facility per day may have contributed to the decline in usage of MCH services. For example, when you supposed to go a health facility that allows only twenty patients a day and if you have had the experience of being denied entry before, you may stop coming for MCH services. so, any pregnancy issue you have, it may end up being taken care of at home. Or at the end of the day, they woman may end up losing her life or the unborn child. So, it had a negative consequence, but this is the Gambia where people don’t try to find out the root causes of maternal and neonatal deaths in the Gambia. |
