# Supplementary material for: Factors that influenced utilization of antenatal and immunization services in two local government areas in The Gambia during COVID-19: An interview-based qualitative study
Source: PLoS One. 2023 Jun 29;18(6):e0276357. doi: 10.1371/journal.pone.0276357 (PMC10309596; doi:10.1371/journal.pone.0276357)
Supplement: S1 File — (ZIP) [file pone.0276357.s001.zip › Supporting information /Health worker 1.docx]

In-depth interview questionnaire for health workers

**Introduction and Consent**

Hello, my name is Abdourahman Bah. I am a final year (MRC sponsored) BSc Global Health student at Queen Mary University of London. I am interviewing health workers and mothers in The Gambia to learn about the impacts of Covid-19-related lockdown measures on utilisation of mother and child services. The interview will take about 30 minutes. All the information I obtain will remain strictly confidential. You may choose not to answer any question that makes you feel uncomfortable.

Do you have any questions?

Do you agree to being interviewed? Yes

| **Background** |
| --- |
| 1. **Could you please tell me where you live?**   I live in Tallinding   1. **What is your profession**?   I am a nurse   1. **What does your role entail?**   We conduct deliveries; we help women during antenatal clinics, and we also help them during their postnatal periods. |
| 1. **Please tell me for how long you have been working in this health facility**.   I have worked here for almost two years now. Since my graduation from nursing school has been two years now. |
| 1. **What motivated you into pursuing a public health career?**   I see nursing as a noble profession. So, my main motivation was to help and save life. It is a very nice job and I really admire the way I see nurses in saving life and when it comes to delivery also, you help to bring someone in the world. So, I thank God for allowing me to pursue such a career. |
| 1. **What MCH services are provided in this facility? Probe: immunisation, antenatal care**   We normally provide antenatal care services, immunisations services, and we also do disease investigations for pregnant women, such a Hepatitis.   1. **Did the provision of these services continue during the pandemic?**   In this facility, the provision of these services continued unstopped even during the pandemic. However, some women were not coming for these services during the pandemic. |
|  |
| 1. **Have you noticed any changes in utilisation of MCH services during the pandemic? For example, do you see fewer or more patients than usual?**   Yes, we experienced a changed in the number of people coming as some people stopped coming during the pandemic. This was because they had the fear that if they come, especially when one Bangladeshi man died here due to Covid-19. As a result, some had a sceptical mind about the issue. Even some women in labour preferred to go to another facility instead of coming here, but now the service is back to normal and even to have an antenatal card is becoming a problem due to the large number of people coming here. |
|  |
| **Individual factors** |
| 1. **From the perspective of health workers, how safe do you think it is to provide MCH services during the pandemic?**   It was very tough for us as there no adequate resources. Even the face mask that we wear to have it was sometimes a problem. Working in that condition is not easy but we just risk our lives to come to work at that time. For the mask, we used to wear it throughout. When we sometimes have a suspected case, we allow only our seniors who have PPEs to attend to our patients, but not all of us were able to have a face mask. This difficulty didn’t prevent us from rendering the service. We just risked our lives to help others. |
| 1. **How safe is for women to access MCH services in this facility at that period?**   It was not safe for them. You know during pregnancy; you are vulnerable to many diseases. It was not safe for them, but they just had to come and protect themselves by following the precautionary measures. They should also continue wearing face mask which may save them from getting the disease. They should also maintain social distancing and maintain proper hand hygiene. |
| 1. **Did you or your colleagues work more or less hours during the lockdown? If yes, please explain why?**   Yes, there was a time when they made arrangements, in which the staff were divided into two groups. One group would work for three days, while the other group takes the three days off. This was done to prevent congestion in the hospital. This allowed social distancing to be maintain and reduce the contact hours that staff have with each other. These are some of the reasons why this was done. This, however, did not affect our working hours but just meant that the number of health workers working at the same time was reduced. |
| **Interpersonal factors** |
| 1. **What is your family’s attitude in your provision of MCH services during the pandemic? (Are they supportive or not? If yes, explain how?**   It was tough for me, especially for my husband, he would say, you are risking your life for this work. I would tell him to just let me go. He would tell me, if you go and get infected, then that would be your problem not mine. If I insist, he will say, I will just pray for you to go work safe and come back safe. You just make sure that you follow the precautionary measures, such as wearing the PPEs, he would advise me. He used to also advise me not to mingle with people, especially pregnant women. He, however, never told me to go to work as he likes nursing and always supports me. He would say if you help people, God will help you in return. |
| 1. **Have you noticed any changes in your colleagues’ attitudes in providing MCH services during the pandemic? probe: did you experience a reduction in staff’s work appetite? If yes, explain why (maybe due to lack of risk allowance and patient overcrowding)**   Of course, some of my colleagues were not willing to come to work. Some were not willing to come because they had problems with their partners, who were not supportive. Some were sick during that period and were stopped from coming to work until when they recovered.   1. **What incentives were provided by the government to motivate health workers during the pandemic?**   We didn’t receive any incentives from the government. There was a time when they give us Covid-19 allowance, but that was a very small amount. They also provided us with face mask but even that mask used to run out. So, we had to buy it outside using our own money just for us to be able to come to work. |
| 1. **What is your attitude towards MCH service users during the pandemic? probe: were they making your work easier or more difficult?**   Some were very difficult to deal with. Some even if you tell them to wear a face mask when entering the labour room. They would tell you that they will not wear it since they do not have Covid-19. The rule we have in the hospital here is that if you don’t wear a face mask, we are not going to provide the service to you. So, if you want our help, you will need to wear a face mask like we do ourselves. Even though some were not willing to follow the precautionary measures, we force them to comply. Because we cannot risk our families by coming to work and at the same time, our patients are refusing to follow advice, so that that makes our work difficult. So, they have to accept in order to receive the service. |
| **Community factors** |
| 1. **Have you experienced any changes in people’s perception in the community about the use of MCH services during the pandemic? if yes, explain**.   Yes, there was a friend of my husband, who had a serious cold. He said he was afraid to go the health facility because they might say he has Covid-19. So, instead of going to the health facility, he came to my place so that I can help him with the sickness. |
| 1. **Have you experienced any challenges in providing MCH services due to transport difficulties? if yes, explain how**   Yes, there was a time when people were not going out that much, but since I was not living very far from the health facility, I used to walk to here. |
| **Institutional factors** |
| 1. **What do you think of the quality of care provided by this health facility during the pandemic?**   The quality of service was very much affected, because during those days, some of our staff were refusing to provide the service due to lack of PPEs. So, some of the patients were also refusing to buy face mask, so that also demotivated some staff from providing the service. So, sometimes they was this push and pull between some of our staff and some patients. So, that made our work very difficult. Also, there was also a time when there was not mask available. Everybody was scared to work. Sometimes, we also could not even have gloves. So, resources were a problem that time. |
| 1. **Do you think this health facility had adequate medical supplies during the pandemic? if no, give reasons.**   We experienced a shortage of medical supplies as well. Sometimes even I.V fluid, Zinc syrup and vitamin C were scarce in the pharmacy as there were in high demand at that time. the shortage of medical supplies could have a been reason why some people were not coming to health facilities because for example, if I leave my home and come here and don’t have the medicines that I need, I will go home and not come back here in the short distant future. So, I will not even come here again, and I would prefer to go to a pharmacy instead.   1. **Do you think this health facility had adequate PPEs during the pandemic? if no, give reasons. Did that have any effect on your willingness or ability to provide MCH services?**   Yes, because you cannot risk your life too much. You leave your family and come to work, where you can get infected very easily and you don’t have the necessary PPEs. So, that one is very difficult. Since not all of us can have PPEs, instead of all of us attending to a patient we only allow our seniors who have the PPEs to attend to the patient. So, for the rest of us, we just used the face mask. That is how we used to operate here. |
| 1. **Do you think this facility had enough manpower to provide MCH services during the pandemic? if no, give reasons**   One of our colleagues was infected. Other three were also asked to self-isolate at home. So, while they were away, it was very difficult for the rest of us here. The workload was too much.   1. **What do you think of the health facility environment? Probe: is the facility clean and not overcrowded?**   It was not conducive as everybody was scared, but that did not prevent me from coming to work. |
| **Policy factors** |
|  |
| 1. To prevent infection in health facilities, infection prevention and control measures, such as mandatory screening, wearing of PPEs and face mask, have been introduced in many health centers. What is the effect of these practices on provision of MCH services? |
| 1. **What is the effect of these measures on utilisation of MCH services during the pandemic?**   There had a huge impact on people’s willingness to come for MCH services because when they come here, their temperatures are checked. For those with health problems, we just encourage them to wear a face mask. If you are in a place where there are few people, you can remove your mask, but if you are in the facility, you can get many illnesses there. That is, why I say at that time it when it was the peak of the pandemic, we do not allow people to enter the delivery ward without a face mask. You wear yours while I wear mine so that we can protect each other. I cannot wear mine while you don’t wear yours. If you don’t wear your mask, we will not help you even if the baby is coming because it is not safe. |
| 1. **Are there any other factors that may have negatively impacted your ability to provide MCH services during the pandemic that I haven’t asked you about? if yes, please state them and explain how?**   At that time, my child was sick. Sometimes if I want to come to work during the pandemic when everybody is staying at home, it becomes difficult for me to come and leave my sick child at home. So, for that reason, sometimes I used to come to work but not feeling happy, but I just try.   1. **Are there any other factors that may have contributed to the decline in the use of MCH services during the pandemic that I haven’t asked you about? If yes, please state them.**   For some women, they think that if they come will get infected with the virus, especially when that man died here. They used to say that everybody at BMCHH has the virus so if I go there, I will get it myself. So, for that reason, thy stopped coming here. At that time, you can even count the number of deliveries we have here. Sometimes we have about only two deliveries in the morning and maybe five in the evening while before the pandemic we used to have about eight deliveries during the day and about fifteen at night. So, many people ran away from health facilities at that time.   1. **To prevent the decline in use and provision of MCH services in the event of another pandemic or second wave, what do you think the government should do?**   For the first time, they said there was a vaccine, and everybody is now taking that vaccine. For the health workers, they should help us with more PPEs if not the work is going to be too much. You cannot risk your life and leave your family at home and come to work and get in contact with different people. I would also advise my colleagues to take the vaccine. I would also advise the government to work on providing enough vaccines as some people have taken their first doses but hasn’t taken the second dose since it is not available.   1. **What advice would you give to people who are not using MCH services during the pandemic?**   The advice I would give them is that if you are already sick, you cannot just sit at home, especially pregnant women, and say it is because of the pandemic. you should come out and go for your antenatal care where they will check you. You should just follow the precautionary measures to protect yourself and your family. You should also maintain a good hygiene. |
|  |
