# Supplementary material for: Factors that influenced utilization of antenatal and immunization services in two local government areas in The Gambia during COVID-19: An interview-based qualitative study
Source: PLoS One. 2023 Jun 29;18(6):e0276357. doi: 10.1371/journal.pone.0276357 (PMC10309596; doi:10.1371/journal.pone.0276357)
Supplement: S1 File — (ZIP) [file pone.0276357.s001.zip › Supporting information / Respondent 3.docx]

In-depth Interview Questionnaire for MCH service Users

**Introduction and Consent**

Hello, my name is Abdourahman Bah. I am a final year (MRC sponsored) BSc Global Health student at Queen Mary University of London. I am interviewing health workers and mothers in The Gambia to learn about the impacts of Covid-19-related lockdown measures on utilisation of mother and child services. The interview will take about 30 minutes. All the information I obtain will remain strictly confidential. You may choose not to answer any question that makes you feel uncomfortable.

Do you have any questions?

Do you agree to being interviewed? Yes

| **Background** |
| --- |
| 1. **Could you please tell me where you live – Probe: house of residence is?**   I live in Tabokto |
| 1. **Please tell me how you got here today? Probe: public transport, private or walked.**   I got here today by using public transport  **14. Was the health facility doing any of the following: hand washing? use of sanitizers? Measuring the temperatures? Isolation wards? Any Other things that you have observed that are different during the non-COVID times?**  During the Covid-19 pandemic, they stopped weighing our children we bring them for immunization. They have still not resumed weighing our children to know their weight. So, we were only bringing our children when they needed to be immunized which was every two months. |
|  |
| 1. **Have you changed the way you access this service during the outbreak? If so, how? If you have changed, are you going more times or less times and if so, what are the reasons? Probe-economic? Fears?**   I was not bringing my child regularly for immunisation during the Covid-19 pandemic. I did not bring my child for immunisation for about three months. This was because of the Covid-19 pandemic. |
|  |
| **Individual factors** |
| 1. **How safe do you think it is to access MCH services during the pandemic? - Probe: have these concerns stopped you from using these health facilities?**   It was not safe, but your child’s health should be your number one priority. You have to sacrifice for your child. I was coming for the sake of my child. |
| **Interpersonal factors** |
| **18.What is your family’s attitude, including your husband, in your use of MCH services during the pandemic? Probe: Do they encourage or discourage you? In what way?**  My husband was supportive, but there were some members of my family who were telling me not to go to the health facility because of the Covid-19 pandemic. |
|  |
| **Community factors** |
| **20.Have you noticed any changes in people’s perception in your community about the use of MCH services during the pandemic? if yes, explain. Probe: give examples of people being afraid of visiting facilities due to stigma associated with visiting health facilities or fear of being quarantined etc.**  Some of people in my community were not bringing their children for immunisation because they were afraid to come. This was the reason they were not coming to the health facility as they believe that they can easily get infected at the health facility. |
| **21.Has this had any impact on your use of MCH services during the pandemic? if yes, explain how**  This did not prevent me from bringing my child to the health facility but could definitely prevent other from going to health facilities because of the rumours that people were saying regarding the health facility being a source of infection. |
| **22.Have you experienced any challenges on getting to health facilities during the pandemic? if yes, state them (e.g., lack of transport)**  I did not experience that because I usually come here around mid-day, when there is usually less traffic and few passengers. However, at that time, I had to board two vehicles to get here because there were few vehicles operating at that time due to social distancing measures. |
| **Institutional factors** |
|  |
|  |
| **23.Do you think this health facility had adequate medical supplies during the pandemic? if no, give reasons. Probe- has this stopped from visiting health facilities.**  When I my child got, I would not have all the medical supplies I needed. I would get some and they would prescribe some for me to buy at pharmacy, which was not always easy because of financial issues. This was a reason why some even stopped coming to the health facility because they would say that it is better to go straight to the pharmacy than to come to the health facility because they would not get the medicines, they need at the health facility. For me, I feel like going to the health facility is very important because if you come to the health facility, they would take you to the lab and check you which is not always the case in private pharmacies. |
|  |
| **24.What are your perceptions about the health workers in this facility? (e.g., competence or behaviour of health workers). probe- has this stopped you from visiting health facilities.**  I was happy with their behaviour, and I did not have any problem with them. |
| **25.Do you think the health workers were following the Covid-19 precautionary measures appropriately? For example, were they always wearing face mask and PPEs? Probe-has this stopped from visiting health facilities?**  Yes, I used to see all of them wearing face mask. |
| **Policy factors** |
|  |
| **26.To prevent infection in health facilities, infection prevention and control measures, such as mandatory screening, wearing of facemask and social distancing, have been introduced in many health centers. What do you think of the implementation of these measures in the health facilities? Probe: were they implemented correctly?**  To come into the health facility, it was mandatory to wear face mask and wash your hands. I know some women do not feel comfortable to wear face mask, but for me, I know is for own safety, so I have to put it on to protect myself and my child. I am Asthmatic, but I have to endure it and put the mask. In this health facility, even if you explain this to the health workers, they will not allow you to get in without the mask, so I had to bear it and put on the face mask, but I know this could be a reason why some women were not willing to come to the health facility. |
|  |
|  |
| **27. Was there any other barrier to accessing health care services during the pandemic that I did not ask you about?**  If you come here, you would not have anywhere to wash your hands. They only consider face mask, which was their priority. Even right, I want to wash my hands, but I cannot find a place where I can wash them. They should have taps around the health facility. It is only available at the main gate, which is not enough for everybody. This issue makes the patients feel unsafe, since your child is not putting on a face mask, you should not breastfeed without first washing your hands, and for me, I used to go to the nearby compounds to get water.  **28. What do you think the government should do to prevent a decline in use of MCH services in the event of another pandemic?**  The government should provide free face masks for people in the health facilities. This should be made available at the main gate because some people may not be able to afford the cost of a face mask. So, the government should face mask free of charge for people coming into health facilities. The government should also encourage mothers to make sure that their children put on face mask when coming to the health facility. This will help to protect the children as well.  **29. What advice would you give to people who are not using MCH services during the pandemic?**  They should bring their children for immunisation to the health facilities because their children’s health should be their main priority. So, they should not say that there is Covid-19 at the health facility, which prevents them from bringing their children for immunisation. They need to bring their children so that they can get the right vaccine at the right time. Some mothers thought that if they bring their children for immunisation, they will be given the Covid-19 vaccine, but I think that before you are given the Covid-19 vaccine, you will have to give consent and you must be eighteen years of age. |
