# Supplementary material for: Factors that influenced utilization of antenatal and immunization services in two local government areas in The Gambia during COVID-19: An interview-based qualitative study
Source: PLoS One. 2023 Jun 29;18(6):e0276357. doi: 10.1371/journal.pone.0276357 (PMC10309596; doi:10.1371/journal.pone.0276357)
Supplement: S1 File — (ZIP) [file pone.0276357.s001.zip › Supporting information /Health worker 13.docx]

In-depth interview questionnaire for health workers

**Introduction and Consent**

Hello, my name is Abdourahman Bah. I am a final year (MRC sponsored) BSc Global Health student at Queen Mary University of London. I am interviewing health workers and mothers in The Gambia to learn about the impacts of Covid-19-related lockdown measures on utilisation of mother and child services. The interview will take about 30 minutes. All the information I obtain will remain strictly confidential. You may choose not to answer any question that makes you feel uncomfortable.

Do you have any questions?

Do you agree to being interviewed? Yes

| **A** |  |
| --- | --- |
| A | **Introduction** |
| 1 | **Could you please tell me where you live?**  I live in Brikama |
| 2 | **Could you please tell me what your profession is?**  I’m a public health officer |
| 3 | **What does your role entail?**  We have numerous roles that we play. Our roles are not only limited in this hospital here. We go for environmental inspections. We also go for occupational health inspections. We also do immunisations, surveillance and many other things. |
| 3 | **Please tell me for how long you have been working in this health facility?**  I have been working here for almost three years |
| 4 | **What motivated you into pursuing a public health career?**  What motivated me is to help my community. |
| 5 | **What MCH services were provided in this facility during the pandemic?**  In our area, we provided immunisations. Even before the pandemic, we provided immunisations. |
| 7 | **Have you noticed any changes in utilisation of MCH services during the pandemic? For example, do you see fewer or more patients than usual?**  Yes. Some were of the notion that we the health workers told them to stay at home, while we did not communicate that to them. Covid-19 is something that is dangerous but the diseases that we are preventing are also dangerous. So, we have to prevent these ones also. I can say there was a reduction in the number of people coming for MCH services, but that lasted for just three months, when people re-started coming. |
| B | **Individual factors** |
| 8 | **From the perspective of health workers, how safe do you think it is to provide MCH services during the pandemic?**  I’m not going to say that it is safe because even the environment that you are working in is not safe, but what we do is to use PPEs such as face mask and hand sanitisers and we monitor the temperature of anyone coming into the hospital. We also provide a hand-wash station. What we used to do is that whoever does not put on a face mask will not receive any service. If you don’t use a face mask, that means you are putting others at risk, who may be very vulnerable to the disease. This includes children under five since they cannot wear a face mask, so you the elder needs to wear a face mask to protect them. So, if you don’t wear a face mask, you will not receive our services. even those who are not comfortable in wearing a face mask, we told them they have to wear a face mask as long as they in a crowd because we don’t know if you are infected or not. |
| 10 | **Did you or your colleagues work more or less hours during the lockdown? If yes, please explain why?**  Our working hours during the pandemic was more or less the same. |
| C | **Interpersonal factors** |
| 11 | **What is your family’s attitude in your provision of MCH services during the pandemic? (Are they supportive or not?**  I can say as in every family, everyone is worried about his/her family member, so they were concerned and worried about me. I always tell them, I am going to protect myself, so I was able to convince them and because they have trust in me. They sent me to school, so I also use that opportunity to sensitise them about the disease. |
| 12 | **Have you noticed any changes in your colleagues’ attitudes in providing MCH services during the pandemic?**  At that time, our seniors always monitor us to make sure that we are putting on our PPEs. I didn’t notice any changes in my colleagues’ desire to provide MCH services. I only noticed that in one student, but not among our staff. Every health worker before you choose this career, you already know the risks involved in this job. This kind of situation is not new to us, so can’t say it is not safe, so you can’t provide MCH services. In a hospital, everyone is at risk because you can get a disease at any moment. |
| 13 | **What is your attitude towards MCH service users during the pandemic?**  Some of them are complicated because some of them may understand things easily while others may make things more difficult. |
| D | **Community factors** |
| 14 | **Have you experienced any changes in people’s perception in the community about the use of MCH services during the pandemic? if yes, explain.**  I can say yes. Some would say I will not go the hospital because the hospital is the source of infection. Even if someone gets ill, they rather stay home than come to the health facility and receive medical attention. |
| 15 | **Have you experienced any challenges in providing MCH services due to transport difficulties? if yes, explain how**  I didn’t experience any difficulties in getting to health facilities during the pandemic because at that time I was having a bicycle. |
| E | **Institutional factors** |
| 16 | **Do you think there is adequate health facilities to provide MCH services during the pandemic? if no, state reasons**  The health facility was continuously open. Even though we had some positive cases here, only some departments were closed for fumigation. |
| 18 | **Do you think this health facility has adequate medical supplies and PPEs during the pandemic? if no, give reasons.**  We experienced a shortage of PPEs during the pandemic and that is still the case here. Even for hand sanitisers, we experienced a shortage. We were donated some, but the ones from the ministry was not enough. We managed with the little we had. |
| 19 | **Do you think this facility has enough manpower to provide MCH services during the pandemic? if no, give reasons**  I can say we had manpower issues at that time. In our department for instance, we were mounting two border posts. So, some of our staff were deployed to those border posts. In Brikama here, we also have a long queue, because we don’t have enough seats, so I can say that’s something normal here. Even though some people stopped coming, the long queue continued. Practising social distancing was not possible here and I don’t think that would be possible in this health facility. What we used to do is to allow them to sit in five’s but when we they get close to us, they sit in two’s. for them, you can’t control them because there are not enough seats. |
| F | **Policy factors** |
| 22 | **Has these measures had any impact on utilisation of MCH services during the pandemic? if yes, explain how**  These measures may have prevented some people from coming to health facilities at that time. This consequently led to low coverage of MCH services. |
| 23 | **Are there any other measures introduced either in the community or health facilities that have had an impact on provision of MCH services during the pandemic? (e.g., policy to close certain health facilities or scale back MCH service provision) if yes, please state them and explain how.**  One of the challenges we had in providing MCH services was the attitude of people towards the Covid-19 pandemic. Some believed that Covid-19 did not exist. |
