# Supplementary material for: Factors that influenced utilization of antenatal and immunization services in two local government areas in The Gambia during COVID-19: An interview-based qualitative study
Source: PLoS One. 2023 Jun 29;18(6):e0276357. doi: 10.1371/journal.pone.0276357 (PMC10309596; doi:10.1371/journal.pone.0276357)
Supplement: S1 File — (ZIP) [file pone.0276357.s001.zip › Supporting information /Respondent 8.docx]

In-depth Interview Questionnaire for MCH service Users

**Introduction and Consent**

Hello, my name is Abdourahman Bah. I am a final year (MRC sponsored) BSc Global Health student at Queen Mary University of London. I am interviewing health workers and mothers in The Gambia to learn about the impacts of Covid-19-related lockdown measures on utilisation of mother and child services. The interview will take about 30 minutes. All the information I obtain will remain strictly confidential. You may choose not to answer any question that makes you feel uncomfortable.

Do you have any questions?

Do you agree to being interviewed? Yes

| **Background** |
| --- |
| 1. **What is your ethnicity?**   I am a Jola   1. **What is your religion?**   I am a Muslim   1. **What is your marital status?**   I am married   1. **Could you please tell me where you live – Probe: house of residence is?**   I live in Jeshwang. I brought my child for immunisation today at this health facility, but I usually take him to Serrekunda health centre. |
| 1. **Please tell me how you got here today? Probe: public transport, private or walked.**   I got here by using public transport. |
| 1. **Have you used MCH services during the pandemic? if yes, what MCH service have you used during the pandemic?**   During the pandemic, I was staying in Fagikunda. I used to go to Fagikunda health centre for antenatal care, but the health centre was later closed because of the pandemic. Even before that I was admitted at Banjul hospital. I delivered there and admitted there at that time. That was a very difficult time for me, as I could not even have brought to me from home. I used to eat the food prepared at the hospital. Sometimes I could not even eat that food because of how awful it was. It was other people that I was admitted with that were helping me. although my husband works hard, he is not wealthy. He has no other family members. He has not brothers or sisters and his parents have passed away. It went to a level that we could not even afford to pay rent. So, we were driven out of the house. This all happen during the pandemic. My child is also not well but we have to thank God despite the difficult circumstance. |
| 1. **Have you changed the way you access this service during the outbreak? If so, how? If you have changed, are you going more times or less times and if so, what are the reasons? Probe-economic? Fears?**   I used to go to Fagikunda health centre for antenatal care, but the health centre was later closed because of the pandemic. So, because of that I stopped going for antenatal service. I later went there and found a health worker there. I told him that I used to come to this health facility, but I would not find anyone here and I was told that the health facility has been closed. He told me that was a lie. The health facility was closed only for few days. It was closed so that we could fumigate it because we had a positive case here. That was the reason it was closed for few days. After which, I re-started taking my child there for immunisation until I moved to Jeshwang and then I started taking him to Serrekunda health centre. |
|  |
| **Individual factors** |
| 1. **How safe do you think it is to access MCH services during the pandemic? - Probe: have these concerns stopped you from using these health facilities?**   If you don’t take your child to the health facility, you will be risking his wellbeing. |
| 1. **Have you experienced any financial difficulties (e.g., transport costs) in accessing MCH services during the pandemic? if yes, explain. Probe- have these difficulties stopped you from using these health facilities?**   For me fare was not the main problem I had with regards to transport because I was not having much money at that time, but I was lucky to sometimes board some vehicles without paying any fare. Some drivers understood my situation and exempted me from paying any fare. Other people would also pay my fare for me as I could not afford to pay. Also, I would sometimes use the money I received during my child’s naming ceremony to pay fare to get to the health facility. |
| **Interpersonal factors** |
| **18.What is your family’s attitude, including your husband, in your use of MCH services during the pandemic? Probe: Do they encourage or discourage you? In what way?**  My husband was very supportive at that time. He would tell me to go the health facility regularly. If I feel lazy to go to the health facility, he will force me to go to the health facility. He was even the one who told me to join the MRC study so that our child will get better and regular service. He has no problem when it comes to our health. He is wealthy, but he values our wellbeing. |
|  |
| **Community factors** |
| **20.Have you noticed any changes in people’s perception in your community about the use of MCH services during the pandemic? if yes, explain. Probe: give examples of people being afraid of visiting facilities due to stigma associated with visiting health facilities or fear of being quarantined etc.**  I don’t know much of what is going on in my community. I am always in my house. I don’t go to other people’s houses. If you are not wealthy, you should know how to handle yourself. I don’t have any friends. My only friends are my husband and my children. |
| **Institutional factors** |
|  |
|  |
| **25.Do you think this health facility had adequate medical supplies during the pandemic? if no, give reasons. Probe- has this stopped from visiting health facilities.**  In the health facilities that I visited, there were not enough medical supplies. Whenever I go there, they would prescribe the medicines for me and tell me to buy it from the pharmacy. Sometimes, I would keep the prescription with me for a week before I would be able to get the money to buy the medicines. So, to be frank with you, there were not enough medicines at the health facilities during the pandemic. The only medicines that they had was paracetamol. You had to buy the rest in the pharmacy. However, it is worth noting that the medical supply shortage in The Gambia was there even before the pandemic, but the issue was exacerbated during the pandemic. |
| **27.What are your perceptions about the health workers in this facility? (e.g., competence or behaviour of health workers). probe- has this stopped you from visiting health facilities.**  I know that some health workers are difficult to deal with, but for me, if they make things difficult for me, I always keep in remember that I need their service, so I need to be patient. They always have problems with people, but for me, I know how to deal with this issue |
|  |
| **Policy factors** |
|  |
| **30.To prevent infection in health facilities, infection prevention and control measures, such as mandatory screening, wearing of facemask and social distancing, have been introduced in many health centers. What do you think of the implementation of these measures in the health facilities? Probe: were they implemented correctly?**  Yes, they would always ask us to wear a face mask, wash our hands and observe social distancing. If you don’t follow these measures, they would not allow you to get into the health facility. |
| **31.What is the effect of these measures on your use of MCH services during the pandemic?**  These measures did not prevent me from going to the health facility. I would put on the face mask until I get into the health facility and then remove it. I remove it because I don’t feel comfortable putting on a face mask. In these health facilities, no matter how difficult it is for you to put on a face mask, you must put it on for you to be allowed entry into the health facility. |
|  |
| **34. Was there any other barrier to accessing health care services during the pandemic that I did not ask you about?**  It was the fact that when we brought our children for immunisation, they would not be weighed. They stopped weighing children during the pandemic. This, however, did not prevent me from bringing my child for immunisation during the pandemic.  **35. What do you think the government should do to prevent a decline in use of MCH services in the event of another pandemic?**  The government does not help people. The government has never helped me. they should help us in improving our wellbeing, by ensuring that there are adequate medical supplies at the health facilities. When you go to the health facility, you will have to buy an entry ticket for 25 dalasi and pay up to 150 dalasi for laboratory test and you would have even paracetamol at the health facility. So, the government should help us with medicines. The government should prioritise people’s wellbeing because even if you are wealthy, but you are not healthy, you will not enjoy your wealth.  **36. What advice would you give to people who are not using MCH services during the pandemic?**  People who were coming for either antenatal care or immunisation, should come. If they don’t come, the consequence of that will be immense. If you want your child to benefit you later in life, you must struggle for that child. Children don’t forget. Whatever you do for them, they will remember it. If you don’t struggle for your children while they were young, they will not struggle for you when you get old. |
