# Supplementary material for: Factors that influenced utilization of antenatal and immunization services in two local government areas in The Gambia during COVID-19: An interview-based qualitative study
Source: PLoS One. 2023 Jun 29;18(6):e0276357. doi: 10.1371/journal.pone.0276357 (PMC10309596; doi:10.1371/journal.pone.0276357)
Supplement: S1 File — (ZIP) [file pone.0276357.s001.zip › Supporting information /Respondent 10.docx]

In-depth Interview Questionnaire for MCH service Users

**Introduction and Consent**

Hello, my name is Abdourahman Bah. I am a final year (MRC sponsored) BSc Global Health student at Queen Mary University of London. I am interviewing health workers and mothers in The Gambia to learn about the impacts of Covid-19-related lockdown measures on utilisation of mother and child services. The interview will take about 30 minutes. All the information I obtain will remain strictly confidential. You may choose not to answer any question that makes you feel uncomfortable.

Do you have any questions?

Do you agree to being interviewed? Yes

| **Background** |
| --- |
| 1. **How old are you?**   I am 18 years old   1. **What is your ethnicity?**   I am a Mandinka   1. **What is your religion?**   I am a Muslim   1. **What is your marital status?**   I am married   1. **Could you please tell me where you live – Probe: house of residence is?**   I am from Latrikunda. |
| 1. **Please tell me how you got here today? Probe: public transport, private or walked.**   My house is not very far from here, so I just walk from my house to the health facility. |
| 1. **Have you used MCH services during the pandemic? if yes, what MCH service have you used during the pandemic?**   I was pregnant at the start of the pandemic. At that time, I used to go for antenatal care in Banjul. I even delivered in Banjul. They stopped weighing children, so you would not be able to know whether your child is growing or not. |
| 1. **Have you changed the way you access this service during the outbreak? If so, how? If you have changed, are you going more times or less times and if so, what are the reasons? Probe-economic? Fears?**   I used to go for antenatal care whenever I had an appointment and after my delivery, I started bringing my child for immunisation every month. |
|  |
| **Individual factors** |
| 1. **How safe do you think it is to access MCH services during the pandemic? - Probe: have these concerns stopped you from using these health facilities?**   At that time, even if a place is safe, you will still feel unsafe. This is because everyone is afraid of getting infected especially in the health facilities. When you come here, you always fear that you may get infected before you the hospital. However, I have never seen with my own eyes anyone with Covid-19. I only saw it on TV and heard about it on the radio. This made me feel unsafe to come to the health facility, but I had to come because of my pregnancy. I knew that if I did not come, I may end up developing some complications because I would not be able to know until it maybe to late |
| 1. **Have you experienced any financial difficulties (e.g., transport costs) in accessing MCH services during the pandemic? if yes, explain. Probe- have these difficulties stopped you from using these health facilities?**   I used to go to Banjul for antenatal care. I used to experience transport difficulties during that time. Transport fares got increased and sometimes, I had to hire a vehicle just to take me there because of the shortage of vehicles at that time. However, my husband has a car that he would use to take me to the hospital at times. The transport difficulty did not prevent me from going regularly for antenatal care during the pandemic. |
| **Interpersonal factors** |
| **18.What is your family’s attitude, including your husband, in your use of MCH services during the pandemic? Probe: Do they encourage or discourage you? In what way?**  My husband is a health worker. So, he understands the importance of going regularly to the health facility. As I said earlier, he would even sometimes take me to the hospital on his vehicle. My entire family was supportive of me, and I never experienced any stigma from them because of going to the hospital. |
|  |
| **Community factors** |
| **20.Have you noticed any changes in people’s perception in your community about the use of MCH services during the pandemic? if yes, explain. Probe: give examples of people being afraid of visiting facilities due to stigma associated with visiting health facilities or fear of being quarantined etc.**  There were people in my community who were either supposed to go for antenatal care or take their children for immunisation, but they were not going to the health facility regularly. I don’t know the reason why they were not attending their appointments, but I know that they were not going regularly. |
|  |
|  |
| **Institutional factors** |
| **23.Did the health facilities stay open during the pandemic? if no, state how this may have affected your access to MCH services.**  When I was pregnant, I used to go for antenatal care in Banjul and the hospital was never closed during that time. When I delivered, I started bringing my child to this hospital and have never come and found it closed. |
|  |
| **25.Do you think this health facility had adequate medical supplies during the pandemic? if no, give reasons. Probe- has this stopped from visiting health facilities.**  When I was going to Banjul Poly Clinic, I used to have all the medicines that I needed. Since I moved to this hospital, I have been getting all the medicines I need here as well. |
|  |
| **27.What are your perceptions about the health workers in this facility? (e.g., competence or behaviour of health workers). probe- has this stopped you from visiting health facilities.**  Whenever I come to the hospital, I used to get the service that I came for. I have never had any problem with the health workers. I did everything that they asked me to do such as wearing face mask, washing my hands regularly and observing social distancing. I believe as long as you follow these rules, you will not have any problem with them. |
| **28.Do you think the health workers were following the Covid-19 precautionary measures appropriately? For example, were they always wearing face mask and PPEs? Probe-has this stopped from visiting health facilities?**  Whenever I went to the hospital, I would see the health workers putting on face mask and they would always observe social distancing. Also, they would always have a bucket of water nearby were they would wash their hands regularly. |
| **Policy factors** |
|  |
| **30.To prevent infection in health facilities, infection prevention and control measures, such as mandatory screening, wearing of facemask and social distancing, have been introduced in many health centers. What is the effect of these measures on your use of MCH services during the pandemic?** |
| For me, following these rules was not problem for me because I knew that they are there to protect me and those close to me. so, I would always put on a face mask, wash my hands regularly and keep a safe distance from others.  **32. What do you think is the effect of these measures on other people’s willingness to come for MCH services?**  However, I know that some people don’t like putting on a face mask, but I don’t really know if this could prevent some people from coming to the health facility. |
|  |
| **35. What do you think the government should do to prevent a decline in use of MCH services in the event of another pandemic?**  The government should help us with more medical supplies. I know some hospitals have adequate medicines but this not the case for every hospital in The Gambia. In some health facilities, you will not have all the medicines you need unless you buy them from the private pharmacies. So, the government should make sure that there are enough medicines in all the health facilities around the country.  **36. What advice would you give to people who are not using MCH services during the pandemic?**  I would advise them to come because it is good for their health, and it is good for their children’s wellbeing. |
