# Supplementary material for: Factors that influenced utilization of antenatal and immunization services in two local government areas in The Gambia during COVID-19: An interview-based qualitative study
Source: PLoS One. 2023 Jun 29;18(6):e0276357. doi: 10.1371/journal.pone.0276357 (PMC10309596; doi:10.1371/journal.pone.0276357)
Supplement: S1 File — (ZIP) [file pone.0276357.s001.zip › Supporting information /Health worker 6.docx]

In-depth interview questionnaire for health workers

**Introduction and Consent**

Hello, my name is Abdourahman Bah. I am a final year (MRC sponsored) BSc Global Health student at Queen Mary University of London. I am interviewing health workers and mothers in The Gambia to learn about the impacts of Covid-19-related lockdown measures on utilisation of mother and child services. The interview will take about 30 minutes. All the information I obtain will remain strictly confidential. You may choose not to answer any question that makes you feel uncomfortable.

Do you have any questions?

Do you agree to being interviewed? Yes

| **Background** |
| --- |
| 1. **Could you please tell me where you live?**   I live in latrikunda jamang   1. **What is your profession?**   I am a nurse   1. **What does your role entail?**   I work at the antenatal side. I take care of antenatal mothers |
| 1. **Please tell me for how long you have been working in this health facility?**   I have been working here for two years now. |
| 1. **What motivated you into pursuing a public health career?**   I just love nursing. I love to help people. I can’t see someone needing help without me helping them. So, I love giving people my help when they need it. |
| 1. **What MCH services are provided in this facility? Probe: immunisation, antenatal care**   For antenatal mothers, when they come, we give them their antenatal cards, we counsel them, fill their antenatal cards, then we take their vitals. We check their history and family health history. Then we check their weight, their BP and palpation because as you know, you need to know their weight and their blood pressure. That is, to know how it is going because for some antenatal mothers, when they get pregnant, their BP level increases, and you also have to monitor their weight. So that you can know whether the weight is increasing or not. This will help you know where the problem is. You can search where the problem is whether the mother’s weight is increasing   1. **Did the provision of these services continue during the pandemic?**   Yes, continued to provide these services during the pandemic. That is the time they need the service more because you know our antenatal when you are pregnant, your immune system goes down. So, it is easy for them to get sick. |
| 1. **Did the health facility stay open during the pandemic, and for how long?**   There was not time during which this hospital was closed. When we have a Covid-19 case, they just spray the whole environment and that just last for few hours. |
| 1. **Have you noticed any changes in utilisation of MCH services during the pandemic? For example, do you see fewer or more patients than usual?**   So, during the first months of the pandemic, the number of people coming for antenatal services was reduced because they didn’t know how to protect themselves from the virus. So, for the first time it was reduced but later on, the number of people coming came back to normal. This reduction was because of lack of sensitisation. So, they misunderstood the Covid-19 pandemic. so, I think that was why many people stopped coming for antenatal services. Others may also be afraid of getting infected, but as time went on, they were no more afraid as it has now become part of our lives. |
|  |
| **Individual factors** |
| 1. **From the perspective of health workers, how safe do you think it is to provide MCH services during the pandemic?**   It was not safe, but since your work, you have to handle it. You have to risk your life. We just tried to protect ourselves by washing our hands, using hand sanitisers and putting on face mask. We also observed social distancing as much as possible despite the fact that there is little space in this hospital which makes it difficult to observe social distancing. |
| 1. **How safe is for women to access MCH services in this facility at that period?**   It was not safe because of over-crowding and some refuse to use face mask. If you tell them to put on a face mask, they get angry because some of them do not believe the existence of Covid-19. That is why they don’t wear face mask. If you tell them to wear it, they will put on in your presence, but they remove it as soon as you leave. Some will be coughing without covering their mouth and nose. So, they would sneeze just like that. So, all of these things made the hospital not safe. If this one is sneezing without covering their mouth and nose, and you are sitting close to them, it makes it very unsafe for you especially considering the fact that social distancing is hardly observed. Also, in our area, which is the antenatal side, the place used to be over-crowded. It is always busy. |
| 1. **Did you or your colleagues work more or less hours during the lockdown? If yes, please explain why?**   We worked more during the pandemic because you know pregnant women, when they are pregnant, their immune system goes down. So, it is very easy for them to get sick. We had some of our antenatal mothers who fell sick, so we had to take care of such people and also for others came for regular appointments. |
| **Interpersonal factors** |
| 1. **What is your family’s attitude in your provision of MCH services during the pandemic? (Are they supportive or not? If yes, explain how?**   My family didn’t give me any difficulty with regards to coming to work at that time. however, in my community, I was stigmatised because of my profession. As result, I stopped going to people’s houses because they would say this one is working in a hospital. So, she may bring Covid-19 to our house. So, I self-isolated in my home and stopped going out. Sometimes, they would say, you nurse, what you are doing is not safe as you are working in a hospital, where you can easily get infected and bring it home with you, unknowingly. I just ignored them and continued doing my job. This is part of our job |
| 1. **Have you noticed any changes in your colleagues’ attitudes in providing MCH services during the pandemic? probe: did you experience a reduction in staff’s work appetite? If yes, explain why (maybe due to lack of risk allowance and patient overcrowding)**   There was no change in their willingness to provide antenatal services. They continued to be highly motivated.   1. **What incentives were provided by the government to motivate health workers during the pandemic?**   We didn’t receive any incentives from the government. |
| 1. **What is your attitude towards MCH service users during the pandemic? probe: were they making your work easier or more difficult?**   The women who came for antenatal services made our lives difficult at that time because they refused to follow the Covid-19 precautionary measures. |
| **Community factors** |
| 1. **Have you experienced any changes in people’s perception in the community about the use of MCH services during the pandemic? if yes, explain.**   Yes, during the start of the pandemic, people were afraid to come to hospital. When they fall sick, they preferred to cure themselves or go to the pharmacy rather than coming to the hospital. |
| 1. **Have you experienced any challenges in providing MCH services due to transport difficulties? if yes, explain how**   Yes, I had transport difficulties at that time. For this public transport, sometimes when you board a vehicle, you meet people who don’t the existence of Covid-19 and as such, do not put a face mask. They will be even sneezing without covering their nose and mouth. You have to bear all these things because you don’t have your own vehicle and you need to come to work. You just wear your face mask and if possible, you observe social distancing. Transport cost was another problem as fares were increased at that time. |
| **Institutional factors** |
| 1. **Do you think this health facility had adequate medical supplies during the pandemic? if no, give reasons.**   In the antenatal area, we didn’t have any shortage of medical supplies as all the medicines we needed were adequately provided.   1. **Do you think this health facility had adequate PPEs during the pandemic? if no, give reasons. Did that have any effect on your willingness or ability to provide MCH services?**   You know at that time, the demand for PPEs was high. So, we had difficulties with PPEs, such as face mask and hand sanitisers, but this was because of the high demand. |
| 1. **Do you think this facility had enough manpower to provide MCH services during the pandemic? if no, give reasons**   We had some nurses who got infected. This affected our ability to provide the service because we had to cover those who got infected. Also, when we have a positive case among our patients, all the nurses that attended to that patient who have to quarantine for two weeks. That also affected our ability to provide antenatal services as it increased our workload.   1. **What do you think of the health facility environment? Probe: is the facility clean and not overcrowded?**   Yes, because we were having our hand washing buckets, hand sanitisers and face mask. We were also sensitising our patients to always wear face mask, wash their hands frequently and when you are sneezing, you must cover your nose and mouth. These measures made us feel somehow safe to provide the service. |
| **Policy factors** |
|  |
| 1. **To prevent infection in health facilities, infection prevention and control measures, such as mandatory screening, wearing of PPEs and face mask, have been introduced in many health centers. What is the effect of these measures on utilisation of MCH services during the pandemic?** |
| For those women who have respiratory problems we understand their situation. so, we exempt them from wearing face mask. We just made sure that we always wear face mask when attending to them. We also ask to observe social distancing since they are not wearing face mask. For those who don’t have money to buy face mask, we give them face mask when they come in if it is available. |
| 1. **Are there any other factors that may have negatively impacted your ability to provide MCH services during the pandemic that I haven’t asked you about? if yes, please state them and explain how?**   The difficulty was that we were at risk and some of the antenatal mothers were difficult to deal with. Some would refuse to wear face mask and sneezing like that. They would say it is just a common cold that they are having so no need to wear a face mask. The availability of face mask, hand sanitisers was another problem. Observing social distancing was another difficultly.   1. **To prevent the decline in use and provision of MCH services in the event of another pandemic or second wave, what do you think the government should do?**   The government should continue encouraging people to come to health facilities and sensitise them about the Covid-19 pandemic via the radio and televisions. This will help people understand what to do and what not to do. The government should provide adequate hand sanitisers and face mask and if it is possible to give the health workers some incentives in the form of allowances so as to encourage the health workers.   1. **What advice would you give to people who are not using MCH services during the pandemic?**   It is just to sensitise people about the Covid-19 pandemic so that they would know how to protect themselves from the disease. If they understand it, they will know how to protect themselves. |
