# Supplementary material for: Factors that influenced utilization of antenatal and immunization services in two local government areas in The Gambia during COVID-19: An interview-based qualitative study
Source: PLoS One. 2023 Jun 29;18(6):e0276357. doi: 10.1371/journal.pone.0276357 (PMC10309596; doi:10.1371/journal.pone.0276357)
Supplement: S1 File — (ZIP) [file pone.0276357.s001.zip › Supporting information /Respondent 4.docx]

In-depth Interview Questionnaire for MCH service Users

**Introduction and Consent**

Hello, my name is Abdourahman Bah. I am a final year (MRC sponsored) BSc Global Health student at Queen Mary University of London. I am interviewing health workers and mothers in The Gambia to learn about the impacts of Covid-19-related lockdown measures on utilisation of mother and child services. The interview will take about 30 minutes. All the information I obtain will remain strictly confidential. You may choose not to answer any question that makes you feel uncomfortable.

Do you have any questions?

Do you agree to being interviewed? Yes

| **Background** |
| --- |
| 1. **How old are you?**   I am twenty-five years old   1. **Could you please tell me where you live – Probe: house of residence is?**   I live in Lamin. |
| 1. **Please tell me how you got here today? Probe: public transport, private or walked.**   I got here by using public transport.   1. **Have you had any health education session for Covid-19?**   If you come here, they would ask you to put on a face mask and maintain social distancing, which as all because of the pandemic.   1. **Have you gone to the health facility during the COVID-19? Were you treated differently? Were there any different procedures? If so, what are they doing that is different?**   Yes, I used to come to the health facility during the Covid-19 pandemic, but not frequently. This is because when then the Covid-19 pandemic started, immunization service was not being offered every month. You would only come when your child needs to be immunized, as weighing was stopped during that time. When I bring my child for immunization, they would usually tell me the month you I am supposed to bring my children again. So, I would wait until that month and then bring him to health facility. Sometimes it used to be every two to three months instead of every month. |
| 1. **Have you changed the way you access this service during the outbreak? If so, how? If you have changed, are you going more times or less times and if so, what are the reasons? Probe-economic? Fears?**   I was coming less frequently during the pandemic. This was not because of fear of infection, but was a policy introduced by the health facility. They stopped weighing children. If they hadn’t stopped weighing, we would have to bring our children every month. They stopped weighing because using the same weighing machine to weigh all the children increases the risk of infection. So, to prevent this, they had to introduce this policy. This also meant that if you bring your child, they will only get the injection which is not done every month, which also means that you would not have to bring your child to health facility regularly. If they hadn’t stopped weighing children, I would not have brought my child to health facility because it is not safe to use the same weighing machine to weigh all the children. |
|  |
| **Individual factors** |
| 1. **How safe do you think it is to access MCH services during the pandemic? - Probe: have these concerns stopped you from using these health facilities?**   I can say on one hand it was safe but on the other it was not safe because I am not a health worker. I was only coming for the sake of my child. It was not safe as I can get infected at any moment at the health facility because when you come here, you get into contact with all kinds of people. So, you can easily get infected even if you follow the rules as not everyone follows the precautionary measures correctly. |
| 1. **Have you experienced any financial difficulties (e.g., transport costs) in accessing MCH services during the pandemic? if yes, explain. Probe- have these difficulties stopped you from using these health facilities?**   Transport was big problem for me during the pandemic because I live all the way in Lamin which is quite far from here. There was shortage of vehicles as many drivers were not working at that time due to social distancing measures introduced by the government. They also increased fares which was another major obstacle. |
| **Interpersonal factors** |
| **18.What is your family’s attitude, including your husband, in your use of MCH services during the pandemic? Probe: Do they encourage or discourage you? In what way?**  He never told me not to come but he would ask me to put on face mask and follow all the Covid-19 precautionary measures so that I can protect myself and my child. I thank God by applying these measures, I did not get infected and nor was my child. |
|  |
| **Community factors** |
| **20.Have you noticed any changes in people’s perception in your community about the use of MCH services during the pandemic? if yes, explain. Probe: give examples of people being afraid of visiting facilities due to stigma associated with visiting health facilities or fear of being quarantined etc.**  Yes, there were several people in my community who stopped taking their children for immunisation during the pandemic, while they were supposed to take them. They would say that they would not take their children for immunisation to the health facility because the health facility is not safe, and they could easily get infected and bring it home to infect their families. |
| **Institutional factors** |
| **23.Did the health facilities stay open during the pandemic? if no, state how this may have affected your access to MCH services.**  I was coming here for antenatal services; I delivered here and brought my child here for immunisation. During all this time, I have never come here and found the hospital closed because of the pandemic. However, before the Covid-19 pandemic, I joined the MRC study here, but when the pandemic started, they stopped coming here. |
| **24.Are you satisfied with the care provided by this health facility during the pandemic? probe: consultation time, treatment and respect from health workers. Has this stopped you from visiting health facilities?**  I was satisfied with the service I received during the pandemic. On one hand the pandemic had a positive impact on the quality of service, but on the other hand, it had a negative impact. If you come here during the pandemic, you would stay here for long time before you can access the service you came for. You would also experience transport difficulties. You would also have to buy a face mask before you would be allowed entry into the health facility even if you don’t have any money on you. Others would sometimes be sent home because they don’t have mask and they children would not be immunised. You would also have to observe social distancing. These were some of the negative impacts of Covid-19 on access to immunisation services during the pandemic. |
| **25.Do you think this health facility had adequate medical supplies during the pandemic? if no, give reasons. Probe- has this stopped from visiting health facilities.**  The availability of medical supplies at this health facility reduced during the pandemic because before the pandemic, I used to have all the medical supplies I needed whenever I came to this health facility but now if you came here, they would sometimes ask you to go and buy the medicines at the private pharmacy. So, I can say this shortage of medical supplies is because of the pandemic because this was not the case before the pandemic. |
| **26.Do you think this facility had enough manpower to provide MCH services during the pandemic? if no, give reasons**  During the pandemic, there were not enough health workers at this health facility because many of them were not working at that time. if there used to be three health workers on duty attending to us, during the pandemic, it was reduced to two health workers. So, this resulted in increased waiting time for us as the service was a bit slow because there were not enough health workers. This, however, did not prevent me from bringing my child for immunisation during the pandemic. I continued to bring my child no matter how difficult the situation was. |
| **27.What are your perceptions about the health workers in this facility? (e.g., competence or behaviour of health workers). probe- has this stopped you from visiting health facilities.**  Since there were not enough health workers at that time, whenever, you come here, you could tell that they are exhausted. For that reason, they get frustrated very easily and become short-tempered. |
| **28.Do you think the health workers were following the Covid-19 precautionary measures appropriately? For example, were they always wearing face mask and PPEs? Probe-has this stopped from visiting health facilities?**  Yes, they were following the Covid-19 precautionary measures effectively as they would all be wearing face mask and putting on gloves when attending to patients. They would also ask people to put on face mask and maintain social distancing. |
| **Policy factors** |
| **30.To prevent infection in health facilities, infection prevention and control measures, such as mandatory screening, wearing of facemask and social distancing, have been introduced in many health centers. What do you think of the implementation of these measures in the health facilities? Probe: were they implemented correctly?**  When entering the health facility, they would ask you to put on a face mask. They would not allow you to enter the health facility without putting on a face mask. |
| **31.What is the effect of these measures on your use of MCH services during the pandemic?**  For me, whenever I am about to go out, I put a mask in my back. I put it on whenever I go out. Putting on a mask has never been a problem for me as I know it is there to protect me and others around me.  **32. What do you think is the effect of these measures on other people’s willingness to come for MCH services?**  However, I know putting on a face mask could be a problem for others, as people are different. There are some people who cannot put it on because of a health condition that they have such as Asthma and other respiratory illnesses. Such individuals cannot put on a face mask and may not be allowed entry into the health facility because the security guards may not understand their situation. |
|  |
| **34. Was there any other barrier to accessing health care services during the pandemic that I did not ask you about?**  When I come here for immunisation, I used to stay here for a very long time. That is, the waiting time increased during the pandemic. this was not the case before the pandemic. So, I can say this was because of the measures during the pandemic such as taking of people’s temperatures, social distancing and ensuring everybody wears a face mask.  **35. What do you think the government should do to prevent a decline in use of MCH services in the event of another pandemic?**  The government should help us in ensuring that speed at which the service is delivered is improved so that when we bring our children, we would not wait here for long before we can receive the service. This can be done by employing more health workers.  **36. What advice would you give to people who are not using MCH services during the pandemic?**  They should bring their children for immunisation because if they don’t, they would be putting their children at risk of vaccine preventable diseases. |
