# Supplementary material for: Factors that influenced utilization of antenatal and immunization services in two local government areas in The Gambia during COVID-19: An interview-based qualitative study
Source: PLoS One. 2023 Jun 29;18(6):e0276357. doi: 10.1371/journal.pone.0276357 (PMC10309596; doi:10.1371/journal.pone.0276357)
Supplement: S1 File — (ZIP) [file pone.0276357.s001.zip › Supporting information /Respondent 5.docx]

In-depth Interview Questionnaire for MCH service Users

**Introduction and Consent**

Hello, my name is Abdourahman Bah. I am a final year (MRC sponsored) BSc Global Health student at Queen Mary University of London. I am interviewing health workers and mothers in The Gambia to learn about the impacts of Covid-19-related lockdown measures on utilisation of mother and child services. The interview will take about 30 minutes. All the information I obtain will remain strictly confidential. You may choose not to answer any question that makes you feel uncomfortable.

Do you have any questions?

Do you agree to being interviewed? Yes

| **Background** |
| --- |
| 1. **How old are you?**   I am twenty-seven years old.   1. **What is your ethnicity?**   I am a Mandinka   1. **What is your religion?**   I am a Muslim   1. **What is your marital status?**   I am married   1. **Could you please tell me where you live – Probe: house of residence is?**   I live in Bundung. |
| 1. **Please tell me how you got here today? Probe: public transport, private or walked.**   I got here by walking but sometimes my husband brings me on his vehicle. |
| 1. **Have you used MCH services during the pandemic? if yes, what MCH service have you used during the pandemic?**   Yes, during the start of the Covid-19 pandemic, I was pregnant. So, during the peak of the pandemic, I was coming for antenatal services. I delivered in March 2021. Since then, I have been bringing my child for immunisation. |
| 1. **Have you changed the way you access this service during the outbreak? If so, how? If you have changed, are you going more times or less times and if so, what are the reasons? Probe-economic? Fears?**   I used to come antenatal care every month. I did not reduce the number of times I was coming for antenatal service during the pandemic. However, the pandemic affected us a lot here because if when we sat down, they would always ask us to leave a safe distance between us. |
|  |
| **Individual factors** |
| 1. **How safe do you think it is to access MCH services during the pandemic? - Probe: have these concerns stopped you from using these health facilities?**   It was not safe at all, but I had come for the sake of my child. I also protected myself by following the Covid-19 precautionary measures, such as wearing of face mask, washing my hands regularly and observing social distancing. |
| 1. **Have you experienced any financial difficulties (e.g., transport costs) in accessing MCH services during the pandemic? if yes, explain. Probe- have these difficulties stopped you from using these health facilities?**   For me, I did not experience any transport difficulty in terms of coming to this health facility because I live very close to the hospital. I would sometimes walk from my home to the hospital and sometimes, my husband would bring me on his vehicle. |
| **Interpersonal factors** |
| **18.What is your family’s attitude, including your husband, in your use of MCH services during the pandemic? Probe: Do they encourage or discourage you? In what way?**  My husband was very supportive of me with regards to coming to the health facility. He used to even bring me here on his vehicle during the pandemic. the rest of my family was also very supportive as no one among them was saying I should not come for antenatal care because of the pandemic. |
|  |
| **Community factors** |
| **20.Have you noticed any changes in people’s perception in your community about the use of MCH services during the pandemic? if yes, explain. Probe: give examples of people being afraid of visiting facilities due to stigma associated with visiting health facilities or fear of being quarantined etc.**  Yes, there was one person in my community who was supposed to bring her child for immunisation but was not coming regularly because according to her, the hospital had stopped weighing children. So, she would only bring her child every two to three months when the child was supposed to be immunised. |
| **Institutional factors** |
| **23.Did the health facilities stay open during the pandemic? if no, state how this may have affected your access to MCH services.**  I have had that the hospital was closed for three days during the start of the pandemic because they had positive case here, who eventually died, but that closure did not coincide with my appointment date. So, for me, the hospital was open whenever I come here. |
| **25.Do you think this health facility had adequate medical supplies during the pandemic? if no, give reasons. Probe- has this stopped from visiting health facilities.**  During my pregnancy, I used to have all the medicines I needed whenever I come for antenatal service at that time. |
| **26.Do you think this facility had enough manpower to provide MCH services during the pandemic? if no, give reasons**  There were enough health workers in the antenatal service department. However, we used to stay here for a long time before we could access the service but that was not because of shortage of health workers, rather it was because of the large number of people coming for antenatal service at that time. when sitting, we would always observe social distancing. This was also another reason why the queue used to be very long. |
| **27.What are your perceptions about the health workers in this facility? (e.g., competence or behaviour of health workers). probe- has this stopped you from visiting health facilities.**  I know that some health workers are difficult to deal with, but for me, I have not had any problem with the health workers during the pandemic. As long as you follow the Covid-19 precautionary measures, you would not have any problem with them, but if you don’t follow these measures, they will give you though time when you come here. |
| **28.Do you think the health workers were following the Covid-19 precautionary measures appropriately? For example, were they always wearing face mask and PPEs? Probe-has this stopped from visiting health facilities?**  The health workers that I have seen during my times here were always following the precautionary measures, such as wearing of face mask, observing social distancing and washing their hands regularly. They would also ask people to follow these measures. They would not allow anyone to get into the health facility without wearing a face mask and washing their hands at the hospital entrance. |
| **Policy factors** |
|  |
| **30.To prevent infection in health facilities, infection prevention and control measures, such as mandatory screening, wearing of facemask and social distancing, have been introduced in many health centers. What is the effect of these measures on your use of MCH services during the pandemic?** |
| For me, following these measures was not a problem for me because I know that they put there to protect me and other around me.  **32. What do you think is the effect of these measures on other people’s willingness to come for MCH services?**  These measures prevented some people from coming to the health facility during the pandemic because they don’t like the idea of wearing of face mask and if they come here without a face mask, they would be denied entry into the hospital. So, for some people, they preferred to just stay home than to come here. |
