# Supplementary material for: Factors that influenced utilization of antenatal and immunization services in two local government areas in The Gambia during COVID-19: An interview-based qualitative study
Source: PLoS One. 2023 Jun 29;18(6):e0276357. doi: 10.1371/journal.pone.0276357 (PMC10309596; doi:10.1371/journal.pone.0276357)
Supplement: S1 File — (ZIP) [file pone.0276357.s001.zip › Supporting information /Health worker 7.docx]

In-depth interview questionnaire for health workers

**Introduction and Consent**

Hello, my name is Abdourahman Bah. I am a final year (MRC sponsored) BSc Global Health student at Queen Mary University of London. I am interviewing health workers and mothers in The Gambia to learn about the impacts of Covid-19-related lockdown measures on utilisation of mother and child services. The interview will take about 30 minutes. All the information I obtain will remain strictly confidential. You may choose not to answer any question that makes you feel uncomfortable.

Do you have any questions?

Do you agree to being interviewed? Yes

| **Background** |
| --- |
| 1. **Could you please tell me where you live?**   I live in Brikama Manduar. It is quite far from here. I used public transport to come to work.   1. **What is your profession?**   I am a nurse mid-wife.   1. **What does your role entail?**   My role as a midwife is to help people, more especially pregnant women, to be able to go through their pregnancies in a positive way. So as to help them avert any complications that may arise and can be prevented. I also do conduct deliveries but as in this hospital, I am working in the RCH, where I don’t conduct deliveries. I provide antenatal services. |
|  |
| 1. **What motivated you into pursuing a public health career?**   For me midwifery is a very nice and interesting field because as a midwife, you are a doctor of your own domain. You can do a lot. You can take care of yourself as a woman; you can help somebody to achieve her healthy status form pregnancy, childbirth and even during the postpartum and the adolescent and adult state. You can even help young girls in your area and even old people, you will be able to help them. Also, as a midwife, with that midwifery skill, you can help people in your community. Even by looking at someone working by, you can tell me if she is pregnant or not. You can also help those you are living with. |
| 1. **What MCH services are provided in this facility? Probe: immunisation, antenatal care**   We conduct deliveries, provide antenatal services, family planning services, we see children under 5. VIA screening is also done in this facility for non-pregnant women. If they are pregnant, we ask them to come for the VIA screening after three months. We also provide male clinics, which is part of the antenatal services we provide because we deem it necessary to talk to them. We have seen its impact. We sensitise them and talk to them about health issues related to pregnancy. The prevention of mother to child transmission of HIV service is also provided here.   1. **Did the provision of these services continue during the pandemic?**   Yes, part of the other services were stopped: the male clinic was stopped for a period of time and even the postnatal clinic was stopped for a period of time but then after it was started because we see that Covid-19 is still here and we taught it was going to last just for some months but it has now been over a year. So, we cannot say it is because of Covid-19 that we have to stop everything. These kids under 5 years of age, maybe for the male clinic you can compromise that but for the postnatal clinic you cannot compromise that because you have neonates that are sick and for some mothers like me, maybe you don’t know your child may not be breastfeeding and you see baby’s skin changing and you just say I have been told not to go the hospital. The child may stop feeding and just die in your hand and when you bring the baby to the hospital, you just say the bay was not breastfeeding. if that child was brought here earlier on, something could have been done. |
|  |
| 1. **Have you noticed any changes in utilisation of MCH services during the pandemic? For example, do you see fewer or more patients than usual?**   Here, to be honest, we tried to reduce the number of women coming for antenatal services, but we couldn’t because they come and you cannot send them home and they are already booked, though the booking for patients has been reduced, but the re-visiting remained the same. The only thing was that we emphasised that they put on their mask and when they enter that door, even us the hospital staff, the security ensured that you wash your hands before you enter the health facility, but the number of people coming for antenatal services was not reduced, compared to other health facilities. I have heard that in other hospitals like Serrekunda and Fagikunda, they only take twenty to thirty patients per day. The rest they just tell them to go home. Maybe the ones they sent home maybe having a health problem and the ones they are seeing may not have any health issues. So, how will they know who has a health problem and who doesn’t. so, it is better you just keeping seeing every patient that comes to the hospital, and emphasise hand washing, wearing of face mask and observing social distancing. That is, to comply with the protective measures and do your work. So, I can say that here the number of people coming for antenatal services was not reduced because I was here last year, and it was not reduced and is still the same. |
|  |
| **Individual factors** |
| 1. **From the perspective of health workers, how safe do you think it is to provide MCH services during the pandemic?**   To be honest, it was not safe, but we are health workers. Even if everybody stays at home, health workers, especially nurses and midwives, cannot stay at home. We have to come and work. I have told where I come from, from Manduar to this hospital and even during that time, I used to pay extra costs because the taxi in which I used to pay ten dalasi, they increased it to twenty dalasi because they used to carry only two people in the vehicle. For the local van, instead of 18 dalasi, they would charge me 50, 35, 30 dalasi as they didn’t have a fix price. I still had to manage and come because it is our job and it is our responsibility to make sure that we help them and we also do our part, though at that time, the government announced the introduction of scaling down of health workers. We did that but even with that those who stay do it weekly of fortnightly. So, we just managed |
| 1. **How safe is for women to access MCH services in this facility at that period?**   To be honest, it was not safe for them either. They too they knew that they are pregnant, and they should attend their clinics. So, they had to come because of that, but to be honest it was not safe, and we have seen women with Covid-19 and we still see some here. Some of them will not even know that they have Covid-19. For example, just a few weeks back, one was here and after palpating everything, I said to her that I can even feel that you have a temperature, and you are constantly coughing. So, can you go and report to the Gynaecology clinic. When she got there, a nurse asked her to go and do the rapid test and she come back positive. so, already I came into contact with her and how many came into contact with her since she left her home, and she might have spent more than 30 minutes here. So, how many mothers may have come into contact with her. So, it was not the least safe. So, we just had to manage. |
| 1. **Did you or your colleagues work more or less hours during the lockdown? If yes, please explain why?**   To me, it was the same time. We are supposed to close at 2:00 pm on Fridays but we sometimes stay here until 3:00 to 4:00 pm. Depending on the flow of patients, if you finish early you go home. |
| **Interpersonal factors** |
| 1. **What is your family’s attitude in your provision of MCH services during the pandemic? (Are they supportive or not? If yes, explain how?**   For me, I didn’t not have any support because I pay my own fare. Nobody gives me anything. It was kids who used to tease me because I used to tell them not to go out and if they come home, they should wash their hands. So, when I also come home from work, they would tell me to go straight to the bathroom and put my things aside. They would also tell me not to get close to them, so, that was the only thing, other than that, there was no other difficulty that I experienced. however, I was stigmatised because of my occupation. Even in my own community I was stigmatised. For example, there was a day I went to visit a woman in my community who just gave birth. So, I when I went there, another woman told me hope you have not brought along the nurses’ sickness. Another woman also asked me if I believe in the existence of Covid-19 and I told her that I believe that it really exists and I have seen somebody with Covid-19, but if you don’t believe it is not my problem but for me, I believe it. She kept saying that no, Covid-19 is not real. This is what many people believe here and up to today that what many people believe. |
|  |
| 1. **What is your attitude towards MCH service users during the pandemic? probe: were they making your work easier or more difficult?**   To be honest, it was difficult because some of them refuse to wear face mask and refused to observe social distancing. Even if you tell them to keep a safe distancing, they will just ignore you. If you also ask them to wear a face mask, they will just put it over their mouth without covering the nose and they would remove it as soon as you leave. So, it was really difficult. As such, at times, I just ignored them. When they come in for palpation, I ask them to put on their face mask. I don’t allow them to come into to consultation room if they don’t wear a face mask. |
| **Community factors** |
| 1. **Have you experienced any changes in people’s perception in the community about the use of MCH services during the pandemic? if yes, explain.**   Yes, even if they fall sick, they refuse to go to the health facility. They would say that if you go, they will say that you have Covid-19. There was a day I told somebody, who was sick and was no willing to go to the hospital because of the fear that she will be told that she has Covid-19. I told her that the Covid-19 testing doesn’t work like that and you will not even be tested today and get the results on that same day because there was not rapid testing at that time. this fear made many people not to go to health facility during that time, even if they are severely sick. |
|  |
| **Institutional factors** |
|  |
|  |
| 1. **Do you think this health facility had adequate medical supplies during the pandemic? if no, give reasons.**   We didn’t not experience a huge shortage of medical supplies at that time. it was only some medicines that are used for the family service were not available and are still not available. I cannot tell if this is because of the pandemic because we used to have shortages even before the pandemic. So, during the pandemic last year, we didn’t have that much of a shortage. So, I can’t say it it is because of the pandemic.   1. **Do you think this health facility had adequate PPEs during the pandemic? if no, give reasons. Did that have any effect on your willingness or ability to provide MCH services?**   Yes, we used to experience a shortage of PPEs at that time. For the face mask, we improvised by using the locally made face mask until we received donations. For gloves too, there was a time when there was a shortage, but it didn’t take that long. The shortage of gloves didn’t disturb us that much because we don’t conduct deliveries. We only use it in the family planning service when doing other procedures such as insertion, but for the injections we just use our bare hands and we them after attending to each patient. |
| 1. **Do you think this facility had enough manpower to provide MCH services during the pandemic? if no, give reasons**   Yes, during the pandemic, we had a shortage of manpower because some of our colleagues were sick, and others were diagnosed with Covid-19 and had to quarantine for two weeks along with those who came in contact with them. This affects our service delivery. The workload also increased for those of us who remained because we had to cover for those were absent. This made our work very difficult at that time. |
| **Policy factors** |
|  |
| 1. **To prevent the decline in use and provision of MCH services in the event of another pandemic or second wave, what do you think the government should do?**   In my opinion, the government should learn more because when the pandemic started, we all saw what happened and it is still a problem. The place that call is a treatment centre is not suitable to be a treatment centre. So, the government should put in more effort in building a proper hospital for treating Covid-19 cases. When it comes to nurses, our welfare is really compromised by the government. We are not considered. We are much paid less than others. We are the frontline health workers, who come into contact with positive cases. We get infected and take it to our families and to ourselves. When you infect your child or your husband or another relative, they easily die from the disease. So, they are not supporting us in any way. No allowance, no incentives in fact we don’t receive any support from them. Since we receive that allowance in 2020, we have not received anything after that. Each nurse should be given a package and vitamin C to boost your immune system. Everybody is not the same but even our facilities, sometimes they don’t have oxygen and other basic things and even nurses are not enough. All those who are trained health workers, where are they. For me the government is not doing much. They are trying but they still need to improve   1. **What advice would you give to people who are not using MCH services during the pandemic?**   At times it will be difficult, as I said earlier, some believe in that Covid-19 is real and others don’t. For me, what I always advise them, even when they come here and ask me if they should come for their next appointment, I always tell them that whenever we give you an appointment make sure you come because it is very important. You cannot sit at home and say I will go to your appointment because of Covid-19. So, whether there is a third wave of forth wave, maternal health service should not be compromised. When they are given an appointment, they should report to the health facility. The only thing is that they should abide by the precautionary measures such as wearing of face mask and observe social distancing. Also, if they don’t have an important thing to do outside, they should stay at home. |
