# Supplementary material for: Factors that influenced utilization of antenatal and immunization services in two local government areas in The Gambia during COVID-19: An interview-based qualitative study
Source: PLoS One. 2023 Jun 29;18(6):e0276357. doi: 10.1371/journal.pone.0276357 (PMC10309596; doi:10.1371/journal.pone.0276357)
Supplement: S1 File — (ZIP) [file pone.0276357.s001.zip › Supporting information /Respondent 11.docx]

In-depth Interview Questionnaire for MCH service Users

**Introduction and Consent**

Hello, my name is Abdourahman Bah. I am a final year (MRC sponsored) BSc Global Health student at Queen Mary University of London. I am interviewing health workers and mothers in The Gambia to learn about the impacts of Covid-19-related lockdown measures on utilisation of mother and child services. The interview will take about 30 minutes. All the information I obtain will remain strictly confidential. You may choose not to answer any question that makes you feel uncomfortable.

Do you have any questions?

Do you agree to being interviewed? Yes

| **Background** |
| --- |
| 1. **What is your ethnicity?**   I am a Fula   1. **What is your religion?**   I am a Muslim   1. **What is your marital status?**   I am married   1. **Could you please tell me where you live – Probe: house of residence is?**   I am currently from Bundung, but I live in London Corner. |
| 1. **Please tell me how you got here today? Probe: public transport, private or walked.**   I got here today by walking, but I usually use public transport. |
| 1. **Have you used MCH services during the pandemic? if yes, what MCH service have you used during the pandemic?**   Yes, I used to take him for immunisation in Bakoteh, where were living at that time. He was even born in Bakoteh. During the pandemic, I used to take him for immunisation in Bakoteh. |
| 1. **Have you changed the way you access this service during the outbreak? If so, how? If you have changed, are you going more times or less times and if so, what are the reasons? Probe-economic? Fears?**   During the pandemic, I was not taking him for immunisation every month because at that time, the health workers used to give us appointments. At that time, you only take your child for immunisation when you have an appointment, which was about every two months. This was because they stopped weighing children during the pandemic. So, I used to bring him only when he was supposed to get vaccinated. |
|  |
| **Individual factors** |
| 1. **How safe do you think it is to access MCH services during the pandemic? - Probe: have these concerns stopped you from using these health facilities?**   During the pandemic, I used to wash my hands before leaving the house and put on a face mask whenever I brough my child for immunisation. I would also wash my hands when I get to the hospital. It was because of the fact that it was not safe during the pandemic that they stopped weighing children and asked mothers to come only when their children were to be vaccinated. This was also to done to prevent over-crowding in the hospital which increases the risk of transmission. |
| 1. **Have you experienced any financial difficulties (e.g., transport costs) in accessing MCH services during the pandemic? if yes, explain. Probe- have these difficulties stopped you from using these health facilities?**   I did not experience transport difficulties during the pandemic. However, I have to note that they increased transport fares during the pandemic, but that did not prevent me from bringing my child for immunisation whenever I had an appointment. We also used to observe social distancing whenever we boarded a vehicle. |
| **Interpersonal factors** |
|  |
| **19.Have you noticed any changes in your friends’ attitudes in use of MCH services during the pandemic? probe: are they going more times or less times?**  I used to go for immunisation with one of my friends, but she later stopped taking her child for immunisation because she was afraid of getting infected as she felted that hospital was not safe since one can easily get infected there. |
| **Community factors** |
| **20.Have you noticed any changes in people’s perception in your community about the use of MCH services during the pandemic? if yes, explain. Probe: give examples of people being afraid of visiting facilities due to stigma associated with visiting health facilities or fear of being quarantined etc.**  I have not paid much attention to that. During the pandemic, I was always in my house. I hardly went out. So, I was not aware of the things that were happening in my community. However, whenever I brought my child for immunisation to the hospital, I noticed that there were not many people coming when compared to pre-pandemic attendance. I think many people were afraid of going to the health facilities during the pandemic. |
|  |
|  |
| **Institutional factors** |
| **23.Did the health facilities stay open during the pandemic? if no, state how this may have affected your access to MCH services.**  The hospital where I was taking my child for immunisation during the pandemic was never closed. However, they used to fumigate the hospital every morning. So, if you come to the hospital at that time, they would not allow you enter. So, for me, I used to come a bit late so that I would find them done with the fumigation. In that hospital, they introduced a limit to the number of people that they could attend to every day. So, whenever we come, they would give us tickets and ask you to wait outside the hospital. They would then call us in one after the other, so that the hospital would not get overcrowded and would ensure that we observe social distancing. |
|  |
| **25.Do you think this health facility had adequate medical supplies during the pandemic? if no, give reasons. Probe- has this stopped from visiting health facilities.**  Whenever I brought my child for immunisation, they would sometimes give me all the medicines that I needed but they would only give me some and ask me to buy the rest from the pharmacy. However, they would sometimes tell you that they do not have any of the medicines that you needed. so, you would have to buy all of them from the pharmacy. I don’t think that was because of the pandemic. That is something normal in this country. In the Gambia, the hospitals have only paracetamol. The other medicines are only available in the pharmacies or in private hospitals. |
|  |
| **27.What are your perceptions about the health workers in this facility? (e.g., competence or behaviour of health workers). probe- has this stopped you from visiting health facilities.**  As you know, some health workers are difficult to deal with. Every health facility should introduce the idea of issuing tickets to all of its patients. Since many health facilities do not do this, there are always problems with who should be attended to first. If, however, this is introduced, this problem would not exist anymore because if this is introduced people will be attending to according to the numbers on their tickets. Also, when it is your turn to be attended to, some health workers would sometimes ask you to go out and wait until whenever they want to attend to you. I have experienced this several times when I was pregnant. In one of these occasions, I went for antenatal care in Sukuta Health Centre. When I got there, I did not know when I should be attended to. As such, I went inside the room where they attend to women who come for antenatal care. I asked one of the health workers when should I be attended to, but she asked me to go out of the room because according to her she was busy. I told her that this is not the way they should treat pregnant women. When some women are pregnant, they are always stressed. The health workers should understand their situation and not to be harsh to them. This is why stopped going to that hospital and transferred to Bakoteh Health Centre. This was however reduced during the pandemic because there were few people coming to the hospital that time. The health workers also ensured that we observed social distancing which also reduced the confusion on who should be attended to first as we they would usually call us one after the other during the pandemic. |
|  |
| **Policy factors** |
|  |
| **30.To prevent infection in health facilities, infection prevention and control measures, such as mandatory screening, wearing of facemask and social distancing, have been introduced in many health centers. What is the effect of these measures on your use of MCH services during the pandemic?** |
| For me, personally, I am Asthmatic. Whenever I brought my child for immunisation during the pandemic, I would put on my face mask covering only my mouth and I would not cover my nose to allow me to breath properly. When I get into the hospital, they would sometimes ask me to cover both the mouth and the nose, but I would tell them that I would really want to cover both the mouth and nose, I can’t because if I do, I would have breathing problems. When I first came to this hospital, the security guard asked me to put on my mask but told him that I am Asthmatic. He understood my situation and allowed me to get into the hospital.  **32. What do you think is the effect of these measures on other people’s willingness to come for MCH services?**  I know this could have, however, prevented some people from coming to health facilities. I have experienced this in my house. There was a someone I was living with who got sick and was coughing a lot. I told him to go to the hospital, but he told me that if he goes to the hospital, they will say that he has Covid-19 since he was always coughing. He was not the only one who stopped coming to health facilities. Many people stopped coming to health facility during the pandemic. |
|  |
| **35. What do you think the government should do to prevent a decline in use of MCH services in the event of another pandemic?**  The government should help us in providing enough medicines. I know that they are trying as much as they can, but I will just encourage them to try even more. Buying medicines from the pharmacy whenever you come to the health facility is difficult for many people. Sometimes you would come to the health facility without having any money on you. Not everyone can afford to buy medicines from the pharmacy as we are all in different situations. They should also help us in improving the quality of service provided in public hospitals. Many people are now going to private hospitals because of the poor service that is offered in public hospitals. However, not everyone can afford to go to private hospitals.  **36. What advice would you give to people who are not using MCH services during the pandemic?**  If you are given an appointment, you should leave whatever you are doing and take your child for immunisation. You should prioritise the wellbeing of your child over everything else. If your child is not vaccinated, he/she will experience the consequence of that in the future. This will affect his/her health status later in life. This the reason why many children end up developing some illnesses later in their lives. If they are vaccinated, they will be safe from vaccine-preventable diseases. |
