# Supplementary material for: Factors that influenced utilization of antenatal and immunization services in two local government areas in The Gambia during COVID-19: An interview-based qualitative study
Source: PLoS One. 2023 Jun 29;18(6):e0276357. doi: 10.1371/journal.pone.0276357 (PMC10309596; doi:10.1371/journal.pone.0276357)
Supplement: S1 File — (ZIP) [file pone.0276357.s001.zip › Supporting information /Respondent 9.docx]

In-depth Interview Questionnaire for MCH service Users

**Introduction and Consent**

Hello, my name is Abdourahman Bah. I am a final year (MRC sponsored) BSc Global Health student at Queen Mary University of London. I am interviewing health workers and mothers in The Gambia to learn about the impacts of Covid-19-related lockdown measures on utilisation of mother and child services. The interview will take about 30 minutes. All the information I obtain will remain strictly confidential. You may choose not to answer any question that makes you feel uncomfortable.

Do you have any questions?

Do you agree to being interviewed? Yes

| **Background** |
| --- |
| 1. **How old are you?**   I am 22 years old   1. **What is your ethnicity?**   I am a Fula   1. **What is your religion?**   I am a Muslim   1. **What is your marital status?**   I am married   1. **Could you please tell me where you live – Probe: house of residence is?**   I live in Manjai |
| 1. **Please tell me how you got here today? Probe: public transport, private or walked.**   I got here today by using public transport |
| 1. **Have you used MCH services during the pandemic? if yes, what MCH service have you used during the pandemic?**   Yes, during the pandemic, I was pregnant. I used to come for antenatal care. |
| 1. **Have you changed the way you access this service during the outbreak? If so, how? If you have changed, are you going more times or less times and if so, what are the reasons? Probe-economic? Fears?**   I used to come for antenatal care during the pandemic regularly. In fact, I used to come for antenatal care every month. Even though I live far from the health facility, I did not miss a single appointment throughout my pregnancy. I used to travel from Manjai to this hospital every month even during the peak of the pandemic. |
|  |
| **Individual factors** |
| 1. **How safe do you think it is to access MCH services during the pandemic? - Probe: have these concerns stopped you from using these health facilities?**   It was not safe, but I just put my faith in God and come regardless of the Covid-19 situation. If you are sick, you would go to the health facility. If you are pregnant, you have to go for your appointments as long as the hospital remains open. Not coming for your appointment because of the pandemic would only harm you in the long run. You may develop complications during that time, and you would know unless you come regularly for your appointments. |
|  |
| **Interpersonal factors** |
| **18.What is your family’s attitude, including your husband, in your use of MCH services during the pandemic? Probe: Do they encourage or discourage you? In what way?**  My family was very supportive, especially my husband. He was the one who would tell me to go for antenatal care. When I forget about my appointment, he would always remind me. |
|  |
| **Community factors** |
| **20.Have you noticed any changes in people’s perception in your community about the use of MCH services during the pandemic? if yes, explain. Probe: give examples of people being afraid of visiting facilities due to stigma associated with visiting health facilities or fear of being quarantined etc.**  I did not pay attention to that because where I live, everyone is in their house. So, is difficult to know what is happen there or what people are saying about the pandemic. I, for one, I was not going out at that time. I would only go out for important things such coming to the health facility or going to the market. So, I am not a position to say anything about that. |
|  |
| **22.Have you experienced any challenges on getting to health facilities during the pandemic? if yes, state them (e.g., lack of transport)**  For me personally, I did not experience transport difficulties. However, I have to note that transport fares got increased at that time because all taxi drivers were told to make sure that they maintain social distancing in their vehicles. So, instead of taking four passengers in a taxi, they had to take only two to three passengers. This was not a problem for me as long as I could afford it. The problem that I experienced was that the waiting time was very long. We used to spend a long time here. There were many people coming for antenatal care. So, it takes a long time before the health workers could attend to all those people. Also, we used to spend a lot of time at the hospital. This because they security guards would ensure that everybody washes their hands at the gate and put on a face mask. They also ensure that people would not enter in groups. We would enter one after the other. All these measures led to increased waiting time. |
| **Institutional factors** |
|  |
|  |
| **25.Do you think this health facility had adequate medical supplies during the pandemic? if no, give reasons. Probe- has this stopped you from visiting health facilities.**  Sometimes, I would have all the medical supplies I need when I come here, but not always. Sometimes, they would write me a prescription and I would have to buy at the pharmacy. This, however, is not because of the pandemic. This is something normal in The Gambia. |
| **27.What are your perceptions about the health workers in this facility? (e.g., competence or behaviour of health workers). probe- has this stopped you from visiting health facilities.**  I did not any problem with the health workers. I followed all of the measures that they introduced in the hospital. Whenever they asked people to put on a mask, I would put it on. Whenever they asked people to observe social distancing, I would always do that. That is why I never had a problem with them. They had problems with those who refused to follow the measures that they introduced. |
| **28.Do you think the health workers were following the Covid-19 precautionary measures appropriately? For example, were they always wearing face mask and PPEs? Probe-has this stopped from visiting health facilities?**  Yes, they were following all the Covid-19 precautionary measures. They would always put on a face mask, and they would wash their hands regularly. |
| **Policy factors** |
| **30.To prevent infection in health facilities, infection prevention and control measures, such as mandatory screening, wearing of facemask and social distancing, have been introduced in many health centers. What do you think of the implementation of these measures in the health facilities? Probe: were they implemented correctly?**  Yes, when we come here, the health workers would always tell us to put on a face mask, wash our hands regularly and observe social distancing. So, we also ensured that we followed these measures correctly. We would always put on a mask and wash our hands at the hand washing stations provided in the hospital. They would not allow anyone to enter the hospital without a face mask. If you don’t have a mask, they would ask you to buy one before they would allow you the enter the hospital. If you can’t provide a mask, you would have to go home. |
| **31.What is the effect of these measures on your use of MCH services during the pandemic?**  Putting on a face mask was not something that I felt comfortable doing, but I had to put it on because I had no other choice. This did not prevent me from coming for antenatal care, but I have to admit that putting on a face mask was very difficult for me because when I put it on for a while, I start having breathing problems. Nonetheless, I had to beer the discomfort and come to the hospital because I knew it would last only for few hours. |
| **35. What do you think the government should do to prevent a decline in use of MCH services in the event of another pandemic?**  The government should help us to provide adequate medical supplies. We really need the government’s help in terms of providing enough medicines because one should not come for antenatal care without having the necessary medicines for your pregnancy.  **36. What advice would you give to people who are not using MCH services during the pandemic?**  The pandemic should not prevent anyone from coming for antenatal care at the health facilities. Anyone who is pregnant should come for their appointments regularly. This ensure that they go through their pregnancy without developing any complications and even if they do develop complications, these will be identified immediately before it could develop into a severe complication. |
