# Supplementary material for: Factors that influenced utilization of antenatal and immunization services in two local government areas in The Gambia during COVID-19: An interview-based qualitative study
Source: PLoS One. 2023 Jun 29;18(6):e0276357. doi: 10.1371/journal.pone.0276357 (PMC10309596; doi:10.1371/journal.pone.0276357)
Supplement: S1 File — (ZIP) [file pone.0276357.s001.zip › Supporting information /Health worker 12.docx]

In-depth interview questionnaire for health workers

**Introduction and Consent**

Hello, my name is Abdourahman Bah. I am a final year (MRC sponsored) BSc Global Health student at Queen Mary University of London. I am interviewing health workers and mothers in The Gambia to learn about the impacts of Covid-19-related lockdown measures on utilisation of mother and child services. The interview will take about 30 minutes. All the information I obtain will remain strictly confidential. You may choose not to answer any question that makes you feel uncomfortable.

Do you have any questions?

Do you agree to being interviewed? Yes

| **A** |  |
| --- | --- |
| 1 | **Could you please tell me where you live?** |
| 2 | Could you please tell me what your profession is?  I’m a public health officer. I have been a public health officer since August 2020. I graduated in 2018 but wasn’t employed until August 2022. |
| 3 | **What does your role entail?**  Public health is dynamic. We have so many roles to do which I even cannot remember all at once. Among some of the things we do include immunisations, birth registration, inspection and surveillance, but the functions of a public health officer go beyond that, whereby you have occupational health, environmental health, poor sanitation and international health regulation and so on and so forth. |
| 3 | **Please tell me for how long you have been working in this health facility?**  I have been working here for nine months now |
| 4 | **What motivated you into pursuing a public health career?**  It is about the three Ps of public health. I just want to prevent, protect, and promote lives. |
| 6 | **Did the health facility stay open during the pandemic, and for how long?**  Immunisations were conducted but at a different sequence. People were coming at random based on your convenience but now it depends on appointments and the capacity of the health facility. So many things were observed including social distancing, personal hygiene, wearing of face masks. Some of the key intervention strategies one can take in order to reduce the prevalence or spread of Covid-19 virus and the infection rate between one to one were also observed. Health promotion and talking to women about the signs and symptoms of the virus. I believed that they understood them very well. |
| 7 | **Have you noticed any changes in utilisation of MCH services during the pandemic? For example, do you see fewer or more patients than usual?**  In the utilisation of MCH services, there was an observed change because every month our target for the number of children to be immunised was over seven hundred, but during the pandemic we noticed that the number of children being brought for immunisation was far less than our target. This was due to notion people have, their attitudes and perceptions about Covid-19. the restrictions of human movements and banning of public gatherings also contributed seriously to the low coverage were experienced. Even talking about the OPD, the number we were seeing was very low. We can attribute all these to Covid-19. However, it is not about the pandemic itself, but sometimes it is about people’s level of understanding and their perceptions about the disease which maybe a contributing factor to the changes in their behaviour. I think, overall, it is all because of the pandemic as everybody is afraid. |
| **B** | **Individual factors** |
|  |  |
| 8 | **From the perspective of health workers, how safe do you think it is to provide MCH services during the pandemic?**  In a situation like this, even those that do not have much technical now how are always very mindful. It is always difficult to prepare for something that you don’t know about. If they know the covid-19 guidelines, they are well prepared because everybody will be wearing a face mask, applying hand sanitisers and putting on gloves and observing social distancing from home to work and from work to home. Let me take an example from myself, hardly during that time you see me with people after work because I know I’m at high risk of contracting the disease since I’m always in contact with positive cases. We are the front liners and as such I always take precautions. I believe that is how they do it. |
| 10 | **Did you or your colleagues work more or less hours during the lockdown? If yes, please explain why?**  The working hours was never less, because if you are to see hundred people per day, based on the timeline you are creating, you have to stay extra hours to satisfy your patients and for you to see the benefit of what you are doing you have to put in extra effort and that will cost you more time. At that time, everybody was at stand by. During an emergency period, you don’t have any time off and as such you are always on standby, and you therefore work more. You can just get home from work and then get a call to come back to work. Let take an example of what happened to me a time back. I thought I had the weekend off, and early in the morning, I invited a friend over. I left him there and went to the shop when I came back, I saw a missed call from my boss who told me a driver I have sent a driver to bring you to work. At that time, I used to come home from work at three. |
| C | **Interpersonal factors** |
| 11 | **What is your family’s attitude in your provision of MCH services during the pandemic? (Are they supportive or not?**  It all depends on how you conduct yourself and how you perceive certain things. If you perceive things to be very hard, your people will perceive it in the same manner. Being a health worker, so many people are relying on you. They take you as an example, you should be someone who will carry their burden. You should also help in counselling them. So, if you perceive it as a disease that cannot be cured and you can contract it by just looking at it, they will also perceive like that. So, I go to work knowing that I’m at risk of contracting the disease and I spend very little time with them. I also make sure that I keep some distance away from them. It was a very difficult time, but they were very supportive, but they didn’t actually know what my role entails. |
| 12 | **Have you noticed any changes in your colleagues’ attitudes in providing MCH services during the pandemic?**  I know for certain there were some who were not willing to provide MCH services during the pandemic, but I haven’t come into contact with anyone of them. |
| D | **Community factors** |
| 14 | **Have you experienced any changes in people’s perception in the community about the use of MCH services during the pandemic? if yes, explain.**  Well, at the beginning of the pandemic, some students and i started something in our community. I called it house-to-house sensitisation. We went to house-to-house to educate them about the signs and symptoms, complications, incubation period and mode of transmission and some of the key interventions they should take. From there, I think their attitude changed towards covid-19, which would reflect automatically on their willing to use maternal health services. I remember there was a time when you tell someone you should go to a health facility when you have a headache or fever, they would tell you I will not because if I went, they would tell me I have Covid-19 and I will be quarantined. There was this Covid-19 phobia that people were running away from. They would say if you there I may have Covid-19. This denied many people from coming to the health facility. |
| 15 | **Have you experienced any challenges in providing MCH services due to transport difficulties? if yes, explain how**  That is obvious. Then I was going to Kotu. I was travelling for a distance of 18km every day. I only get access to the vehicle when I come to Brikama. I would come here early in the morning and the vehicle would take me to Kotu and bring me back to Brikama and then find my own way to my home. So, I can say there were transport difficulties. Looking at the Gambian setting, you come to realise that people are not just working to earn a living, but what I understand from the health sector, they are patriotic as they want to do something for their country, so that is what motivate them. It is not because there is a commission or an allowance that is motivating health workers. They are always happy with their work which is not because of the money. It is because of the simple fact that they want to make change. Fare is something and salary and allowances are something else. |
| E | **Institutional factors** |
| 17 | **What do you think of the quality of care provided by this health facility during the pandemic?**  Obviously, it will be affected. You know hospital management has to do with economics. So, if the hospital is not financially strong, it is something else. We realised that were not enough medicines. In the Gambia everyone is poor and if you are asking people to buy their medicines when there are lockdown and people are not earning as they use to and the government cannot provide for them it becomes a constraint. So, these are some of the constrains. We do not have the human resource capacity. |
| 18 | **Do you think this health facility has adequate medical supplies and PPEs during the pandemic? if no, give reasons.**  Talking of enough PPEs, we do not have enough PPEs. During the covid-19 pandemic, we were supplied with mask. We did not have enough PPEs, but we managed and improvised. We were well trained as health care professionals. Whether PPEs are available or not, we should be able to improvise. Not having a face mask should not restrict/prevent me from rendering my service. We must find other alternatives. We those working at the OPD, and outpatients’ departments were provided with PPEs. |
| 19 | **Do you think this facility has enough manpower to provide MCH services during the pandemic? if no, give reasons**  No, at the beginning of the pandemic, the rapid response team suffered. We lacked adequate mobility and adequate human resource. Some health workers were relocated to other units. So, to develop a new unit during the pandemic, some had to be removed from other units. But in this was only in the beginnings as some changes were introduced later. |
| F | **Policy factors** |
| 20 | **Did the lockdown measures, such as stay at home policies, travel bans, etc., put in place last year had any impact on your ability to provide MCH services during the pandemic? if yes, explain how.**  Not necessarily. At the beginning of the pandemic, I had mobility issues, but later on I was allocated a driver who would drive me from home to work. The problem was getting home from work. During the lockdown though, mobility was a problem. You realise that drivers stop work early due to the curfews. At certain point in time, we were closing after six and the ministry had to provide some of us with motor bikes, but not everyone was lucky to have it. |
| 23 | **Are there any other measures introduced either in the community or health facilities that have had an impact on provision of MCH services during the pandemic? (e.g., policy to close certain health facilities or scale back MCH service provision) if yes, please state them and explain how.**  Mobility as I mentioned earlier on is a problem. Motivation is another problem. Health workers are working without any motivations. They are allocated with allowances but that is not something that happens often. It does not happen every month. The health workers are not well motivated. PPEs and other resources are also not enough. The most challenging thing though is the lack of knowledge, attitude and practices of people towards the pandemic. It will be very difficult to convince people to practice certain things if they are not convinced that Covid-19 really exist. If I tell you covid-19 is a western disease and is not applicable to local Gambia, you will not be well convinced to wear a face mask. So, if you can tell me that I have not seen anyone who died from Covid-19 in my own country, so I don’t have to believe it. So do I expect you to believe if I tell you there is a covid-19 case in the Gambia. So, we have to accept it first. I know the knowledge is lacking in our country but there also very knowledgeable people whose attitudes are very poor. So, if the attitudes are poor, the practices must be also poor. |
