# Supplementary material for: Factors that influenced utilization of antenatal and immunization services in two local government areas in The Gambia during COVID-19: An interview-based qualitative study
Source: PLoS One. 2023 Jun 29;18(6):e0276357. doi: 10.1371/journal.pone.0276357 (PMC10309596; doi:10.1371/journal.pone.0276357)
Supplement: Attachment — Submitted filename: Response to Reviewers.docx [file pone.0276357.s003.docx]

Dear Editors,

Editor Comments:

1. Title: Barriers to accessing mother, new-born and child health services in urban Gambia during COVID-19: An interview-based qualitative study
   - Be clear on the objective of the study and be consistent throughout from title till conclusion
   MNCH vs antenatal and immunization, these terms are not interchangeable. For one, MNCH includes curative services.

**Response:** MNCH replaced with antenatal and immunization were applicable

- ‘New-born’ replace with ‘newborn’ throughout

**Response: ‘**New-born’ replaced with ‘newborn’ throughout the manuscript

- ‘barriers to accessing...’ or ‘factors that influenced utilization of…’

**Response:** ‘barriers to accessing’ replaced with ‘factors that influenced utilization of’ throughout the manuscript

- “The Gambia” versus “the Gambia” versus “Gambia” – choose one and be consistent

**Response:** The Gambia used throughout the manuscript

2. Abstract
   - Introduction: Good to avoid terms that are not supported by data “least developed…, least researched…” , low-income country with limited research on ..

**Response:** “ ….least developed and least researched countries” removed from the abstract

- Results: ‘.. relating to factors that were responsible for the reduction in the uptake of antenatal and immunisation services’ since the study did not measure if there was reduction or not of antenatal and immunization service uptake, a better phrase could be ‘… factors that influenced uptake (use) of antenatal and immunization services...’

**Response:** ‘.. relating to factors that were responsible for the reduction in the uptake of antenatal and immunisation services’ removed from the abstract.

3. Introduction
4. Methods

Data collection:

- Sample size, why 17 mothers? Did you expect saturation by then? Most were from the 2 hospitals and not so much from the 3rd public hospital and hardly any from the private practice. Was that intended or were you unable to get participants? In either case – this should be reflected in the discussion as limitation, and reasons why.

**Response:** the initial plan was to recruit as least 24 mothers, 6 from each health facility, but this was not possible due to difficulties in getting participants willing to be interviewed, especially in private health facilities. Most of them did not like the idea of being tape recorded even after telling them it would be totally anonymised. We also decided to include only one private health facility because we realised that the use of MNCH services in private health facilities were hardly disrupted. This limitation has been reflected in the discussion section (page 12, paragraph 5, line 13)

- How did you classify mothers who regularly versus not regularly used services? Were they asked different questions? Were there unique perspective ..? were you trying to see change from their norm in terms of use of service?

**Response:** mothers who accessed antenatal and immunisation services for at least 4 times during the pandemic were classified as regularly used services, while those who accessed services for less than 4 times were classified as not regularly used services. some of the questions they were asked were similar while some were different. We classified them into these categories so that we can understand the barriers/challenges that those who regularly used services experienced while accessing services and understand the reasons why some mothers did not regularly used services.

- Who conducted the interviews (were the interviewers trained, male/female, health worker/not hw)? Possible social desirability bias given that the interview occurred inside a health facility

**Response:** All the interviews were conducted by the first author, who was trained on interview techniques by the second author prior to conducting the interviews. This statement has been included in the methods section (page 9, second paragraph). The authors acknowledge that there could be a possible social desirability bias given that the interviews occurred inside a health facility. This statement has been added to the discussion section as a possible limitation (page 12, paragraph 5, line 12).

- How long did the interview take per person?

**Response:** The interviews lasted between 15-30 minutes. This statement has been included in the methods section (page 5, paragraph 2, line10).

- Were the tool used for interview translated/back translated or? dedicated translator or were the authors/interviewers fluent in all three languages?

**Response:** The first author is fluent in all the three languages. He conducted all the interviews and translated those conducted in Mandinka and Fula into English. As such, a dedicated translator was not needed. This statement has been included in the methods section (page 5, paragraph 2, line 9).

5. Results
   - ‘claimed’ has negative undertone suggest using words like ‘reported’ throughout

**Response: ‘**claimed’ replaced with ‘reported’ throughout the manuscript

- ‘Fear of being quarantined’ is this similar to being asked to stay at home and to social distance. Good to differentiate and clearly state.

**Response:** they are different. At the start of the pandemic, when someone tests positive, they would be taken to a government facility, usually a hotel, where they will stay at least for 14 days before they will be allowed to go home. This was later changed to allow those who test positive to self-isolate at home for 14 days. Changes made to the manuscript (page 7, paragraph 2, line 5)

- Given your small sample size per health facility, it is possible that the respondents might be identified by health facility name. We often advise to not include name of hospital or say hospital 1 or 2 to avoid possible identification of interviewee and breach of confidentiality.

**Response:** Hospital names removed from all quotes, only numbers used.

- Some of the interviewees were describing what they perceive was going on in their community – good to say that in your discussion section some of your findings were also the interviewees perspective of what was going on in their community and not a personal experience.
o “There were several people in my community who stopped taking their children for immunisation during the pandemic..”.

**Response:** This statement has been included in the discussion section (page 12, paragraph 5, line 15)

- Reference “WhatsApp”

**Response: “**WhatsApp” removed from the manuscript

- “misleading information” or “rumors” – trying to understanding if the respondents defined the information as “misleading’ or “rumor” ? as some of the health facilities were closed as described under paragraph Scaling down of MNCH services.

**Response:** both terms were used by participants to define the information.

6. Discussion
   - Hard to follow flow of this section. Suggest restructuring: main/key finding, interpretation of your findings/reflection of findings as it relates to other African and non-African studies, implications of the findings, strength/limitations of study, recommendation (what could be done differently in the future based on your findings).

**Response:** Discussion section restructured based on the above suggestions.

- First sentence
o Given the small sample and lack of generalizability in Gambia suggest editing to say gives insight in Urban areas of Gambia.

**Response:** Wording of the first sentence changed to “urban areas of The Gambia”

- Second sentence
o ‘Factors responsible for reduction’… suggest rewording… the study does not measure reduction but rather factors that influenced decision to use or not use services.

**Response: ‘**Factors responsible for reduction’ replaced with ‘factors that influenced the use’

- Line 8
o “physical barriers” .. suggest to say barriers

**Response: “**physical barriers’ replaced with “barriers”

- ‘small” country as compared to ?

**Response: “**small” removed from the manuscript

- Limitations: I would also think these were limitations
o The small sample size , unclear if saturation was researched
o The sampling method, selection bias as the pool of patients was drawn from hospital and does not include those who did not seek care at all. The authors acknowledge that there could be a possible social desirability bias given that the interviews occurred inside a health facility.
o Urban centric as sample was from urban areas.
o Hence, findings not generalizable to Gambia but provides insight for the country.

**Response:** these points have been included in the limitations (page 12, paragraph 5, line 10 – 19).

7. Figure 1. Missing title of Figure. Not sure it adds value. Is it trying to capture framework or key findings and needs language edits “mistreatment by ..”

**Response:** Figure 1. removed from the manuscript.

**Reviewer Comments to the Author:**

1. Intro comments
   • “since the health of women and children is usually disproportionally affected during pandemics and conflicts” (page 2) I have no doubt this statement is true but was wondering if you could cite evidence for this statement?

**Response:** We thank the above reviewer for highlighting this lack of clarity. We have decided to remove the above statement from the manuscript.

• Not mandatory but may be helpful for the reader- “The country has an under-five mortality rate of 56 per 1,000 live births, an infant mortality rate of 42 per 1,000 live births and a neonatal mortality rate of 29 per 1,000 live births [26]. Concerning maternal deaths, the maternal mortality rate is 289 per 100,000 live births [26]” (page 2) do you have any global estimates that you could compare to so the reader doesn’t have to look that up themselves while reading?

**Response**: Thank you for this suggestion. Here are some global estimates: global maternal mortality rate: 223 per 100,000 live births, under-5 mortality rate: 38 per 1,000 live births, infant mortality rate: 29 per 1,000 live births, neonatal mortality rate of 18 per 1,000 live births.

• Add when data in table 1 are from to the title of the table (I assume they’re from 2019 since they match numbers from 2019 in introduction section). I suggest to also reference table 1 when speaking about 2019 indicators

**Response**: Thank you for this additional reference. 2019 added to title of table 1 and table 1 referenced when speaking about 2019 indicators (page 2, paragraph 4 and 5).

• “The COVID-19 pandemic, which was declared a public health emergency in January 2020, reached the Gambia on 17 March 2020” (page 3) I suggest re-wording this to say that COVID-19 was first detected on 17 March 2020 since it may have been circulating in the Gambia before this

**Response**: Thank you for this suggestion. The wording of the above sentence changed to “The first case of COVID-19 was detected in The Gambia on 17 March 2020”.

• Edits on table 2
1- Suggest re-naming table 2 “COVID-19 Cases in the Gambia Over Time” or something similar so we know cases in your heading are in fact cases of COVID-19

**Response**: Thank you for this suggestion. Title of table 2 renamed as ““COVID-19 Cases in the Gambia Over Time” (page 3)

2- Make sure that all headings indicate that these are total #s of cases, deaths, etc.

**Response**: Thank you for this suggestion. Changes made as suggested.

3- Add a border to separate October to December 2020 and January to March 2021.

**Response:** We are a bit unsure about this recommendation, as you will notice there is already a border in the table. Please advise on further action.

4- In writing you state “by June 2020, only 48 new cases had been detected” (page 3) but table 2 says there were 45 cases April-June 2020, why are these numbers different?

**Response**: Thank you for raising this. 45 news cases between April and June + 3 new cases in March excluding the first case = 48 new cases by end of June excluding the first case. Wording has been changed to “45 new cases by the end of June” (page 3, paragraph 1, line 4).

5- Is this total cases in all of the Gambia or part of the Gambia, and is this all people in the Gambia or just the population of interest. Consider adding a footnote in your table to specify all of this. Is this limited to confirmed cases and is a confirmed case by PCR test only or antigen and PCR here?

**Response:** Thank you for highlighting this lack of clarity. It is the total cases in all of The Gambia. It is also all the people in The Gambia not just the population of interest. The data is limited to confirmed cases only. I am not sure how cases were confirmed but all the data used here are from the Ministry of Health reports. This clarification has now been added in Table 2 and in page 3, paragraph 1).

• “evidence collected thus far suggests that the uptake of 4 antenatal and immunisation services declined considerably during the first wave of the pandemic” (pages 3-4). When did the first wave end in the Gambia?

**Response:** Thank you for this question. The first wave ended in September 2020. This clarification has now been added in page 4, paragraph 1, line 4).

2. Materials and methods comments
   •What is the catchment area of these facilities? Are only people from the region of the facility or is it common for people in rural locations and outside of the region to visit one of these hospitals?

**Response**: Thank you for these questions. The health facilities are from the two most densely populated local government areas in the county, representing about 57% of the country’s population. With regards to antenatal and immunization services, only patients living in these areas access such services. This clarification has now been added in page 4, paragraph 4, line 2.

• Table 3:
1- Merge the two Kanifing LGA sub-headers so it looks like the Brikama sub-header

**Response**: Thank you for this suggestion. Merged the two Kanifing LGA sub-headers

2- Center the #s in the respective cells as is done with prior tables.

**Response**: Thank you for this suggestion. Centred the numbers in the respective cells

3- Highlight the second total column. Also suggest to label them differently so they can be easily distinguished to someone reading table without the accompanying methods section.

**Response**: Thank you for this suggestion. Changes made as suggested.

• “Following this, to ensure the objectivity of the findings, the two researchers worked closely together in reviewing and defining themes” (page 5) so did both reviewers code everything and compare or did each reviewer work on something different and discuss? Please clarify.

**Response:** Thank you for highlighting this lack of clarity. Both researchers coded everything and compare. This statement has been included in the data analysis section ( page 6, paragraph 1, line 9)

3. Results:
   • Results are presented in a cohesive manner- were there any instances where a single health facility or type (e.g. private vs public), or were all of these themes corroborated in all 4 facilities? E.g. was there a lack of PPE and meds in all facililies or just some? Public vs private differences may be noteworthy since public facilities in some low income settings are known to be more under-resourced

**Response:** Thank you for raising this. For some of the themes, they were corroborated in all 4 facilities, but that is not the case for every theme. When doing the analysis, we tried to find the most common themes among all the facilities, but that was not always possible since most of our participants, particularly the mothers, were drawn from only two health facilities. Public vs private differences were difficult to determine since we had only 3 participants from the private sector, but still, we noticed that the disruption on MNCH services was more common in the public sector compared to the private sector.

4. Discussion
   • From my understanding, another limitation is not knowing how mothers and healthcare workers impressions on accessing MNCH before COVID-19. Some of these issues you discovered may be new, but I am left curious if some existed before the pandemic or were relevant during other outbreaks. I do see you cited Ebola also playing a role in misinformation, but I am interested if Ebola had implications on other factors too… may not be possible to know, so would acknowledge what you don’t know about other outbreaks in limitations.

**Response**: Thank you for this suggestion. We understand that some of the factors highlighted by study participants existed even before the pandemic, but the participants highlighted that some of these factors got worse during the pandemic, such as lack of medications. With regards to the implication of Ebola, we don’t really now, so we have now added this as a limitation (page 12, paragraph 5, line 3)

5. Conclusions
   • “as well as to the need to counter misinformation” (page 13) would also add the need to mitigate fears of the public since this was a big theme of yours.

**Response:** Thank you for this suggestion. **“**the need to mitigate fears of the public” added to page 13, paragraph 3, line 4.

• Any more specific thoughts on what the facilities and government can do if there’s a future uptick or different pandemic? E.g. maybe recommend things like stockpiling extra PPE, planning for essential med logistics in a pandemic, etc.

**Response:** Thank you for this suggestion. More recommendations added based on the above suggestions (page 13, paragraph 3, line 4)
