# Supplementary material for: Factors that influenced utilization of antenatal and immunization services in two local government areas in The Gambia during COVID-19: An interview-based qualitative study
Source: PLoS One. 2023 Jun 29;18(6):e0276357. doi: 10.1371/journal.pone.0276357 (PMC10309596; doi:10.1371/journal.pone.0276357)
Supplement: Attachment — Submitted filename: Response to Reviewers.docx [file pone.0276357.s004.docx]

Dear Editors,

Editor Comments:

1. 1. Introduction section
   Keep the mortality information as it is relevant for readers and makes a point that in countries that have high infant, child and maternal mortality the indirect impact of the pandemic can be severe. Minor edit - it is Maternal mortality ratio (not rate).

**Response**: We have kept the mortality information as suggested and have changed Maternal mortality rate to maternal mortality ratio.

2. Discussion section
   "Temesgan and colleagues [14] found that mothers who were not required to obtain permission from their partners to access antenatal and immunization services during the pandemic had greater odds of accessing antenatal and immunization services than those who were required to seek permission" While an interesting finding I was not clear how the findings relate to the results of your study.

**Response**: the above citation has been removed from the transcript and replaced with: “Hailemariam and colleagues [18] found that mothers were unwilling to seek antenatal services due to fear of mistreatment by health workers and perceived poor quality of care arising from lack of motivation of health workers to provide antenatal services”.

**Reviewer Comments to the Author:**

1. Check throughout paper that you are using plural verbs when referring to data, since you have more than one piece of data (i.e. use “data were” rather than “data was”)

**Response**: Thank you for this suggestion. “Data was” replaced with “data were” throughout the article.

Abstract

2. “we identified five main themes, ranging from individual factors to interpersonal, community, institutional and policy factors”
   • Seems you identified more than five themes since each of these areas has more than one theme, suggest to instead say “we identified themes at five different levels: individual, interpersonal, etc” or something along this line for clarity.

**Response**: Thank you for highlighting this lack of clarity. Changes made as suggested (page 1, paragraph 3, line 1)

Intro

3. “With the recent COVID-19 pandemic, the achievements made globally in improving mother”
   • This statement implies that the pandemic is over, but there are still cases occurring globally,” consider rewording to “ongoing COVID-19 pandemic” or “COVID pandemic declared as a public health emergency on x date” or something similar.

**Response**: Thank you for this suggestion. “With the recent COVID-19 pandemic” replaced with “ongoing COVID-19 pandemic” (page 2, paragraph 1, line 1)

4. “During the early phase of the pandemic there was a concern about the potential”
   • very minor but would add comma after the word pandemic.

**Response**: Thank you for this suggestion. Comma added as suggested (page 2, paragraph 1, line 3).

5. “The demands-side factors include movement restrictions, economic hardship”
   • Minor but take out the s in demands here to keep language consistent.

**Response**: Thank you for this suggestion. The s in demands removed as suggested (page 2, paragraph 3, line 4)

6. “However, despite these achievements in increased MNCH uptake, when compared to international rates, the maternal, newborn and child mortality rates in The Gambia remain very high”
   • Suggest to remove the words “when compared to international rates” since you didn’t provide international rates in relation to MNCH uptake, this will leave the reader wanting international rates to compare to.

**Response**: Thank you for this suggestion. “When compared to international rates” removed as suggested.

7. You talk about MNCH and how it has improved over time, but then for mortality rates, you don’t mention change over time… is this because mortality hasn’t improved over time? Instead of focusing on mortality rates being high and not saying what they are high in comparison to (presumably it’s global rates but you don’t explicitly say this or what those global rates are), would focus on this rate not improving if this is true. I also recommend to consider why you even included mortality information in this paper if the focus is antenatal and immunization services. and threats to improvement of this. Suggest to add a sentence to drive home why it matters in the context of this paper or delete this paragraph.

**Response**: We appreciate your suggestion, but we have decided to keep the mortality information as the editor suggested.

8. “On 27 March, the country declared a state of emergency, which included the closing of schools, non-essential shops, and places of worship, the prohibition of social gatherings of more than 10 people, and the limiting of the number of passengers on public transport”
   • Did the state of emergency mandate these things? Suggest to change verb if this is what is meant.

**Response**: Thank you for this suggestion. Verb changed to mandated (page 3, paragraph 2).

9. “Between April and July 2020, the government introduced contact tracing and quarantine measures for suspected and confirmed cases, who were obliged to remain in hotels for 14 days.”
   • So quarantine meant they were obliged to stay in a hotel? If this is what you meant, suggest to reword to “and quarantine/isolation measures for suspected and confirmed cases which obliged cases to remain in hotels for 14 days” And what about contacts… did they need to quarantine in a hotel?

**Response**: Thank you for asking for clarification. Contacts were initially required to quarantine for 14 days, but later, quarantine in hotels was replaced with self-isolation at home for both suspected and confirmed cases.

10. Also, isolation applies to cases to prevent spread of illness and quarantine applies to contacts who are not yet ill, so I suggest to check that you use these terms correctly throughout discussion on quarantine/isolation.

**Response**: Thank you for this suggestion. We have made the following changes: “Between April and July 2020, the government introduced contact tracing and quarantine measures which obliged suspected and confirmed cases to remain in hotels for 14 days. As the pandemic progressed, hotel quarantine was replaced with self-isolation at home for both suspected and confirmed cases for a period of 10” (page 3, paragraph 2, line 7-9). With regard to your question about quarantine of contacts,

11. “To ensure confidentiality and anonymity, the interview data was anonymised.”
    • The use of anonymous twice is redundant, maybe say data were de-identified before analysis. Also make sure you refer to data as plural rather than singular here and throughout the paper.

**Response**: Thank you for this suggestion. “The interview data was anonymised” replaced with “data were de-identified before analysis” (page 4, paragraph 3)

12. “They were reported to have the highest prevalence of COVID-19 cases in the country [43].”
    • Just looked at this citation and didn’t see anything about prevalence of COVID-19, seems like you may have cited the wrong source.

**Response**: Thank you for bringing this to our attention. Our sincere apologies, the source reported the incidence not the prevalence in the second paragraph of its methods section. We have made the following change: “They were reported to have the highest incidence of confirmed COVID-19 cases in the country”.
